# Glucocorticoid receptor-induced non-muscle caldesmon regulates metastasis in castration-resistant prostate cancer

**DOI:** 10.1038/s41389-023-00485-z

**Published:** 2023-08-12

**Authors:** Verneri Virtanen, Kreetta Paunu, Antti Kukkula, Saana Niva, Ylva Junila, Mervi Toriseva, Terhi Jokilehto, Sari Mäkelä, Riikka Huhtaniemi, Matti Poutanen, Ilkka Paatero, Maria Sundvall

**Affiliations:** 1 Cancer Research Unit, Institute of Biomedicine, and FICAN West Cancer Center Laboratory, University of Turku, and Turku University Hospital, Kiinamyllynkatu 10, 20520 Turku, Finland; 2 Research Centre for Integrative Physiology and Pharmacology, Institute of Biomedicine, and FICAN West Cancer Center, University of Turku, Kiinamyllynkatu 10, 20520 Turku, Finland; 3 Turku Bioscience Centre, University of Turku and Åbo Akademi University, Tykistökatu 6, 20520 Turku, Finland; 4 Department of Oncology, Turku University Hospital, PL52, 20521 Turku, Finland

**Keywords:** Prostate cancer, Endocrine cancer

## Abstract

Lethal prostate cancer (PCa) is characterized by the presence of metastases and development of resistance to therapies. Metastases form in a multi-step process enabled by dynamic cytoskeleton remodeling. An actin cytoskeleton regulating gene, *CALD1*, encodes a protein caldesmon (CaD). Its isoform, low-molecular-weight CaD (l-CaD), operates in non-muscle cells, supporting the function of filaments involved in force production and mechanosensing. Several factors, including glucocorticoid receptor (GR), have been identified as regulators of l-CaD in different cell types, but the regulation of l-CaD in PCa has not been defined. PCa develops resistance in response to therapeutic inhibition of androgen signaling by multiple strategies. Known strategies include androgen receptor (AR) alterations, modified steroid synthesis, and bypassing AR signaling, for example, by GR upregulation. Here, we report that in vitro downregulation of l-CaD promotes epithelial phenotype and reduces spheroid growth in 3D, which is reflected in vivo in reduced formation of metastases in zebrafish PCa xenografts. In accordance, *CALD1* mRNA expression correlates with epithelial-to-mesenchymal transition (EMT) transcripts in PCa patients. We also show that *CALD1* is highly co-expressed with *GR* in multiple PCa data sets, and GR activation upregulates l-CaD in vitro. Moreover, GR upregulation associates with increased l-CaD expression after the development of resistance to antiandrogen therapy in PCa xenograft mouse models. In summary, GR-regulated l-CaD plays a role in forming PCa metastases, being clinically relevant when antiandrogen resistance is attained by the means of bypassing AR signaling by GR upregulation.

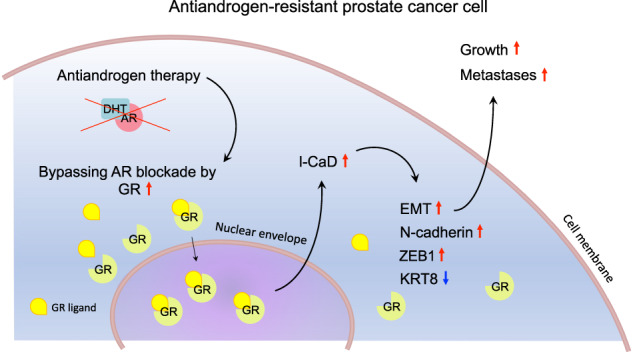

## Introduction

PCa is globally the second most common cancer in men [1]. While a substantial part of local PCa tumors are indolent, a subset of them are aggressive and cannot be curatively treated. Some patients are also diagnosed primarily with metastatic disease. In metastatic PCa, androgen deprivation therapy (ADT) and antiandrogens are used to inhibit the single most important driver of PCa progression—AR signaling. Although most of the patients respond initially, the cancer cells eventually adapt and develop resistance to therapies targeting AR signaling. Resistance to antiandrogens in PCa is currently understood to be attainable by mechanisms leading to restored AR signaling, such as AR amplifications, and other AR-targeting mutations or changes in the adrenal and intratumoral steroid synthesis; AR bypass signaling, such as GR upregulation; and complete AR independence [2]. Further knowledge of these mechanisms is of clinical importance and may uncover potential targets to address antiandrogen resistance in PCa.

Regulation of actin cytoskeleton has a central role in the formation of metastases [3]. *CALD1*-gene encodes two major molecular weight isoforms that regulate actin cytoskeleton by direct actin-binding: high-molecular-weight CaD (h-CaD, 120–150 kDa) and l-CaD (70–80 kDa) [4, 5]. Both isoforms produced by alternative splicing share the currently known functional regions, including regions for binding actin, tropomyosin, calmodulin, myosin, and phospholipids [6, 7]. h-CaD, also known as smooth muscle CaD, is a contractility regulator highly expressed in smooth muscle cells [8]. h-CaD is also expressed in the prostatic stroma, including vascular endothelial cells [9]. l-CaD, on the other hand, is a ubiquitously expressed protein localized in membrane ruffles and lamellipodial extensions of migrating cells and associates with the regulation of microfilaments by actomyosin cross-linking and actin polymerization [5, 10–13]. The effect of l-CaD on contractility and migration, however, seems to be two-fold, as both the loss of and increased levels of l-CaD are reported to result in dysfunctional migration or reduced contractile function [14–16]. In vivo CaD knockout and mutant mouse homozygotes die perinatally, whereas a homozygous loss restricted to h-CaD is not lethal [15, 17–19]. Further, a xenopus morpholino l-CaD knockdown model shows reduced neural crest migration [20]. Additionally, vascular and heart developmental defects are present in a zebrafish morpholino CaD knockdown model; however, similar defects are not reported in other known in vivo models [15, 17–22].

Published data on the role of l-CaD in cancer are, in part, conflicting and suggest that *CALD1* acts both as a tumor suppressor or as an oncogene. Some preclinical studies suggest that l-CaD suppresses cancer cell migration in vitro [23–28]. However, conversely, in vitro data from both older and more recent studies also suggest that l-CaD promotes migration and invasion of cancer cells [29–33]. Studies based on patient samples suggest that higher l-CaD expression associates with worse prognosis (oral squamous cell carcinoma, gastric cancer, and bladder cancer), higher histopathological grade (glioblastoma and bladder cancer), increased immune infiltrates (gastric cancer and bladder cancer), and metastases (oral squamous cell carcinoma) [31, 32, 34–36]. Additionally, upregulated l-CaD expression associates with therapy resistance to the estrogen receptor modulator tamoxifen in breast cancer and with resistance to chemoradiotherapy in rectal cancer [37, 38]. The regulation of l-CaD expression in cancer is likely yet to be fully uncovered, but several factors, including p53, GR, and Cdk5, are implicated to have a role in specific contexts [24, 30, 39–46].

Here, we have characterized the role of l-CaD in PCa by analyzing co-expression data from the largest PCa patient data sets and experimentally by using monolayer- and 3D-cultured PCa cells, zebrafish PCa xenograft models, and castration-resistant VCaP xenograft mouse models. We show that l-CaD associates with EMT, GR-mediated antiandrogen resistance, and the formation of metastases in PCa. We conclude that l-CaD is critical in forming metastases in PCa and is upregulated in PCa cells that acquire therapy resistance by GR upregulation.

## Results

### l-CaD is expressed in PCa cell lines and is strongly downregulated during steroid hormone deprivation

**Fig. 1:**
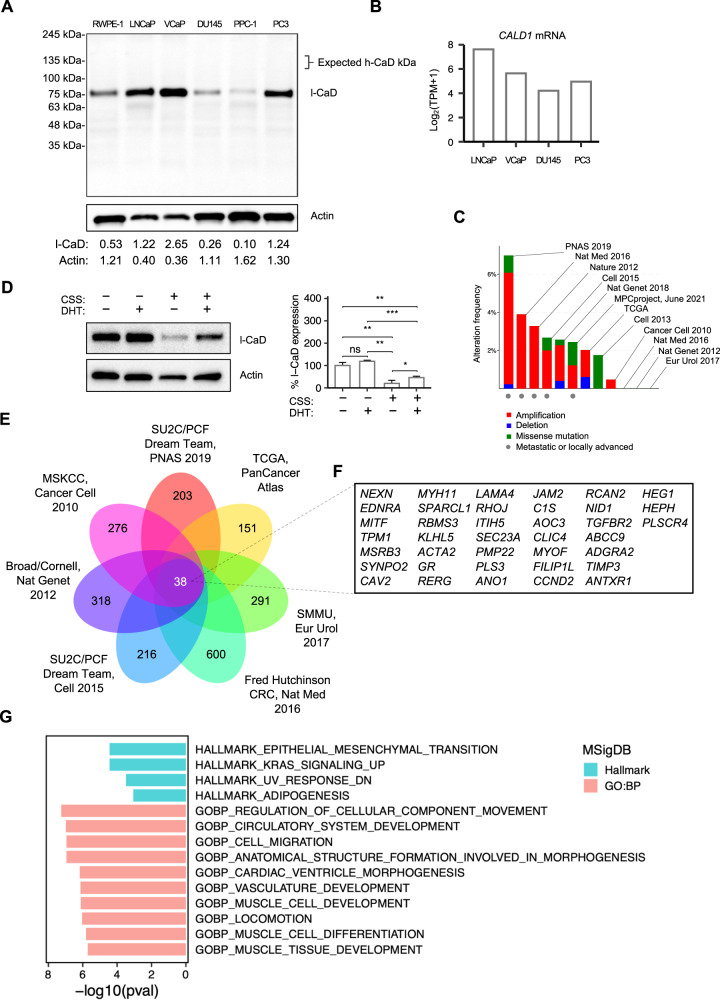
l-CaD is expressed in PCa cell lines, and *CALD1* co-expresses with positive regulators of EMT in PCa patient data sets. **A** Western blot depicting l-CaD protein expression in commercial PCa cell lines. **B** Barplots representing *CALD1* mRNA expression data in PCa cell lines acquired from Cancer Cell Line Encyclopedia. **C** Frequency of *CALD1* alterations in publicly available PCa patient data sets containing *CALD1* mutation and copy number alteration data [67–78]. **D** Representative Western blot depicting l-CaD expression and barplot depicting pooled densitometry of Western blot bands from three biological repeats after 72 h of 5% CSS culture and 24 h DHT treatment. **E** Venn diagram showing the concordance of top 1000 mRNAs co-expressed with *CALD1* between PCa patient data sets available on cBioPortal. **F** List of concordant hits of mRNA co-expression with *CALD1*. **G** MSigDB (version 7.5) hallmark gene sets that significantly overlap and ten MSigDB GO:BP gene sets with the highest percentage of overlap with the list of mRNA concordantly co-expressed with *CALD1*.

To examine which CaD isoforms were expressed in PCa, we performed a Western blot to analyze the protein expression from a variety of commercial PCa cell lines cultured in recommended growth conditions (Fig. 1A). All the tested cell lines solely expressed the non-muscle isoform l-CaD (70–80 kDa). No bands were detected in the 120–150 kDa range where the smooth muscle-associated isoform h-CaD migrates. The protein expression levels detected were in line with mRNA data from the Cancer Cell Line Encyclopedia (Fig. 1B). Notably, no differences in expression between androgen-sensitive and androgen-independent cell lines were observed.

Next, we examined published PCa patient data sets with both mutation and copy number alteration data available to study the frequency of alterations in the *CALD1* gene (Fig. 1C). Overall, *CALD1* alterations in tumors were not particularly common, but interestingly, amplifications were the most common alteration observed, and deletion was the least frequent type of alteration. The data sets with the highest frequency of amplifications were data sets of metastatic or locally advanced tumors. Comparing survival between altered and unaltered patients in the metastatic data sets, we observed a significantly decreased overall survival in a data set of 48 cases of metastatic and high-grade localized PCa, while differences in other data sets were not significant (Supplementary Fig. S1A, B).

To study whether l-CaD was regulated by AR signaling—the major driver of PCa progression—we looked at l-CaD expression in androgen-sensitive VCaP cells in conditions altering the AR activation status. We observed a substantial reduction of l-CaD expression in VCaP cells cultured in charcoal-stripped serum (CSS) media, while the expression levels were increased upon the addition of dihydrotestosterone (DHT) to the cells (Fig. 1D). Next, we wanted to study whether l-CaD associated with AR expression in PCa but found no correlation between *AR* and *CALD1* mRNA expression by examining the published PCa patient data sets, including TCGA (Supplementary Fig. S1C). Taken together, these data suggested that l-CaD expressed in PCa cells strongly responded to steroid hormone deprivation in androgen-sensitive cell lines, but the expression was also retained even in AR-negative cells, and *CALD1* did not correlate with *AR* expression in PCa.

### *CALD1* expression in PCa is associated with the expression of positive regulators of EMT and known markers of the mesenchymal phenotype

To assess in an unbiased manner which transcripts were associated with *CALD1* in PCa, we performed an integrative analysis of co-expression data from seven PCa data sets (Fig. 1E). We found 38 transcripts that were among the top 1000 positively co-expressed mRNAs in all data sets (Fig. 1F). To this list of 38 transcripts, we performed Molecular Signatures Database (MSigDB) analysis comparing the list with hallmark gene sets and gene ontology gene sets of biological processes (Fig. 1G). Interestingly, the identified list shared transcripts with EMT, cell migration, and locomotion gene sets. The identified EMT-associated transcripts were either involved in the positive regulation of EMT or commonly expressed in the mesenchymal phenotype. Other interrelated gene sets were circulatory system development and vasculature development, suggesting *CALD1* may also be involved in angiogenesis. Identified transcripts were also associated with three muscle-related gene sets.

We further explored *CALD1* co-expression with regulators of cancer hallmarks, actin-related processes, and relevant signaling pathways based on curated sets of positive and negative regulators separately (Gene Ontology: Biological Processes) on mRNA level in the largest single PCa patient data set (PCa subset of the PanCancer TCGA data set) by producing *CALD1* co-expression heatmaps. The percentages of positively and negatively co-expressed (determined in our analysis by cutoff at Spearman’s rank correlation coefficient ≥0.3 and ≤−0.3, respectively) regulators were evaluated (Supplementary Table 1). Positively co-expressed regulators were most common within the gene set of positive regulators of EMT (52.1% of regulators in the gene set) (Supplementary Fig. S2C and Supplementary Table 1). Other evaluated positive regulators of cancer hallmarks that showed high co-expression were regulators of angiogenesis (38.1%) and proliferation (32.6%) (Supplementary Fig. S2A, S2B, and Supplementary Table 1). Co-expression appeared to be, to some extent, present in both negative and positive regulators in analyzed processes, with the exception of anoikis, which was interestingly observed to have an almost exclusively negative association with *CALD1* (Supplementary Fig. S2F and Supplementary Table 1). To further investigate the association between *CALD1* and EMT using co-expression data in an alternative approach, we generated a custom gene set of epithelial and mesenchymal markers in which we observed predominantly positive co-expression between *CALD1* and the mesenchymal markers (Supplementary Fig. S2D).

### l-CaD enhances growth in organotypic culture while knockdown of l-CaD does not change proliferation in 2D culture

**Fig. 2:**
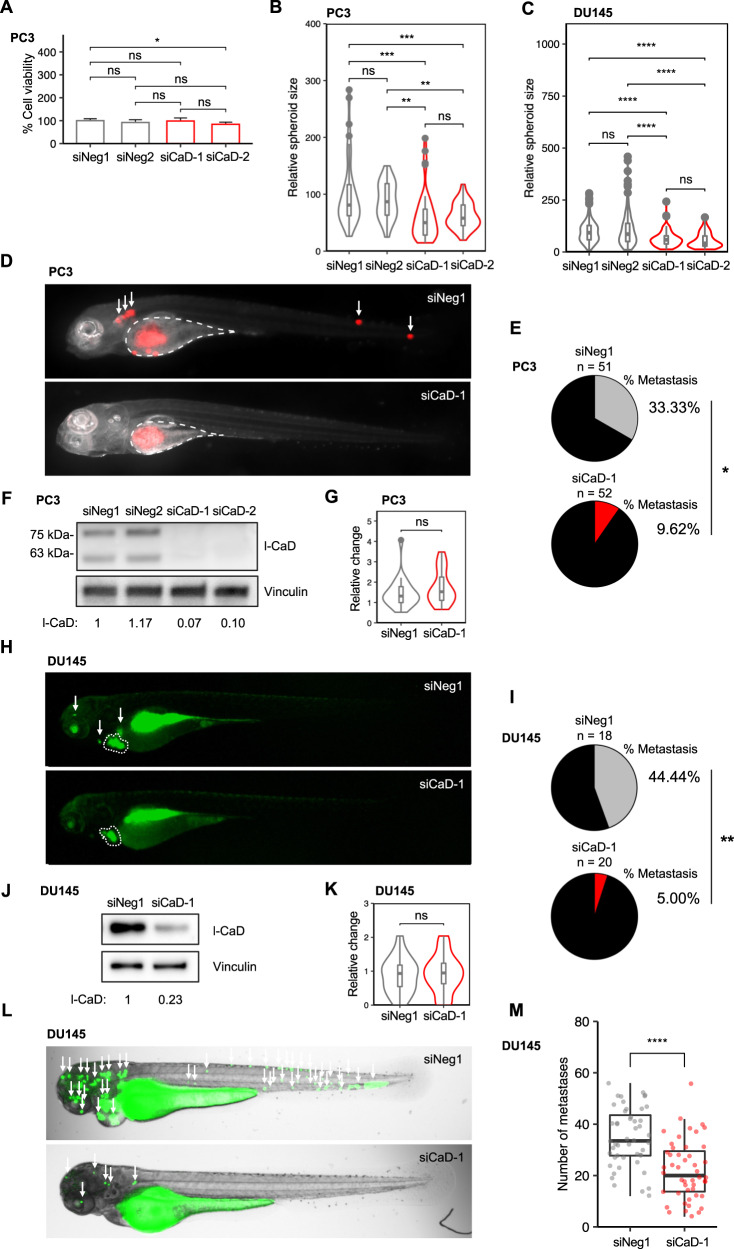
Loss of l-CaD impairs metastasis and invasion of PCa cells. **A** Barplot representing viability in PC3 cells transfected with siNeg1, siNeg2, siCaD-1, or siCaD-2. The mean and standard deviation (SD) of three experiments are shown (ns = not significant and *p < 0.05 as determined by the Mann–Whitney–Wilcoxon two-sided test). **B** Violin plot of relative spheroid size in PC3 cells (*n* = 160) transfected with siNeg1, siNeg2, siCaD-1, or siCaD-2 and grown in 3D basement membrane matrix culture. Boxplot indicates median and whiskers indicate 1.5 times interquartile range pooled from three experiments (ns = not significant, **p < 0.01, and ***p < 0.001 as determined by *t*-test). **C** Violin plot of relative spheroid size in DU145 cells (*n* = 527) transfected with siNeg1, siNeg2, siCaD-1, or siCaD-2 and grown in 3D basement membrane matrix culture. Boxplot indicates median and whiskers indicate 1.5 times interquartile range pooled from three experiments (ns = not significant, **p < 0.01, and ***p < 0.001 as determined by *t*-test). **D** Representative fluorescent merge images of zebrafish embryos 4 days after yolk sac microinjection of mCherry PC3 cells transfected with siNeg1 or siCaD-1. The injection site is highlighted in white. Metastases are indicated with arrows. **E** Pie chart showing the percentage of zebrafish embryos with visible metastases 4 days after injection pooled from three experiments (*p < 0.05 as analyzed by Fisher’s exact test). **F** Western blot depicting expression levels of l-CaD in mCherry PC3 cells transfected with siNeg1, siNeg2, siCaD-1, or siCaD-2 48 h after transfection. **G** Representative violin plots of relative change in primary tumor size (size 4 days after injection / size 1 day after injection). The tumor area was measured from fluorescent images of zebrafish. Boxplot indicates median, and whiskers indicate 1.5 times interquartile range (ns = not significant as determined by *t*-test). **H** Representative fluorescent images of zebrafish embryos 4 days after pericardial microinjection of CellTracker Green labeled DU145 cells transfected with siNeg1 or siCaD-1. The primary tumor is highlighted in white. Metastases are indicated with arrows. **I** Pie chart showing the percentage of zebrafish embryos with visible metastases 4 days after injection to the pericardial cavity (**p < 0.01 as analyzed by Fisher’s exact test). **J** Western blot depicting expression levels of l-CaD in DU145 cells transfected with siNeg1 or siCaD-1 48 h after transfection. **K** Violin plots of relative change in primary tumor size (size 4 days after injection/size 1 day after injection). The tumor area was measured from fluorescent images of zebrafish. Boxplot indicates median, and whiskers indicate 1.5 times interquartile range (ns = not significant as determined by *t*-test). **L** Representative brightfield and fluorescent merge images of zebrafish embryos 1 day after microinjection of CellTracker Green labeled DU145 cells transfected with siNeg1 or siCaD-1 to the common cardinal vein. Metastases are indicated with arrows. **M** Box and jitter plots depicting the number of metastases in zebrafish embryos (*n* = 96). Boxplot indicates median, and whiskers indicate 1.5 times interquartile range (****p < 0.0001 as determined by *t*-test).

Next, we experimentally investigated in vitro whether l-CaD played a role in regulating viability corresponding to our in silico analysis suggesting a positive correlation with proliferation. We did not observe any significant change in PC3 cell viability after l-CaD knockdown with siRNAs as measured by an MTS assay (Fig. 2A). In accordance, no significant effect on viability was found either after l-CaD knockdown in androgen-sensitive VCaP cells (Supplementary Fig. S3A). We then continued to study the role of l-CaD in the regulation of PCa growth using an organotypic basement membrane matrix culture, and the results showed that the knockdown of l-CaD reduced growth in 3D cultures (Fig. 2B, C, and Supplementary Fig. S3B). These data suggested that l-CaD was beneficial for the PCa cells in organotypic growth in 3D, although downregulation did not significantly affect viability in monolayer culture.

### Knockdown of l-CaD impairs the formation of metastases in PCa xenograft zebrafish

Our co-expression analysis (Fig. 1G–I) suggested that *CALD1* was co-expressed with transcripts involved in the regulation of EMT and associated with cell migration and locomotion. To study the role of l-CaD in cancer cell migration and invasion in vivo, we injected mCherry PC3 cells transfected with siRNAs targeting l-CaD or non-targeting siRNAs into zebrafish larvae yolk sacs and assessed the formation of metastases after four days (Fig. 2D, E). The zebrafish assays were performed in parallel with viability assessments by monolayer culture MTS and with organotypic 3D culture growth area analysis, as well as with the knockdown verification at protein level by Western blotting (Fig. 2F). We observed that the rate of metastases in the xenografted zebrafish was reduced to less than one-third upon siRNA knockdown of *CALD1* (Fig. 2E). The size of the primary tumor was not changed after l-CaD knockdown, suggesting a specific effect pertaining to the metastasis process (Fig. 2G). As with non-mCherry PC3 cells, viability was not affected by l-CaD knockdown in mCherry PC3 cells (Supplementary Fig. S3C). While remaining viable, and producing spheroids in basement membrane matrix, the mCherry PC3 cells exhibited reduced growth after l-CaD knockdown similar to non-mCherry PC3 cells (Supplementary Fig. S3D, E). Similar results were observed in two DU145 cell xenograft models, wherein l-CaD knockdown led to a lower rate of metastasis and a reduced number of metastases after the cells were microinjected into pericardial cavity or common cardinal vein (Fig. 2H–M). These data suggested that when l-CaD expression was downregulated, aggressive PCa cells exhibited decreased capability to metastasize while remaining otherwise viable, indicating that l-CaD was specifically involved in metastasis and invasion.

### l-CaD is upregulated upon GR activation in PCa in vitro

**Fig. 3:**
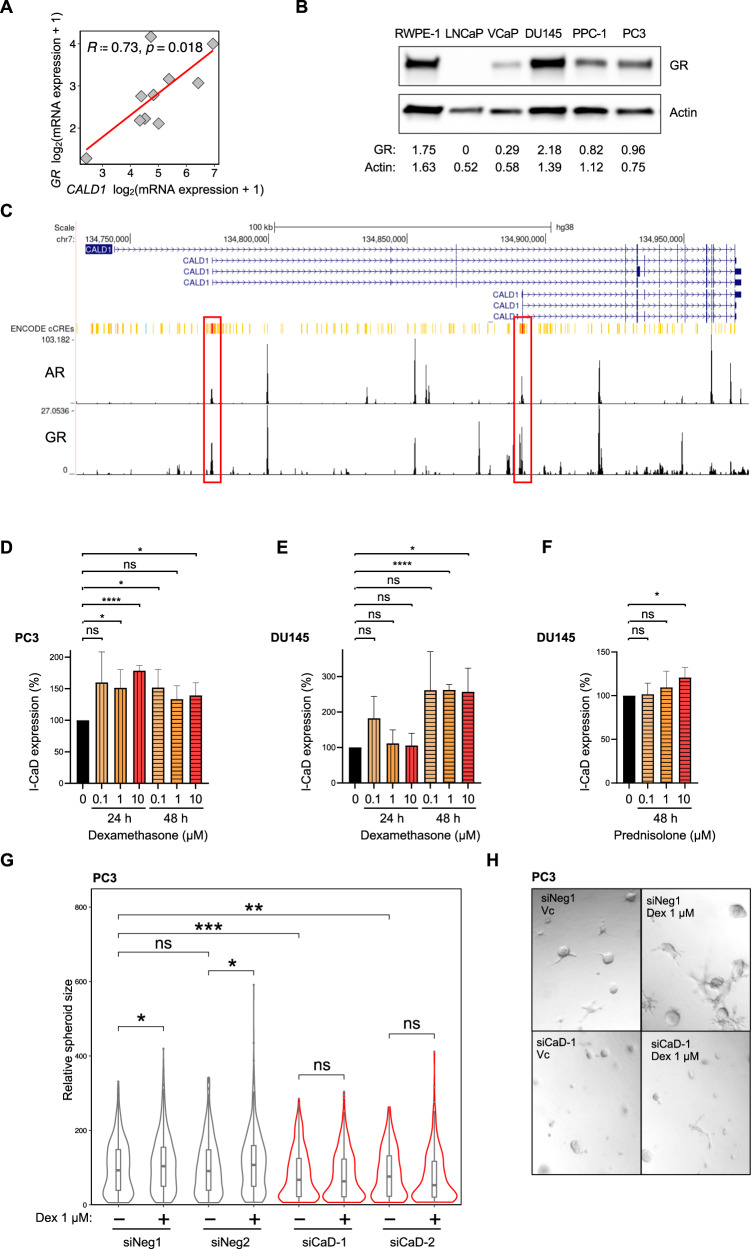
l-CaD expression is upregulated by GR activation promoting growth in organotypic cell culture. **A** Correlation between *GR* and *CALD1* mRNA in patient-derived organoids (cBioPortal) [79]. **B** Western blot depicting GR expression in commercial PCa cell lines. **C** UCSC genome browser visualization of ReMap ChIP-seq tracks for AR and GR in *CALD1* with ENCODE candidate promoter-like signatures highlighted in red and enhancer-like signatures highlighted in yellow and orange. **D** Barplots depicting l-CaD protein expression in PC3 cells treated with Dex (0.1 µM, 1 µM, or 10 µM) after 24 or 48 hours. The mean and SD of three experiments are shown (ns = not significant, *p < 0.05, and ****p < 0.0001 as determined by *t*-test). **E** Barplots depicting l-CaD protein expression in DU145 cells treated with Dex (0.1 µM, 1 µM, or 10 µM) after 24 or 48 hours. The mean and SD of three experiments are shown (ns = not significant, *p < 0.05, and ****p < 0.0001 as determined by *t*-test). **F** Barplots depicting l-CaD protein expression in DU145 cells treated with prednisolone (0.1 µM, 1 µM, or 10 µM) after 48 hours. The mean and SD of three experiments are shown (ns = not significant and *p < 0.05 as determined by *t*-test). **G** Violin plots of relative spheroid size in PC3 cells transfected with siNeg1 or siCaD-1 and grown in the presence or absence of 1 µM Dex in basement membrane matrix. Boxplot indicates median and whiskers indicate 1.5 times interquartile range pooled form three experiments (ns = not significant, *p < 0.05, and **p < 0.01 as determined by *t*-test). **H** Representative brightfield image of day 5 organotypic culture of PC3 cells treated with siNeg-1 or siCaD-1 and exposed to 1 µM Dex or vehicle (DMSO) on day 3 of culture in basement membrane matrix.

After establishing the anti-tumorigenic effect of l-CaD knockdown, we extended our analysis of patient samples to investigate possible regulators of l-CaD in PCa. Among the concordant hits of co-expression with *CALD1* between seven patient data sets (Fig. 1F), there were two transcription factors, GR (*NR3C1*) and melanocyte inducing transcription factor (*MITF*), that could directly regulate l-CaD expression. Due to the known connection of GR to PCa, we focused further on characterizing the GR–CaD interplay in PCa. To confirm that the significant co-expression was not caused by stromal h-CaD mRNA in the patient samples, we analyzed a published data set of patient-derived organoids lacking stroma and found a strong correlation between *CALD1* and *GR* expression (Fig. 3A).

We observed that GR was expressed in commercial PCa cell lines except for LNCaP cells which also have a CaD mutation (Fig. 3B). We performed zebrafish xenograft assay also with LNCaP cells microinjecting them into common cardinal vein and found that l-CaD knockdown reduced the number of metastases despite LNCaP cells being AR-positive and lacking GR expression (Supplementary Fig. S4A–C). To further understand the role of AR and GR in the regulation of l-CaD, we examined ReMap ChIP-seq tracks for AR and GR in the UCSC genome browser, and found that both receptors displayed peaks in the putative promoter and enhancer sites at *CALD1* (Fig. 3C). Next, we investigated the effect of GR activation on the expression of l-CaD in PCa cells. We observed that GR-expressing PC3 cells responded to a GR activator dexamethasone (Dex) with l-CaD upregulation at 24 or 48 hours (Fig. 3B, D). Similarly, in DU145 cells, treatment with Dex and prednisolone resulted in the upregulation of l-CaD expression after 48 hours of treatment (Fig. 3E, F). Dex stimulation improved spheroid growth of PC3 cells in basement membrane matrix cultures unless the l-CaD expression was knocked down using siRNA prior to the Dex treatment (Fig. 3G, H). These data suggested that l-CaD was upregulated by GR activation in AR-negative PCa.

### Knockdown of l-CaD shifts the phenotype away from the mesenchymal cell state in PCa in organotypic culture

**Fig. 4:**
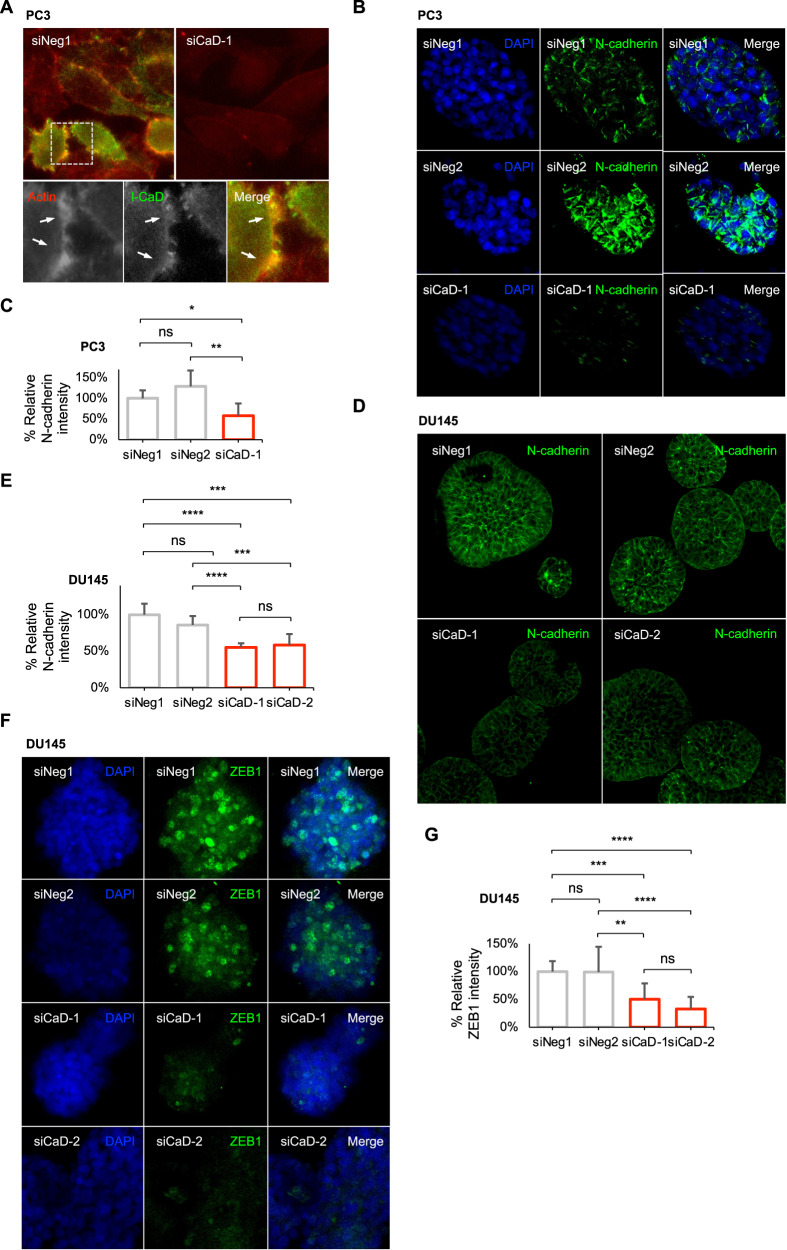
l-CaD colocalizes with actin in filopodia, and the knockdown of l-CaD downregulates N-cadherin. **A** Representative fluorescent merge images of PC3 cells transfected with siNeg1 or siCaD-1 and stained with antibodies recognizing actin and l-CaD. The lower row shows separate images of actin, l-CaD, and merge in a close-up of the upper siNeg1 image. **B** Representative fluorescent images of PC3 spheroids transfected with siNeg1, siNeg2, or siCaD-1, stained with N-cadherin, and counterstained with DAPI. **C** Barplots depicting N-cadherin protein intensity in spheroids formed by transfected PC3 cells shown in **B**. Average was calculated from multiple individual spheroids (*N* = 22) measured for mean intensity. The mean and SD are shown (ns = not significant, *p < 0.05, and **p < 0.01 as determined by *t*-test). **D** Representative fluorescent images of DU145 spheroids transfected with siNeg1, siNeg2, siCaD-1, or siCaD-2 stained against N-cadherin. **E** Barplots depicting N-cadherin protein intensity in spheroids formed by transfected DU145 cells shown in **D**. Average was calculated from multiple individual spheroids (*N* = 30) measured for mean intensity. The mean and SD shown (ns = not significant, ***p < 0.001, and ****p < 0.0001 as determined by *t*-test). **F** Representative fluorescent images of DU145 spheroids transfected with siNeg1, siNeg2, siCaD-1, or siCaD-2 stained against ZEB1. **G** Barplots depicting ZEB1 protein intensity in spheroids formed by transfected DU145 cells shown in **F**. Average was calculated from multiple individual spheroids (*N* = 58) measured for mean intensity. The mean and SD shown (ns = not significant, **p < 0.001, ***p < 0.001, and ****p < 0.0001 as determined by *t*-test).

After associating *CALD1* with the expression of positive EMT regulators and mesenchymal markers in the patient data (Fig. 1), we wanted to see if these same markers could be observed in the models used to demonstrate that l-CaD silencing leads to diminished invasiveness. l-CaD colocalized with actin in PC3 cell filopodia (Fig. 4A). Staining PC3 cell spheroids cultured in basement membrane matrix showed downregulated expression of the mesenchymal cell state-associated N-cadherin after l-CaD knockdown (Fig. 4B, C), whereas no difference in the expression of E-cadherin was observed upon l-CaD knockdown (Supplementary Fig. S5A, B). Furthermore, DU145 cell spheroids were similarly observed having downregulated N-cadherin and ZEB1 expression after l-CaD knockdown (Fig. 4D–G). By staining the zebrafish used in the in vivo xenograft model studying metastases, we confirmed the lack of l-CaD and reduced N-cadherin expression in the PC3 mCherry cells after l-CaD knockdown with siRNA (Supplementary Fig. S5C). These data taken together with the patient co-expression analyses, provided evidence supporting the role of l-CaD in promoting EMT in PCa.

### GR upregulation in enzalutamide resistance leads to upregulated l-CaD expression in vivo

**Fig. 5:**
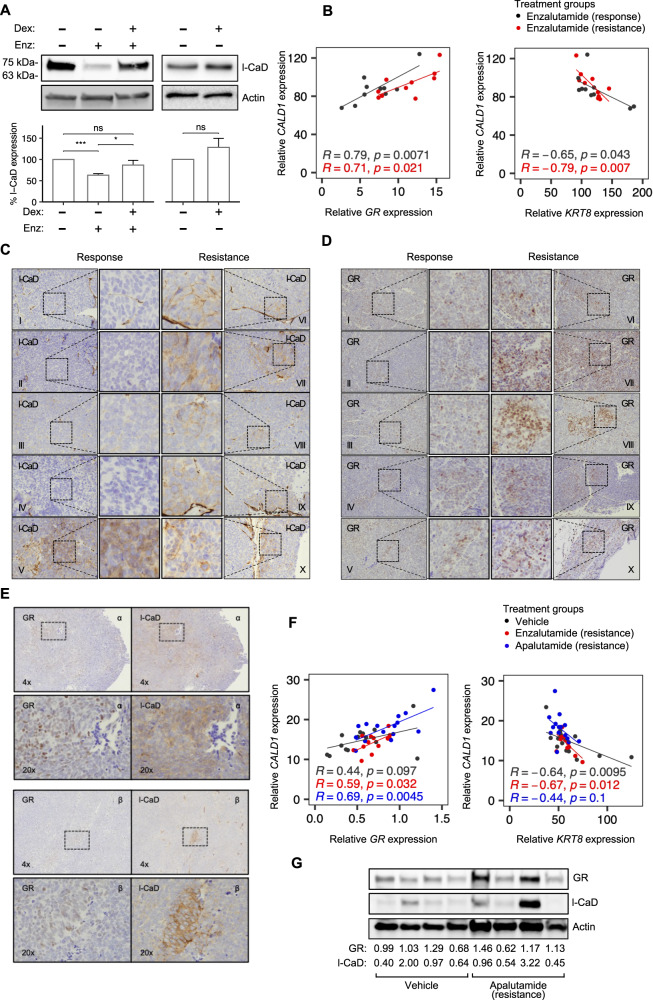
l-CaD expression is upregulated during enzalutamide resistance in vivo. **A** Representative Western blot depicting l-CaD expression and barplot depicting pooled densitometry of Western blot bands from three biological repeats after treatment with enzalutamide (5 days, 10 µM) and Dex (24 h, 0.1 µM). **B** Scatterplots depicting Pearson correlation between *CALD1* and *GR* or *KRT8* mRNA from enzalutamide-treated VCaP xenograft mice. **C** IHC of l-CaD in castration-resistant VCaP xenografts during enzalutamide response (left) and after attained enzalutamide resistance (right). Individual mice are denoted using Roman numerals. **D** IHC of GR in castration-resistant VCaP xenografts during enzalutamide response (left) and after attained enzalutamide resistance (right). Individual mice are denoted using Roman numerals. **E** IHC of l-CaD and GR from adjacent slices of VCaP xenograft tumors in mice during apalutamide resistance. The lower row shows a higher magnification image of the area highlighted on the upper row. Individual mice are denoted using Greek letters. **F** Scatterplots depicting Pearson correlation between *CALD1* and *GR* or *KRT8* mRNA from vehicle-, enzalutamide-, and apalutamide-treated VCaP xenograft mice. **G** Western blot of vehicle- and apalutamide-treated castration-resistant VCaP xenografts. Quantification of the GR and l-CaD signal relative to actin loading control signal and the average vehicle-treated signal is shown below.

To study l-CaD during the development of resistance to AR-targeting therapies, we used androgen-sensitive PCa cells. To overcome interference to GR signaling by AR activation, we used a lead-in treatment with an antiandrogen (enzalutamide) in VCaP cells and observed that l-CaD expression was upregulated by Dex, while also slightly increasing in response to Dex in the absence of prior antiandrogen (Fig. 5A). As an in vivo model of castration resistance, we re-analyzed subcutaneous VCaP xenograft tumors grown in nude mice [47, 48]. Mice with castration-resistant tumors were treated with an antiandrogen (enzalutamide) and sacrificed either when tumors were still responding to antiandrogen or after the eventual appearance of resistance to antiandrogen. We re-analyzed RNA-Seq data of the subcutaneous tumors and observed *GR* having a positive correlation with *CALD1* (Fig. 5B). To examine l-CaD and GR at protein levels and to distinguish between stromal and tumor CaD, we performed IHC on the xenograft tumors. We observed the appearance of islands of cells with upregulated l-CaD expression by IHC in resistant tumors when compared with tumors from mice sacrificed during antiandrogen response (Fig. 5C). Notably, one mouse in the response group with exceptionally high PSA (9.462 ng ml^−1^ vs. an average of 2.037 ng ml^−1^ in the rest of the response group), which likely reflects incomplete response, was observed to have areas of l-CaD expression similar to that of the resistant group. Staining of the samples with an antibody recognizing GR showed a similar pattern of spotted upregulation in the resistant samples, whereas a lower baseline expression was observed in the response group (Fig. 5D). We also stained adjacent slides of an orthotopic VCaP xenograft mouse model [49] and observed similar islands of l-CaD upregulation in tumors treated with the antiandrogen (apalutamide) within the same regions that presented GR upregulation (Fig. 5E). Moreover, we re-analyzed previously generated RNA-seq data of reported orthotopic xenografts and found similar positive correlation between *GR* and *CALD1* in the resistant tumors (Fig. 5F). Western blot analysis of tumor homogenates from the orthotopic VCaP xenografts also showed a trend of increase in both GR and l-CaD expression in the tumors treated with the antiandrogen (apalutamide), although the expressions were heterogeneous as expected based on our IHC analyses (Fig. 5G). Thus, l-CaD upregulation was associated in vivo with areas of GR upregulation after resistance to antiandrogens.

### *GR* expression correlates with *CALD1* expression, while *KRT8* expression is negatively correlated with *CALD1* in vivo

After establishing the connection between GR and l-CaD in the VCaP xenograft mouse models, we used RNA-Seq analysis to study the association with EMT-related transcripts. We observed *CALD1* having a negative correlation with *KRT8* in both the subcutaneous and the orthotopic xenografts (Fig. 5B, F). In line with our in silico analyses from patient data (Fig. 1) and our analysis of immunofluorescence in PC3 cell spheroids (Supplementary Fig. S5A, B), *CALD1* and *CDH1* (encodes E-cadherin) expressions were non-correlating in the subcutaneous xenografts (Supplementary Fig. S6A). Fitting the VCaP model being non-metastatic, markers for mesenchymal phenotype were commonly undetectable or lowly expressed in VCaP xenografts (Supplementary Table 2). None of the correlation analyses with *CALD1* and mesenchymal markers reached statistical significance in the subcutaneous xenografts, which again is likely due to the VCaP model being non-metastatic (Supplementary Fig. S6A). However, some positive EMT-related correlations in the orthotopic model were significant (*SNAI1* in the apalutamide resistance and *TWIST2* in the enzalutamide resistance), despite the VCaP model being non-metastatic (Supplementary Fig. S6B). Using IHC staining of vimentin in the subcutaneous tumors we observed no expression in the samples of the resistant group (Supplementary Fig. S6C). In conclusion, using the VCaP xenograft mouse models, we were able to verify our initial observations from PCa patient data sets showing the association between *CALD1* and *KRT8* and *GR*.

## Discussion

Despite the initial response to the therapeutic targeting of AR signaling, aggressive PCa eventually develops castration resistance and progresses to lethal metastatic PCa. Here, we have characterized one critical mechanism involved in antiandrogen therapy-resistant PCa. We report that l-CaD is expressed in PCa cell lines and that *CALD1* amplifications are more common in metastatic or locally advanced than primary tumor PCa patient data sets. In a co-expression analysis of the largest PCa patient data sets, we find *CALD1* to correlate with EMT markers and *GR*. The knockdown of l-CaD in vitro limits organotypic growth and downregulates EMT marker expression but has no effect on monolayer growth. Finally, we experimentally show in vivo that l-CaD induces EMT, promotes metastasis, and is upregulated in antiandrogen-resistant PCa together with GR.

Several previous studies have described an upregulation of GR as one important mechanism in antiandrogen-resistant PCa [50–55]. We show that l-CaD is upregulated by GR activation in AR-negative PCa cell lines and after AR inhibition in AR-positive cells. Moreover, *CALD1* expression correlates with *GR* expression in PCa patient samples and patient-derived organoids, and by IHC, we show that l-CaD and GR are upregulated in antiandrogen-resistant VCaP xenograft mouse models. The detailed mechanism for GR-induced upregulation in PCa is not known, but interestingly previous work shows that activated GR binds to *CALD1* promoter in lung cancer cells, providing a mechanism for l-CaD upregulation by GR [24]. In accordance, our analysis of the published ChIP-seq data available on the UCSC genome browser also support that GR may directly contribute to l-CaD expression regulation. With a myriad of identified regulators for CaD, modulators other than GR could also play a part in pushing l-CaD expression in PCa [30, 39–46]. Additionally, calmodulin binding and the regulation of l-CaD phosphorylation by CDK1, Erk, p38 MAPK, isoforms of s100, and p21-activated kinases (PAK) 1 and 2 may further enhance l-CaD effects in PCa [29, 40, 56–59].

In our analyses, l-CaD shows no association with the common PCa driver AR, but, interestingly, a few *CALD1* amplifications were already present in primary tumor patients, adding to the possibility of l-CaD having a role in PCa progression outside antiandrogen-resistance via AR bypassing by GR upregulation. GR signaling in PCa is also a complex entity as glucocorticoids act as partial antiandrogens in the presence of androgens, lower steroid levels by inducing balancing feedback in the pituitary, and effectively alleviate the side effects of utilized therapies. The presence of AR seems to complicate the acute response to GR ligands which may, in part, be explained by AR signaling regulating GR negatively [53]. Our experiments with VCaP cells show that l-CaD is upregulated by GR activation when stimulation is preceded by antiandrogen treatment. Thus, the GR-dependent upregulation of l-CaD in AR-expressing cells seems to be enhanced by AR inhibition. Our analysis of the published ChIP-seq data suggest that, in addition to GR, also AR may contribute to l-CaD regulation. Furhermore, it is probable that the regulation is also modulated variously during different phases of PCa progression and if direct, it is likely further influenced by co-factor binding. Taken together, our results display the complexity of the interconnected AR and GR signaling in therapy resistant PCa and suggest that GR activation upregulates l-CaD in the absence of active AR signaling in PCa.

Using PCa cells injected into zebrafish larvae as a model, we now show that l-CaD expression is critical for PCa metastasis in vivo. Silencing l-CaD reduces the rate of metastases in the PC3 xenograft zebrafish by two-thirds and leads to an even more pronounced reduction in the rate of metastasis in the DU145 xenograft zebrafish. Importantly, viability is unaffected by the silencing of l-CaD, supporting a specific role in metastasis. Notably, our results in the zebrafish metastasis assay with AR-positive and GR-negative LNCaP cells, suggest a more general role for l-CaD in promoting metastasis in PCa cells independently of AR or GR status. Interestingly, the silencing of l-CaD also reduces spheroid size when cells are grown in basement membrane matrix. The discrepancy between the results observed in plastic 2D and 3D basement membrane matrix gel is possibly attributable to the latter model not only measuring proliferation capacity but more closely resembling in vivo environment and also characteristics involved in metastasis. Moreover, 3D culture may also represent the microenvironment more accurately when compared to the traditional invasion and migration assays. Previous preclinical studies in vitro show an increase in migration and invasion after l-CaD knockdown in lung cancer, gastric cancer, breast cancer, hepatocellular carcinoma, and PCa cells in contrast to our results [24–28]. However, supporting our findings, a positive correlation of l-CaD expression with in vitro migration and invasion is reported in myoblasts, HeLa, bladder cancer, and osteosarcoma cells [29–31, 33]. Similarly, previous reports on the role of GR in migration and invasion in vitro are contrasting [60, 61]. Previous work on the role of GR signaling in radial migration of neurons interestingly suggests that an appropriate l-CaD level is critical for optimal migration in vivo [14]. In conclusion, by providing for the first time in vivo data of metastatic zebrafish xenografts, our results add to the evidence supporting that l-CaD promotes invasion and migration and, further, the formation of metastases.

Cancer cell plasticity is a requirement for the formation of metastases, which cells can obtain to different extents by undergoing EMT [62]. VCaP xenograft mouse models we use are not metastatic, which is reflected in the low expression of mesenchymal markers in the tumor data. However, we show that epithelial marker expression is negatively correlated with *CALD1* in the xenografts, and two mesenchymal marker-expressing PCa cell lines have reduced N-cadherin expression after silencing l-CaD in 3D culture. Additionally, we show high co-expression with several EMT regulators and mesenchymal markers and *CALD1* in PCa TCGA and combined analysis of six other PCa patient data sets. Notably, the common epithelial marker E-cadherin does not correlate with l-CaD in the patient data sets and does not show differential expression upon silencing l-CaD in 3D culture. Retained E-cadherin expression may suggest that l-CaD induces partial EMT, which is interestingly associated with calmodulin, a known interactor of l-CaD [62, 63].

It is important to acknowledge limitations associated with specific cell lines used to study PCa. While PC3 and DU145 cells are among the most widely utilized PCa cell lines alongside LNCaP cells, they do not express significant levels of AR protein in contrast to LNCaP and VCaP cells [64]. Given that the majority of PCa express AR, it is important to avoid generalizing the findings obtained with AR-negative cell lines. Instead, PC3 and DU145 cells may be considered representative of AR low or negative PCa subtypes, which, including neuroendocrine PCa, may make up one-fifth of castration-resistant PCa cases [65, 66].

Our results describe a new mechanism that promotes EMT and metastasis in GR-upregulated antiandrogen-resistant PCa. In summary, our data suggest that antiandrogen resistance may give rise to colonies of GR-upregulated PCa cells within the tumor where l-CaD expression is induced in response to GR activation in the absence of AR signaling. The induction of l-CaD promotes EMT in the PCa cells and may eventually stimulate the dissemination of the colony. In conclusion, our study demonstrates that l-CaD is involved in facilitating metastasis in PCa. Herein described, l-CaD upregulation is potentially one specific target to prevent metastases in therapy resistant PCa.

## Materials and methods

Available in Supplementary information.

### Supplementary information

Supplementary Information

Supplementary Figures

Supplementary Tables

Original Data File

## Data Availability

Data are available upon reasonable request.

## References

[1] Sung H, Ferlay J, Siegel RL, Laversanne M, Soerjomataram I, Jemal A (2021). Global Cancer Statistics 2020: GLOBOCAN Estimates of Incidence and Mortality Worldwide for 36 Cancers in 185 Countries. CA Cancer J Clin.

[2] Watson PA, Arora VK, Sawyers CL (2015). Emerging mechanisms of resistance to androgen receptor inhibitors in prostate cancer. Nat Rev Cancer.

[3] Yamaguchi H, Condeelis J (2007). Regulation of the actin cytoskeleton in cancer cell migration and invasion. Biochim Biophys Acta.

[4] Sobue K, Muramoto Y, Fujita M, Kakiuchi S (1981). Purification of a calmodulin-binding protein from chicken gizzard that interacts with F-actin. Proc Natl Acad Sci USA.

[5] Sobue K, Tanaka T, Kanda K, Ashino N, Kakiuchi S (1985). Purification and characterization of caldesmon77: a calmodulin-binding protein that interacts with actin filaments from bovine adrenal medulla. Proc Natl Acad Sci USA.

[6] Hayashi K, Yano H, Hashida T, Takeuchi R, Takeda O, Asada K (1992). Genomic structure of the human caldesmon gene. Proc Natl Acad Sci USA.

[7] Payne AM, Yue P, Pritchard K, Marston SB (1995). Caldesmon mRNA splicing and isoform expression in mammalian smooth-muscle and non-muscle tissues. Biochem J.

[8] Watanabe K, Kusakabe T, Hoshi N, Saito A, Suzuki T (1999). h-Caldesmon in leiomyosarcoma and tumors with smooth muscle cell-like differentiation: its specific expression in the smooth muscle cell tumor. Hum Pathol.

[9] Walther S, Strittmatter F, Roosen A, Heinzer F, Rutz B, Stief CG (2012). Expression and alpha1-adrenoceptor regulation of caldesmon in human prostate smooth muscle. Urology..

[10] Hemric ME, Chalovich JM (1988). Effect of caldesmon on the ATPase activity and the binding of smooth and skeletal myosin subfragments to actin. J Biol Chem.

[11] Marston SB, Redwood CS (1992). Inhibition of actin-tropomyosin activation of myosin MgATPase activity by the smooth muscle regulatory protein caldesmon. J Biol Chem.

[12] Goncharova EA, Shirinsky VP, Shevelev AY, Marston SB, Vorotnikov AV (2001). Actomyosin cross-linking by caldesmon in non-muscle cells. FEBS Lett.

[13] Makuch R, Kulikova N, Graziewicz MA, Nowak E, Dabrowska R (1994). Polymerization of actin induced by actin-binding fragments of caldesmon. Biochim Biophys Acta.

[14] Fukumoto K, Morita T, Mayanagi T, Tanokashira D, Yoshida T, Sakai A (2009). Detrimental effects of glucocorticoids on neuronal migration during brain development. Mol Psychiatry.

[15] Pütz S, Barthel LS, Frohn M, Metzler D, Barham M, Pryymachuk G (2021). Caldesmon ablation in mice causes umbilical herniation and alters contractility of fetal urinary bladder smooth muscle. J Gen Physiol.

[16] Helfman DM, Levy ET, Berthier C, Shtutman M, Riveline D, Grosheva I (1999). Caldesmon inhibits nonmuscle cell contractility and interferes with the formation of focal adhesions. Mol Biol Cell.

[17] Guo H, Wang CL (2005). Specific disruption of smooth muscle caldesmon expression in mice. Biochem Biophys Res Commun.

[18] Guo H, Huang R, Semba S, Kordowska J, Huh YH, Khalina-Stackpole Y (2013). Ablation of smooth muscle caldesmon affects the relaxation kinetics of arterial muscle. Pflug Arch.

[19] Deng M, Boopathi E, Hypolite JA, Raabe T, Chang S, Zderic S (2013). Amino acid mutations in the caldesmon COOH-terminal functional domain increase force generation in bladder smooth muscle. Am J Physiol Ren Physiol.

[20] Nie S, Kee Y, Bronner-Fraser M (2011). Caldesmon regulates actin dynamics to influence cranial neural crest migration in Xenopus. Mol Biol Cell.

[21] Zheng PP, Severijnen LA, van der Weiden M, Willemsen R, Kros JM (2009). A crucial role of caldesmon in vascular development in vivo. Cardiovasc Res.

[22] Zheng PP, Severijnen LA, Willemsen R, Kros JM (2009). Caldesmon is essential for cardiac morphogenesis and function: in vivo study using a zebrafish model. Biochem Biophys Res Commun.

[23] Yoshio T, Morita T, Kimura Y, Tsujii M, Hayashi N, Sobue K (2007). Caldesmon suppresses cancer cell invasion by regulating podosome/invadopodium formation. FEBS Lett.

[24] Mayanagi T, Morita T, Hayashi K, Fukumoto K, Sobue K (2008). Glucocorticoid receptor-mediated expression of caldesmon regulates cell migration via the reorganization of the actin cytoskeleton. J Biol Chem.

[25] Hou Q, Tan HT, Lim KH, Lim TK, Khoo A, Tan IB (2013). Identification and functional validation of caldesmon as a potential gastric cancer metastasis-associated protein. J Proteome Res.

[26] Schwappacher R, Rangaswami H, Su-Yuo J, Hassad A, Spitler R, Casteel DE (2013). cGMP-dependent protein kinase Iβ regulates breast cancer cell migration and invasion via interaction with the actin/myosin-associated protein caldesmon. J Cell Sci.

[27] Dierks S, von Hardenberg S, Schmidt T, Bremmer F, Burfeind P, Kaulfuß S (2015). Leupaxin stimulates adhesion and migration of prostate cancer cells through modulation of the phosphorylation status of the actin-binding protein caldesmon. Oncotarget..

[28] Zhang J, Ren Z, Zheng D, Song Z, Lin J, Luo Y (2022). AHSA1 Promotes Proliferation and EMT by Regulating ERK/CALD1 Axis in Hepatocellular Carcinoma. Cancers.

[29] Manes T, Zheng DQ, Tognin S, Woodard AS, Marchisio PC, Languino LR (2003). Alpha(v)beta3 integrin expression up-regulates cdc2, which modulates cell migration. J Cell Biol.

[30] Jang SM, Kim JW, Kim D, Kim CH, An JH, Choi KH (2013). Sox4-mediated caldesmon expression facilitates differentiation of skeletal myoblasts. J Cell Sci.

[31] Lee MS, Lee J, Kim JH, Kim WT, Kim WJ, Ahn H (2015). Overexpression of caldesmon is associated with tumor progression in patients with primary non-muscle-invasive bladder cancer. Oncotarget.

[32] Li C, Yang F, Wang R, Li W, Maskey N, Zhang W (2021). CALD1 promotes the expression of PD-L1 in bladder cancer via the JAK/STAT signaling pathway. Ann Transl Med.

[33] Kokate SB, Ciuba K, Tran VD, Kumari R, Tojkander S, Engel U (2022). Caldesmon controls stress fiber force-balance through dynamic cross-linking of myosin II and actin-tropomyosin filaments. Nat Commun.

[34] Chang KP, Wang CL, Kao HK, Liang Y, Liu SC, Huang LL (2013). Overexpression of caldesmon is associated with lymph node metastasis and poorer prognosis in patients with oral cavity squamous cell carcinoma. Cancer.

[35] Cheng Q, Tang A, Wang Z, Fang N, Zhang Z, Zhang L (2021). CALD1 Modulates Gliomas Progression via Facilitating Tumor Angiogenesis. Cancers.

[36] Liu Y, Xie S, Zhu K, Guan X, Guo L, Lu R (2021). CALD1 is a prognostic biomarker and correlated with immune infiltrates in gastric cancers. Heliyon..

[37] Kim KH, Yeo SG, Kim WK, Kim DY, Yeo HY, Hong JP (2012). Up-regulated expression of l-caldesmon associated with malignancy of colorectal cancer. BMC Cancer.

[38] De Marchi T, Timmermans AM, Smid M, Look MP, Stingl C, Opdam M (2016). Annexin-A1 and caldesmon are associated with resistance to tamoxifen in estrogen receptor positive recurrent breast cancer. Oncotarget..

[39] Nishida W, Nakamura M, Mori S, Takahashi M, Ohkawa Y, Tadokoro S (2002). A triad of serum response factor and the GATA and NK families governs the transcription of smooth and cardiac muscle genes. J Biol Chem.

[40] Morita T, Mayanagi T, Yoshio T, Sobue K (2007). Changes in the balance between caldesmon regulated by p21-activated kinases and the Arp2/3 complex govern podosome formation. J Biol Chem.

[41] Mukhopadhyay UK, Eves R, Jia L, Mooney P, Mak AS (2009). p53 suppresses Src-induced podosome and rosette formation and cellular invasiveness through the upregulation of caldesmon. Mol Cell Biol.

[42] Quintavalle M, Elia L, Price JH, Heynen-Genel S, Courtneidge SA (2011). A cell-based high-content screening assay reveals activators and inhibitors of cancer cell invasion. Sci Signal.

[43] Bianchi-Smiraglia A, Kunnev D, Limoge M, Lee A, Beckerle MC, Bakin AV (2013). Integrin-β5 and zyxin mediate formation of ventral stress fibers in response to transforming growth factor β. Cell Cycle.

[44] Zhang L, Liu J, Wang X, Li Z, Zhang X, Cao P (2014). Upregulation of cytoskeleton protein and extracellular matrix protein induced by stromal-derived nitric oxide promotes lung cancer invasion and metastasis. Curr Mol Med.

[45] Bisht S, Nolting J, Schütte U, Haarmann J, Jain P, Shah D (2015). Cyclin-Dependent Kinase 5 (CDK5) Controls Melanoma Cell Motility, Invasiveness, and Metastatic Spread-Identification of a Promising Novel therapeutic target. Transl Oncol.

[46] Zhang S, Wang Q, Li W, Chen J (2022). MIR100HG Regulates CALD1 Gene Expression by Targeting miR-142-5p to Affect the Progression of Bladder Cancer Cells in vitro, as Revealed by Transcriptome Sequencing. Front Mol Biosci.

[47] Huhtaniemi R, Oksala R, Knuuttila M, Mehmood A, Aho E, Laajala TD (2018). Adrenals Contribute to Growth of Castration-Resistant VCaP Prostate Cancer Xenografts. Am J Pathol.

[48] Huhtaniemi R, Sipilä P, Junnila A, Oksala R, Knuuttila M, Mehmood A (2022). High intratumoral dihydrotestosterone is associated with antiandrogen resistance in VCaP prostate cancer xenografts in castrated mice. iScience.

[49] Knuuttila M, Yatkin E, Kallio J, Savolainen S, Laajala TD, Aittokallio T (2014). Castration induces up-regulation of intratumoral androgen biosynthesis and androgen receptor expression in an orthotopic VCaP human prostate cancer xenograft model. Am J Pathol.

[50] Sahu B, Laakso M, Pihlajamaa P, Ovaska K, Sinielnikov I, Hautaniemi S (2013). FoxA1 specifies unique androgen and glucocorticoid receptor binding events in prostate cancer cells. Cancer Res.

[51] Arora VK, Schenkein E, Murali R, Subudhi SK, Wongvipat J, Balbas MD (2013). Glucocorticoid receptor confers resistance to antiandrogens by bypassing androgen receptor blockade. Cell.

[52] Isikbay M, Otto K, Kregel S, Kach J, Cai Y, Vander Griend DJ (2014). Glucocorticoid receptor activity contributes to resistance to androgen-targeted therapy in prostate cancer. Horm Cancer.

[53] Xie N, Cheng H, Lin D, Liu L, Yang O, Jia L (2015). The expression of glucocorticoid receptor is negatively regulated by active androgen receptor signaling in prostate tumors. Int J Cancer.

[54] Lam HM, McMullin R, Nguyen HM, Coleman I, Gormley M, Gulati R (2017). Characterization of an Abiraterone Ultraresponsive Phenotype in Castration-Resistant Prostate Cancer Patient-Derived Xenografts. Clin Cancer Res.

[55] Puhr M, Hoefer J, Eigentler A, Ploner C, Handle F, Schaefer G (2018). The Glucocorticoid Receptor Is a Key Player for Prostate Cancer Cell Survival and a Target for Improved Antiandrogen Therapy. Clin Cancer Res.

[56] Polyakov AA, Huber PA, Marston SB, Gusev NB (1998). Interaction of isoforms of S100 protein with smooth muscle caldesmon. FEBS Lett.

[57] D'Angelo G, Graceffa P, Wang CA, Wrangle J, Adam LP (1999). Mammal-specific, ERK-dependent, caldesmon phosphorylation in smooth muscle. Quantitation using novel anti-phosphopeptide antibodies. J Biol Chem.

[58] Yamashiro S, Chern H, Yamakita Y, Matsumura F (2001). Mutant Caldesmon lacking cdc2 phosphorylation sites delays M-phase entry and inhibits cytokinesis. Mol Biol Cell.

[59] Chrétien A, Dierick JF, Delaive E, Larsen MR, Dieu M, Raes M (2008). Role of TGF-beta1-independent changes in protein neosynthesis, p38alphaMAPK, and cdc42 in hydrogen peroxide-induced senescence-like morphogenesis. Free Radic Biol Med.

[60] Xiao D, Singh SV (2008). z-Guggulsterone, a constituent of Ayurvedic medicinal plant Commiphora mukul, inhibits angiogenesis in vitro and in vivo. Mol Cancer Ther.

[61] Guo J, Ma K, Xia HM, Chen QK, Li L, Deng J (2018). Androgen receptor reverts dexamethasone‑induced inhibition of prostate cancer cell proliferation and migration. Mol Med Rep.

[62] Bakir B, Chiarella AM, Pitarresi JR, Rustgi AK (2020). EMT, MET, Plasticity, and Tumor Metastasis. Trends Cell Biol.

[63] Norgard RJ, Pitarresi JR, Maddipati R, Aiello-Couzo NM, Balli D, Li J (2021). Calcium signaling induces a partial EMT. EMBO Rep.

[64] Sobel RE, Sadar MD (2005). Cell lines used in prostate cancer research: a compendium of old and new lines–part 1. J Urol.

[65] Bluemn EG, Coleman IM, Lucas JM, Coleman RT, Hernandez-Lopez S, Tharakan R (2017). Androgen Receptor Pathway-Independent Prostate Cancer Is Sustained through FGF Signaling. Cancer Cell.

[66] Vellky JE, Ricke WA (2020). Development and prevalence of castration-resistant prostate cancer subtypes. Neoplasia..

[67] Abida W, Cyrta J, Heller G, Prandi D, Armenia J, Coleman I (2019). Genomic correlates of clinical outcome in advanced prostate cancer. Proc Natl Acad Sci USA.

[68] Kumar A, Coleman I, Morrissey C, Zhang X, True LD, Gulati R (2016). Substantial interindividual and limited intraindividual genomic diversity among tumors from men with metastatic prostate cancer. Nat Med.

[69] Grasso CS, Wu YM, Robinson DR, Cao X, Dhanasekaran SM, Khan AP (2012). The mutational landscape of lethal castration-resistant prostate cancer. Nature..

[70] Robinson D, Van Allen EM, Wu YM, Schultz N, Lonigro RJ, Mosquera JM (2015). Integrative clinical genomics of advanced prostate cancer. Cell.

[71] Taylor BS, Schultz N, Hieronymus H, Gopalan A, Xiao Y, Carver BS (2010). Integrative genomic profiling of human prostate cancer. Cancer Cell.

[72] Armenia J, Wankowicz SAM, Liu D, Gao J, Kundra R, Reznik E (2018). The long tail of oncogenic drivers in prostate cancer. Nat Genet.

[73] Crowdis J, Balch S, Sterlin L, Thomas BS, Camp SY, Dunphy M (2022). A patient-driven clinicogenomic partnership for metastatic prostate cancer. Cell Genom.

[74] The ICGC/TCGA Pan-Cancer Analysis of Whole Genomes Consortium. (2020). Pan-cancer analysis of whole genomes. Nature.

[75] Baca SC, Prandi D, Lawrence MS, Mosquera JM, Romanel A, Drier Y (2013). Punctuated evolution of prostate cancer genomes. Cell.

[76] Beltran H, Prandi D, Mosquera JM, Benelli M, Puca L, Cyrta J (2016). Divergent clonal evolution of castration-resistant neuroendocrine prostate cancer. Nat Med.

[77] Barbieri CE, Baca SC, Lawrence MS, Demichelis F, Blattner M, Theurillat JP (2012). Exome sequencing identifies recurrent SPOP, FOXA1 and MED12 mutations in prostate cancer. Nat Genet.

[78] Ren S, Wei GH, Liu D, Wang L, Hou Y, Zhu S (2018). Whole-genome and Transcriptome Sequencing of Prostate Cancer Identify New Genetic Alterations Driving Disease Progression. Eur Urol.

[79] Gao D, Vela I, Sboner A, Iaquinta PJ, Karthaus WR, Gopalan A (2014). Organoid cultures derived from patients with advanced prostate cancer. Cell.

