# Supplementary material for: Glucocorticoid receptor-induced non-muscle caldesmon regulates metastasis in castration-resistant prostate cancer
Source: Oncogenesis. 2023 Aug 12;12(1):42. doi: 10.1038/s41389-023-00485-z (PMC10423232; doi:10.1038/s41389-023-00485-z)
Supplement: Supplementary file 1 — Supplementary Information [file 41389_2023_485_MOESM1_ESM.docx]

**Supplementary materials and methods**

*Cell lines and culture*

PC3 (ATCC, Manassas, VA) and DU145 (ATCC) cells were cultured in DMEM (Gibco, Waltham, MA) supplemented with heat-inactivated 10% fetal bovine serum (FBS) (Gibco). VCaP (ATCC) cells were cultured in DMEM (Gibco) supplemented with non-inactivated 10% FBS (Gibco). PPC-1 (ATCC) cells were cultured in RPMI 1640 (Gibco) supplemented with heat-inactivated 10% FBS (Gibco) and 2 mM glutamine (Lonza, Quakertown, PA). RWPE-1 (ATCC) cells were cultured in Keratinocyte Serum Free Medium (K-SFM) (Gibco) supplemented with bovine pituitary extract (Gibco) and human recombinant epidermal growth factor (Gibco). LNCaP (ATCC) and 22Rv1 (ATCC) cells were cultured in RPMI 1640 (Gibco) supplemented to a final concentration of 10% heat-inactivated FBS (Gibco), 2 mM glutamine (Lonza), 4500 mg/l D-glucose (Sigma-Aldrich, Burlington, MA), 10 mM HEPES (Lonza) and 1 mM sodium pyruvate (Gibco). The mCherry-expressing PC3 cell line was created by infecting PC3 cells using mCherry lentivector (Addgene, Watertown, MA; pLV-mCherry, pLenti-MP2, #36084) followed by the selection of mCherry-expressing cells by fluorescence-activated cell sorting. All cell lines were cultured in the presence of 1% penicillin-streptomycin solution (Sigma-Aldrich) except during experiments. All cell lines were routinely tested to be mycoplasma free and checked for authenticity.

*RNA interference*

*Silencer*^®^ Select siRNA oligonucleotides targeting human CaD (Ambion, Austin, TX; *CALD1* siRNA#2337, “siCaD-1”; siRNA#2339, “siCaD-2”) and negative control siRNA oligonucleotides (Ambion; Neg CTRL#1, “siNeg1”; Neg CTRL#2, “siNeg2”) were used for transfection. PC3 cells were transfected 24 h after seeding using siRNA at a final concentration of 2.5 nM with 0.125% DharmaFECT 2 (Dharmacon, Lafayette, CO), DU145 cells 24 h after seeding using 5 nM siRNA with 0.125% DharmaFECT 1 (Dharmacon), and VCaP cells 3 h after seeding using 10 nM siRNA with 0.5% DharmaFECT 3 (Dharmacon). Transfection solutions were prepared in Opti-MEM (Gibco). The efficiency of downregulation was verified 48 h after transfection by Western blotting.

*Cell lysis and Western blotting*

Cells were lysed into lysis buffer containing 1% Triton X-100, 20 mM Tris-HCl, (pH 7.5), 1 mM EDTA, 150 mM NaCl, and 10 mM NaF. Lysis buffer was supplemented with 2% cOmplete EDTA-free protease inhibitor cocktail (Roche, Penzberg, Germany), 10 mM Na_4_P_2_O_7_, and 1 mM Na_3_VO_4_ just prior to the lysis of cells. The lysates were centrifuged, and 30–100 μg samples of total protein from the supernatants were separated on 4–15% SDS-PAGE gels (Biorad, Hercules, CA). The separated proteins were transferred onto a nitrocellulose membrane (Santa Cruz Biotechnology, Dallas, TX). Nonspecific binding to the membrane was blocked by an hour incubation in TBST (10 mM Tris-HCl, pH 7.4, 150 mM NaCl, 0.05% Tween-20) buffer containing 5% nonfat dry milk and 1% bovine serum albumin (BSA) (Sigma-Aldrich). Membranes were incubated overnight (4°C) with antibodies for CaD (Cell Signaling, Danvers, MA; #12503, 1:1000), glucocorticoid receptor (Cell Signaling; #12041, 1:1000), actin (Santa Cruz Biotechnology; sc-1616, 1:1000), and vinculin (Santa Cruz Biotechnology; sc-73614, 1:1000,) diluted in TBST containing 5% BSA (Sigma-Aldrich). Membranes were incubated for an hour at room temperature (RT) with peroxidase-conjugated goat anti-rabbit IgG and goat anti-rabbit IgG secondary antibodies (Santa Cruz Biotechnology; 1:10,000) diluted in TBST containing 5% milk and 1% BSA (Sigma-Aldrich). Bound secondary antibody was visualized by enhanced chemiluminescence WesternBright Quantum kit (Advansta, San Jose, CA) and captured with LAS-4000 (Fujifilm, Tokyo, Japan) or ChemiDoc™ Touch Gel Imaging System (Bio-Rad, Hercules, CA).

*3D growth assays*

Cells were trypsinized 48 h after siRNA transfection, reconstituted in 25% basement membrane matrix (Corning, Corning, NY; Matrigel^®^, Growth Factor Reduced), and seeded in 96-well plates precoated with 50% basement membrane matrix (40 µl cell layer and 30 µl precoat /well) (Corning). 100 µl of culture media was added on top of the cell layer and the precoat after 1 h incubation at + 37 °C. Media was replaced every 1­–3 days. For the treated wells, dexamethasone (Abmole, Houston, TX or Selleckchem, Houston, TX; 1 µM) or vehicle (DMSO) was added on day 3. Duplicate wells were imaged on days 5 and 7 for a minimum of three 4x brightfield images per well using EVOS M5000 (Thermo Fisher Scientific, Waltham, MA). Spheroid size was measured in ImageJ with segmenting by manual thresholding and manual exclusion of spheroids merged due to high initial proximity.

*MTT assays*

The viability of cells grown in triplicate wells was determined using CellTiter 96® AQueous Non-Radioactive Cell Proliferation MTS Assay (Promega, Madison, WI; G5430) according to product protocol, and the absorbance was measured at 490 nm using a Wallac Victor2 1420 Multilabel Counter (PerkinElmer, Waltham, MA). Assays were performed 48 h after transfection for PC3 and VCaP cells and 72 h after CSS treatment for VCaP cells.

*Zebrafish xenografts*

Analyses of zebrafish embryos were carried out under the licenses MMM/465/712–93 (issued by the Finnish Ministry of Agriculture and Forestry) and ESAVI/9339/04.10.07/2016 (granted by Project Authorization Board of Regional State Administrative Agency for Southern Finland) according to the regulations of the Finnish Act on Animal Experimentation (62/2006). The study was carried out in compliance with the ARRIVE guidelines. Zebrafish embryos were obtained using natural spawning of the fish in breeding tanks. The embryos were cultured in E3 + PTU medium at 28.5 °C until subjected to mCherry PC3, DU145, or LNCaP microinjection using Nanoject II microinjector (Drummond Scientific, Broomall, PA). DU145 and LNCaP were labeled with CellTracker Green CFMDA (Invitrogen, Waltham, MA) prior to the microinjection. After microinjection, embryos were transferred into 33 °C incubator with E3 + PTU + PenStrep. The embryos were anesthetized and imaged using Zeiss AxioZoom V16 (Zeiss, Oberkochen, Germany) or Nikon Eclipse Ti2 (Nikon, Tokyo, Japan) at 1 day after injection (final endpoint for the common cardinal vein model) and again at 4 days after injection. Data from images was extracted in ImageJ. All measurements were done independently by two investigators blinded to the group allocation.

*2D culture immunofluorescence staining*

Monolayer-cultured cells were fixed with 4% paraformaldehyde for 10 min, washed with PBS 3 times 5 min, and permeabilized with 0.25% Triton X-100 for 10 min followed by 3 times 5 min PBS washes. After 1 h blocking in 3% BSA in PBS buffer at RT, glasses were incubated in blocking solution containing antibody against CaD (Cell Signaling; #12503, 1:200) overnight at + 4 °C. After washing 3 times 5 min using 100 µl droplets of PBS, glasses were incubated for 1 h in blocking buffer containing anti-rabbit Alexa Fluor 488 (Invitrogen; A-11034, 1:1000) at RT. Before mounting glasses on slides in Vectashield (Vector Laboratories, Burlingame, CA), slides were washed with PBS for 5 min, incubated with Phalloidin 647 (1:200 in PBS), and washed with PBS containing 1:1000 DAPI for 5 min. The cells were imaged using Nikon Eclipse Ni (Nikon). Secondary controls incubated without primary antibody and phalloidin were used for each biological replicate to determine the threshold for autofluorescence and signal from unspecific secondary antibody binding.

*3D spheroid immunofluorescence staining*

After washing wells with PBS, 2% paraformaldehyde was added and incubated for 15 min, followed by transferring the spheroids in Eppendorf tubes using transfer pipettes and washing 3 times 5 min using 3D wash buffer containing 0.2% Triton X-100, 0.05% Tween-20, and 0.1% BSA in PBS. Spheroids were permeabilized using 0.25% Triton X-100 for 10 min followed by 3 times 5 min 3D wash buffer washes. Spheroids were blocked at RT with 3D wash buffer containing 2.5% BSA and incubated overnight at 4 °C on a rotator with antibody against N-Cadherin (Cell Signaling; #13116, 1:200), E-cadherin (Cell Signaling; #3195, 1:1600), and ZEB1 (Cell Signaling; #70512, 1:400) diluted in 3D wash buffer. Spheroids were washed 3 times 15 min on a rotator before 1 h incubation with 3D wash buffer containing anti-rabbit Alexa Fluor 488 antibody (Invitrogen; A-11034, 1:1000) or anti-rabbit Alexa Fluor 555 antibody (Invitrogen; A-21329, 1:1000) at RT on a rotator followed by 3 times 15 min washes with the last wash containing 1:1000 DAPI. For imaging, spheroids were moved in a 96-well plate in Vectashield (Vector Laboratories) and imaged using 3i CSU-W1 spinning disk confocal microscope (Yokogawa, Tokyo, Japan). N-cadherin, ZEB1, and E-cadherin intensities were analyzed in ImageJ by measuring the mean intensity of individual spheroids from sum projections after substracting image background intensity from the measure area.

*Zebrafish embryo immunofluorescence staining*

Zebrafish embryos were stained using a previously described protocol with some modifications [1]. Zebrafish embryos with xenografts of PC3mCherry cells transfected with control or l-CaD targeting siRNA were fixed with 4% paraformaldehyde overnight 4 °C and washed 3 times with PBST (PBS + 0.2% Tween-20). Fixed embryos were treated with 0.25% trypsin-EDTA (Gibco) and washed 3 times with PBST. Embryos were permeabilized using 2% Triton X-100 (Sigma-Aldrich) for 1 h and washed 3 times with PBST. Embryos were blocked overnight at 4 °C in PBSTx (PBST + 0.2% Triton X-100) containing 5% FBS and 1% BSA. After blocking, embryos were incubated overnight in primary antibodies against N-cadherin (Cell Signaling; #13116, 1:200) and CaD (Santa Cruz Biotechnology; sc-25339, 1:200) diluted in blocking solution and washed 3 times with PBST. Secondary antibodies anti-rabbit Alexa Fluor 488 (Invitrogen; A-11034) and anti-mouse Alexa Fluor 555 (Invitrogen; A-21422) were used in 1:1000 dilution with 1:2000 DAPI (Sigma-Aldrich) overnight in blocking solution. After overnight incubation, embryos were washed 4 times 60 min with PBSTx. Before confocal microscope visualization, embryos were mounted on glass bottom dishes in low-melting point agarose. Embryos were visualized with 3i CSU-W1 (Yokogawa) spinning disk confocal microscope.

*CSS, DHT, dexamethasone, prednisolone, and enzalutamide lead-in treatment*

CSS treatments of VCap cells were carried out 24 h after seeding or 48 h after transfection by washing two times and replacing the old media with media supplemented with 5% charcoal-stripped FBS (Gibco). 10 nM DHT (Sigma-Aldrich) or vehicle (methanol) was added after 48 h, and cells were lysed after 24 h of DHT treatment.

PC3 and DU145 cells were treated with 0.1, 1, 10 µM dexamethasone or vehicle (DMSO) 20 h after seeding and the cells were lysed after 24 or 48 h of treatment. DU145 cells were treated with 0.1, 1, 10 µM prednisolone (Abmole) or vehicle (DMSO) 20 h after seeding and the cells were lysed after 48 h of treatment.

VCaP cells were treated with 10 µM enzalutamide (Abmole or Selleckchem) or vehicle (DMSO) (MP Biomedicals, Irvine, CA) 24 h after seeding. After five days of enzalutamide treatment, 0.1 µM dexamethasone or vehicle (DMSO) was added, and cells were lysed after 24 h.

*VCaP xenograft mouse models and IHC*

Orthotopic [2] and subcutaneous [3, 4] castration-resistant xenograft models have been previously generated. Sections of xenograft tumors were stained using antibodies against CaD (Abcam, Cambridge, United Kingdom; ab32330, 1:50) and GR (Cell Signaling; #12041, 1:200). Staining conditions were optimized for both antibodies before staining the VCaP xenograft samples.

*Statistical analyses*

Statistical analyses were performed using R studio (R Project for Statistical Computing) and GraphPad Prism 8.4.2 software. The distribution of genotypes was analyzed with the Chi-square test and allele frequencies with the binomial test. The Spearman’s rank correlation coefficient was used for analysis of correlation. Data extracted from cBioPortal was generated using the default methods of the v4.1.9. Two-tailed unpaired Student’s *t*-test, Welch’s *t*-test, or the Mann–Whitney–Wilcoxon two-sided test were used in comparisons between two groups. The comparisons for categorical data between two groups were performed using Fisher’s exact test.

**References**

1. Inoue D, Wittbrodt J. One for all--a highly efficient and versatile method for fluorescent immunostaining in fish embryos. PLoS One. 2011; 6:e19713.
2. Knuuttila M, Yatkin E, Kallio J, Savolainen S, Laajala TD, Aittokallio T, et al. Castration induces up-regulation of intratumoral androgen biosynthesis and androgen receptor expression in an orthotopic VCaP human prostate cancer xenograft model. Am J Pathol. 2014; 184:2163–73.
3. Huhtaniemi R, Oksala R, Knuuttila M, Mehmood A, Aho E, Laajala TD, et al. Adrenals Contribute to Growth of Castration-Resistant VCaP Prostate Cancer Xenografts. Am J Pathol. 2018; 188:2890–2901.
4. Huhtaniemi R, Sipilä P, Junnila A, Oksala R, Knuuttila M, Mehmood A, et al. High intratumoral dihydrotestosterone is associated with antiandrogen resistance in VCaP prostate cancer xenografts in castrated mice. iScience. 2022; 25:104287.
