# Supplementary material for: Glucocorticoid receptor-induced non-muscle caldesmon regulates metastasis in castration-resistant prostate cancer
Source: Oncogenesis. 2023 Aug 12;12(1):42. doi: 10.1038/s41389-023-00485-z (PMC10423232; doi:10.1038/s41389-023-00485-z)
Supplement: Supplementary file 2 — Supplementary Figures [file 41389_2023_485_MOESM2_ESM.pdf]

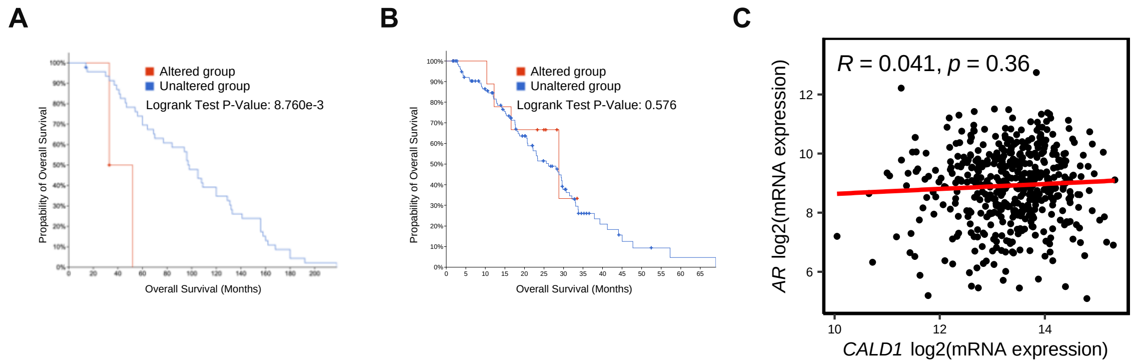

Supplementary Figure S1. Correlations of *CALD1* alterations and expression with poor prognosis and *AR* in PCa patient data sets.

(A) Kaplan-Meier overall survival curves between patients with *CALD1* amplifications (n = 2) and unaltered *CALD1* (n = 46) (70).

(B) Kaplan-Meier overall survival curves between patients with *CALD1* alterations (n = 10) and unaltered *CALD1* (n = 118) (68).

(C) Scatterplot depicting the Spearman's rank correlation between *CALD1* and *AR* in TCGA PCa data set.

A

EPITHELIAL CELL PROLIFERATION

ITGB3	0.811****	*PKHD1*	0.202****	*ZNF580*	-0.225****
FGF2	0.794****	*VASH2*	0.2****	*HRAS*	-0.246****
FZD7	0.788****	*PDGFB*	0.189****	*BAD*	-0.247****
CAV2	0.763****	*THBS4*	0.189****	*MYDGF*	-0.255****
NRP2	0.763****	*PTPRN*	0.188****	*SIRT6*	-0.258****
FGFR1	0.69****	*HIF1A*	0.184****	*AKT1*	-0.273****
NOG	0.659****	*CCR3*	0.183****	*APLN*	-0.285****
SFRP1	0.637****	*NOTCH2*	0.173***	*EMC10*	-0.291****
SCN5A	0.63****	*ODAM*	0.173***	*IQGAP3*	-0.291****
FGF7	0.627****	*SP1*	0.171***	*PDCL3*	-0.335****
FGF10	0.615****	*CYP7B1*	0.168***	*GATA2*	-0.342****
SEMA5A	0.597****	*SHH*	0.166***	*NME2*	-0.356****
HTRA1	0.593****	*ADAM17*	0.162***	*ESRP2*	-0.371****
JCAD	0.593****	*VIP*	0.151***	*MST1*	-0.373****
KDR	0.589****	*SCG2*	0.15***	*SRSF6*	-0.413****
YAP1	0.579****	*APLNR*	0.146**	*PDCD6*	-0.424****
TLR4	0.568****	*MMP12*	0.14**	*NME1*	-0.465****
FGFR2	0.549****	*NR4A1*	0.138**	*HPN*	-0.517****
TEK	0.545****	*GRN*	0.135**		
STAT5A	0.544****	*CCL24*	0.131**		
BMP4	0.542****	*RICTOR*	0.13**		
ECM1	0.531****	*CCL26*	0.128**		
PRKCA	0.512****	*EGFL7*	0.125**		
FGF1	0.489****	*HMOX1*	0.116*		
OSR1	0.483****	*F3*	0.112*		
CDH3	0.476****	*CCL11*	0.109*		
PIK3CD	0.474****	*EPGN*	0.1*		
TP63	0.458****	*VEGFB*	0.1*		
CXCL12	0.454****	*CDC42*	0.097*		
IGF2	0.443****	*CCND1*	0.092*		
NOTCH1	0.435****	*NRP1*	0.089*		
PTN	0.433****	*EGF*	0.086ns		
HYAL1	0.43****	*SIRT1*	0.084ns		
FLT4	0.429****	*LACRT*	0.083ns		
HAS2	0.425****	*CRNN*	0.077ns		
LAMB1	0.414****	*VEGFD*	0.071ns		
NOD2	0.407****	*REG3G*	0.068ns		
TGFBR1	0.399****	*WNT5A*	0.062ns		
TNFAIP3	0.394****	*GHRL*	0.061ns		
OSR2	0.389****	*VEGFA*	0.056ns		
B4GALT1	0.368****	*PLCG1*	0.055ns		
PGF	0.366****	*MTOR*	0.052ns		
PROX1	0.366****	*SOX9*	0.046ns		
TGM1	0.366****	*AR*	0.044ns		
ITGA4	0.361****	*MED1*	0.044ns		
RPTOR	0.359****	*ZNF304*	0.043ns		
JAML	0.354****	*MAP2K5*	0.04ns		
WNT3A	0.351****	*ATP7A*	0.038ns		
BMPR2	0.349****	*ZNF703*	0.037ns		
TGFA	0.349****	*RREB1*	0.033ns		
EGFR	0.343****	*REG3A*	0.026ns		
TNFSF12	0.341****	*ARG1*	0.022ns		
WNT2	0.324****	*PRKD1*	0.018ns		
CDH13	0.32****	*GDF2*	0.012ns		
STXBP4	0.319****	*GHSR*	0.01ns		
ID1	0.315****	*SMO*	0.009ns		
FGF9	0.313****	*AGGF1*	0.008ns		
PPP1R16B	0.305****	*DLX5*	0.003ns		
BMP2	0.304****	*DLX6*	0.001ns		
CLDN1	0.303****	*MDK*	0ns		
ARNT	0.299****	*NRAS*	0ns		
AKT3	0.294****	*IHH*	-0.004ns		
WDR48	0.292****	*LRG1*	-0.004ns		
BMPR1A	0.288****	*PDPK1*	-0.018ns		
NR4A3	0.28****	*XBP1*	-0.027ns		
CFLAR	0.278****	*PRKD2*	-0.047ns		
FGFBP1	0.277****	*REG1A*	-0.05ns		
GPBAR1	0.272****	*SOX11*	-0.057ns		
RTN4	0.265****	*HMGB2*	-0.061ns		
ANG	0.251****	*ERBB2*	-0.067ns		
LAMC1	0.25****	*HMGB1*	-0.082ns		
WNT7A	0.25****	*KDM5B*	-0.085ns		
C5AR2	0.247****	*BMP6*	-0.093*		
IGF1	0.245****	*AGTR1*	-0.096*		
VEGFC	0.24****	*PAX2*	-0.122**		
TCF7L2	0.232****	*ZBED9*	-0.137**		
NODAL	0.226****	*TWIST1*	-0.138**		
EGR3	0.225****	*MST1L*	-0.142**		
C5AR1	0.218****	*TBX1*	-0.145**		
BMP5	0.217****	*FOXE3*	-0.156***		
AREG	0.213****	*NRARP*	-0.169***		
IL10	0.208****	*MYC*	-0.183****		
HTR2B	0.205****	*PLXNB3*	-0.218****		

GOBP_POSITIVE_REGULATION_OF_EPITHELIAL_CELL_PROLIFERATION

CAV1	0.79****	*TGFB1*	0.422****
CAV2	0.763****	*GPC3*	0.412****
TGFBR3	0.752****	*CDKN2B*	0.405****
GJA1	0.744****	*TGFBR1*	0.399****
LIMS2	0.703****	*KLF9*	0.395****
WFDC1	0.697****	*ATOH8*	0.389****
ISL1	0.693****	*TNMD*	0.386****
MEF2C	0.674****	*FLT1*	0.375****
RGN	0.661****	*THBS1*	0.361****
SERPINF1	0.651****	*CEACAM1*	0.359****
MCC	0.647****	*DLL4*	0.338****
SFRP1	0.637****	*SOX2*	0.333****
SULF1	0.623****	*MTSS1*	0.326****
SNAI2	0.559****	*KRT4*	0.325****
NR2F2	0.555****	*B2M*	0.294****
TGFB2	0.555****	*RGCC*	0.287****
FGFR2	0.549****	*PPARG*	0.282****
ROBO1	0.549****	*PTEN*	0.276****
GDF5	0.548****	*IL12A*	0.274****
BMP4	0.542****	*RUNX3*	0.274****
COL4A3	0.515****	*CDKN1C*	0.271****
SPARC	0.5****	*NF1*	0.252****
DAB2IP	0.479****	*CCL2*	0.25****
NGFR	0.457****	*EREG*	0.244****
SFRP2	0.457****	*VASH1*	0.24****
GATA3	0.441****	*PTPRK*	0.225****
CD109	0.433****	*CNMD*	0.222****
PTN	0.433****	*TNF*	0.217****
		VDR	0.204****
		ATF2	0.196****
		ALOX5	0.191****
		RB1	0.189****
		NLRC3	0.186****
		A4GNT	0.183****
		PTPRM	0.183****
		SFN	0.169***
		PPARD	0.164***
		STAT1	0.163***
		PTCH1	0.161***
		EPPK1	0.151***
		SCG2	0.15***
		BRCA2	0.148**
		NKX2-8	0.146**
		CDC73	0.143**
		CASK	0.142**
		FBXW7	0.123**

GOBP_NEGATIVE_REGULATION_OF_EPITHELIAL_CELL_PROLIFERATION

NFIB	0.123**
IFT52	0.118**
DUSP10	0.112*
CDK6	0.1*
SAV1	0.093*
XDH	0.077ns
IL12B	0.064ns
WNT5A	0.062ns
GHRL	0.061ns
DLG1	0.048ns
SOX9	0.046ns
AR	0.044ns
MED1	0.044ns
SUZ12	0.025ns
SYNJ2BP	0.018ns
GDF2	0.012ns
IL26	0.011ns
KRIT1	0.009ns
INTU	-0.005ns
IFT172	-0.018ns
PRL	-0.018ns
IFT57	-0.019ns
SLURP1	-0.025ns
APOE	-0.028ns
CPB2	-0.04ns
PDCD10	-0.044ns
IFT80	-0.056ns
MARVELD3	-0.082ns
STRAP	-0.092*
TINF2	-0.101*
APOH	-0.106*
PBLD	-0.106*
WNT10B	-0.111*
PEX2	-0.115*
AQP11	-0.127**
ATP5F1A	-0.16***
NUPR1	-0.184****
NKX3-1	-0.194****
MAGED1	-0.219****
PHB2	-0.228****
STK11	-0.232****
IFT74	-0.251****
AIMP1	-0.306****
WDR13	-0.317****
ATP5IF1	-0.365****
WDR77	-0.396****
KDF1	-0.485****
HPN	-0.517****

0.8

0.4

0

-0.4

-0.8

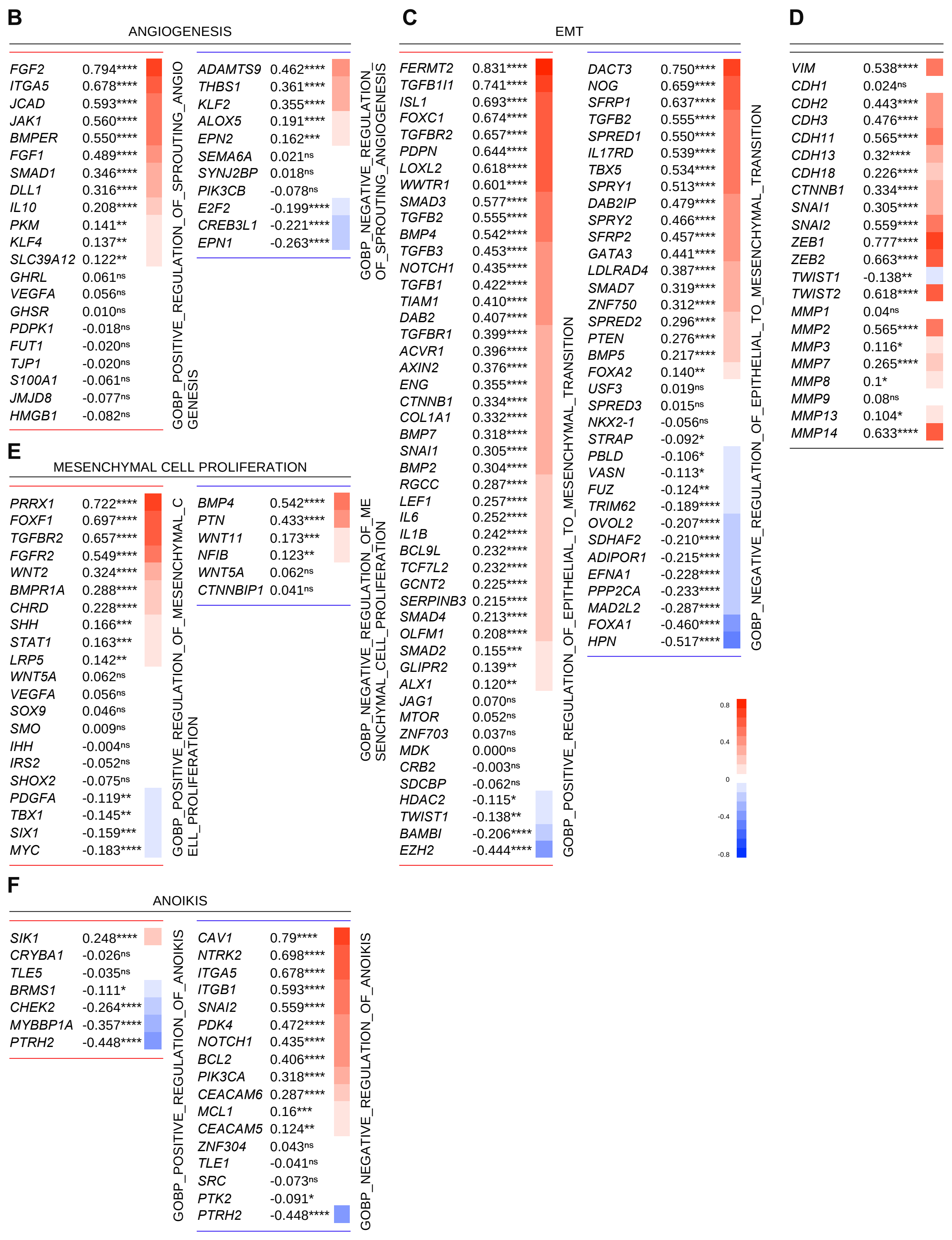

Supplementary Figure S2. Co-expression between *CALD1* and regulators of proliferation, angiogenesis, EMT, and anoikis in the prostate TCGA data set.
(A) List of transcripts from the Gene Ontology: Biological Processes gene set for positive and negative regulators of epithelial cell proliferation sorted according to Spearman's correlation values.
(B) List of transcripts from the Gene Ontology: Biological Processes gene set for positive and negative regulators of angiogenesis sorted according to Spearman's correlation values.

(C) List of transcripts from the Gene Ontology: Biological Processes gene set for positive and negative regulators of EMT sorted according to Spearman's correlation values.

(D) A custom list featuring markers associated with epithelial and markers associated with mesenchymal cell phenotype.

(E) List of transcripts from the Gene Ontology: Biological Processes gene set for positive and negative regulators of mesenchymal cell proliferation sorted according to Spearman's correlation values.

(F) List of transcripts from the Gene Ontology: Biological Processes gene set for positive and negative regulators of anoikis sorted according to Spearman's correlation values.

Spearman's rank correlation coefficient presented as numbers and significance indicated by asterisks (ns = not significant, *$p < 0.05$, **$p < 0.01$, *** $p < 0.001$, and ****$p < 0.0001$ as obtained using cBioPortal v4.1.9).

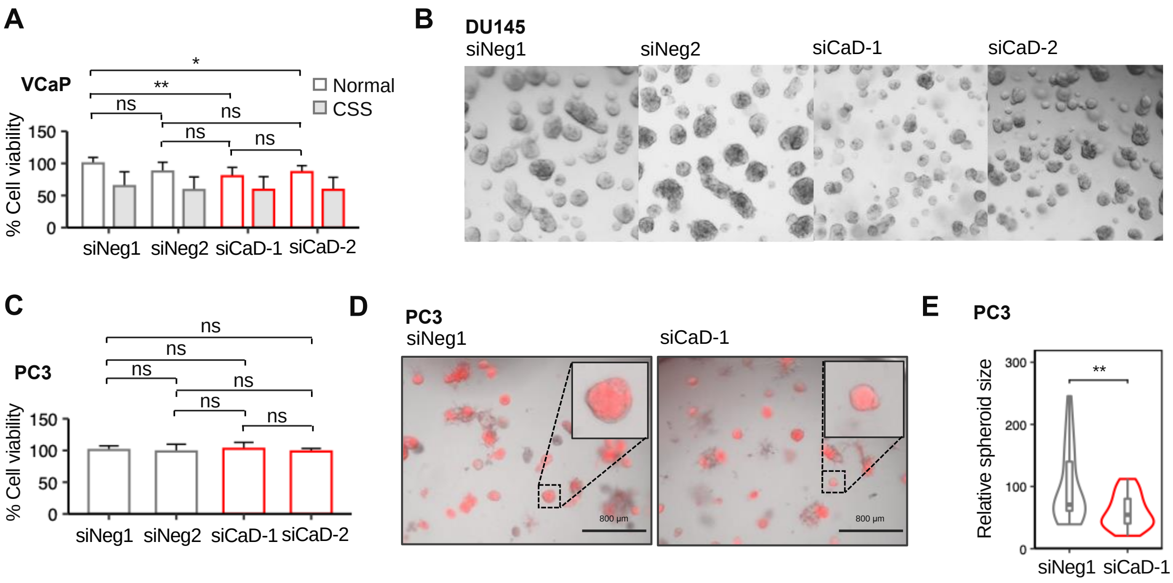

Supplementary figure S3. Viability and 3D growth after l-CaD knockdown.

(A) Barplot representing viability in VCaP cells transfected with siNeg1, siNeg2, siCaD-1, or siCaD-2 and cultured with or without CSS. Viability of CSS-treated cells is highlighted with grey fill. The mean and SD of three experiments are shown (ns = not significant, $*p < 0.05$, and $**p < 0.01$ as determined by the Mann–Whitney–Wilcoxon two-sided test).

(B) Representative brightfield images of DU145 cells cultured in 3D after l-CaD knockdown.

(C) Barplot representing viability in mCherry PC3 cells transfected with siNeg1, siNeg2, siCaD-1, or siCaD-2. The mean and SD of triplicate wells are shown (ns = not significant as determined by the Mann–Whitney–Wilcoxon two-sided test).

(D) Representative brightfield and fluorescent merge images of mCherry PC3 cells transfected with siNeg1 or siCaD-1 when grown in 3D basement membrane matrix culture.

(E) Violin plots of relative spheroid size in mCherry PC3 cells transfected with siNeg1 or siCald-1 and grown in 3D Matrigel culture. Boxplot indicates median, and whiskers indicate 1.5 times interquartile range ($**p < 0.01$ as determined by t-test).

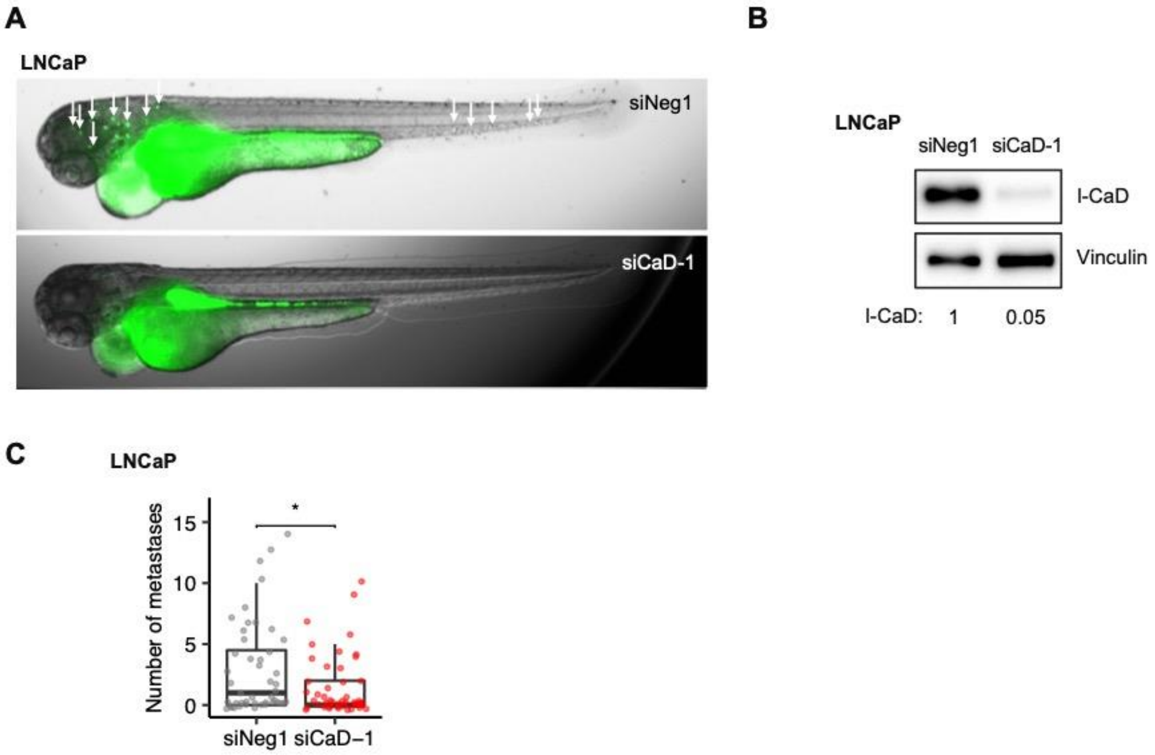

Supplementary figure S4. l-CaD knockdown impairs metastatic potential of LNCaP cells injected into zebrafish.

(A) Representative brightfield and fluorescent merge images of zebrafish embryos 1 day after microinjection of CellTracker Green labeled LNCaP cells transfected with siNeg1 or siCaD-1 to the common cardinal vein. Metastases are indicated with arrows.

(B) Expression levels of l-CaD in LNCaP cells transfected with siNeg1 or siCaD-1 48 h after transfection were analyzed by Western blotting using an anti-CaD antibody and loading was controlled using anti-vinculin.

(C) Box and jitter plots depicting the number of metastases in zebrafish embryos (n = 96). Boxplot indicates median, and whiskers indicate 1.5 times interquartile range (* $p < 0.05$ as determined by *t*-test).

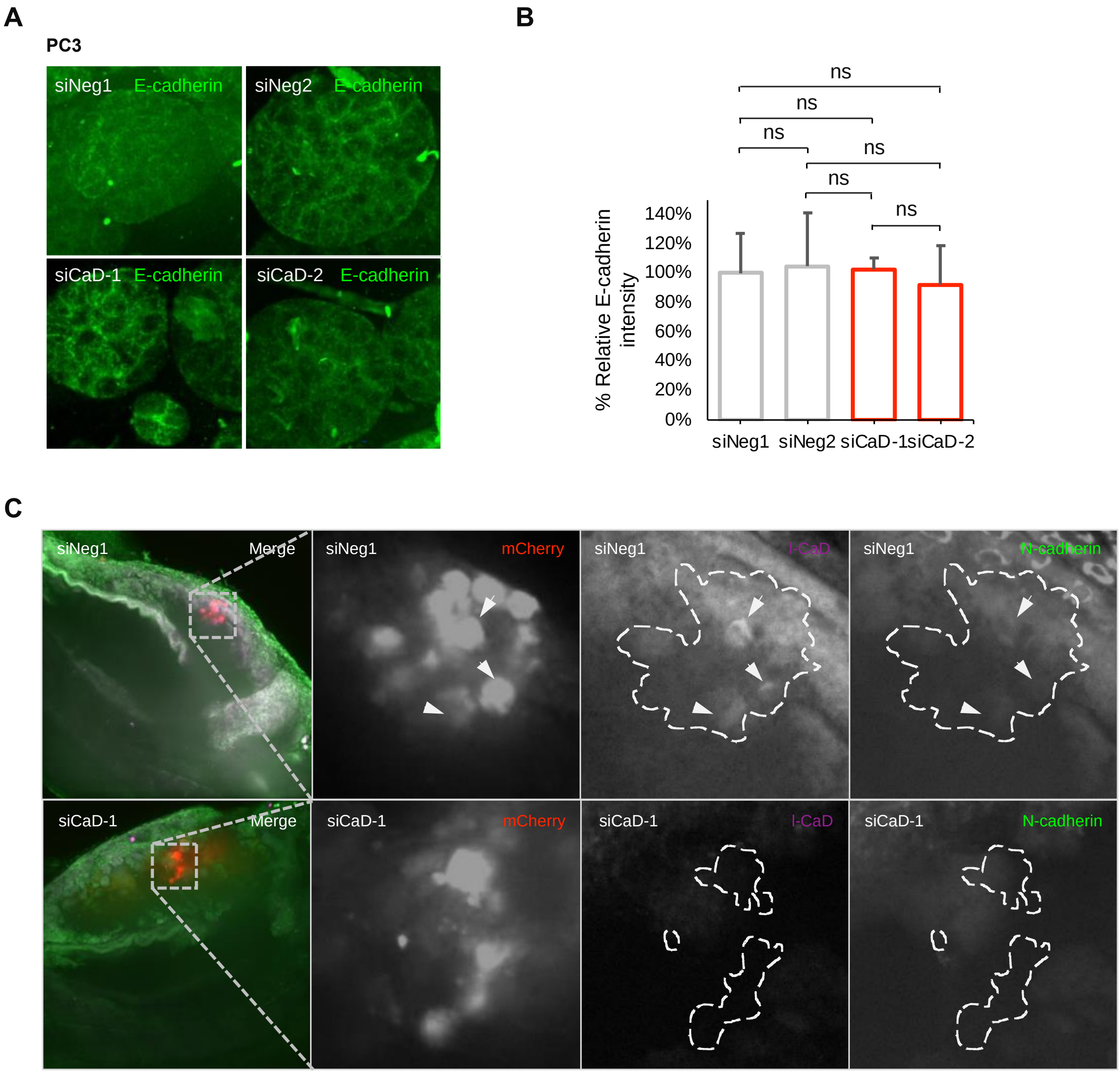

Supplementary Figure S5. E-cadherin expression in PC3 cell spheroids after l-CaD knockdown *in vitro* and N-cadherin expression in mCherry PC3 cell primary tumors after l-CaD knockdown in zebrafish xenografts *in vivo*.

(A) Representative fluorescent images of PC3 spheroids transfected with siNeg1, siNeg2, or siCaD-1, siCaD-2, stained with E-cadherin, and counterstained with DAPI.

(B) Barplots depicting E-cadherin protein intensity in spheroids formed by transfected PC3 cells shown in figure A. Average was calculated from multiple individual spheroids (N = 25) measured for mean intensity. The mean and SD are shown (ns = not significant as determined by *t*-test).

(C) Representative fluorescent images of primary tumors formed in zebrafish yolk sacs from injected PC3 cells transfected with siNeg1 or siCaD-1 stained for l-CaD and N-cadherin. Merge image shows the primary tumor in the yolk sac of the zebrafish.

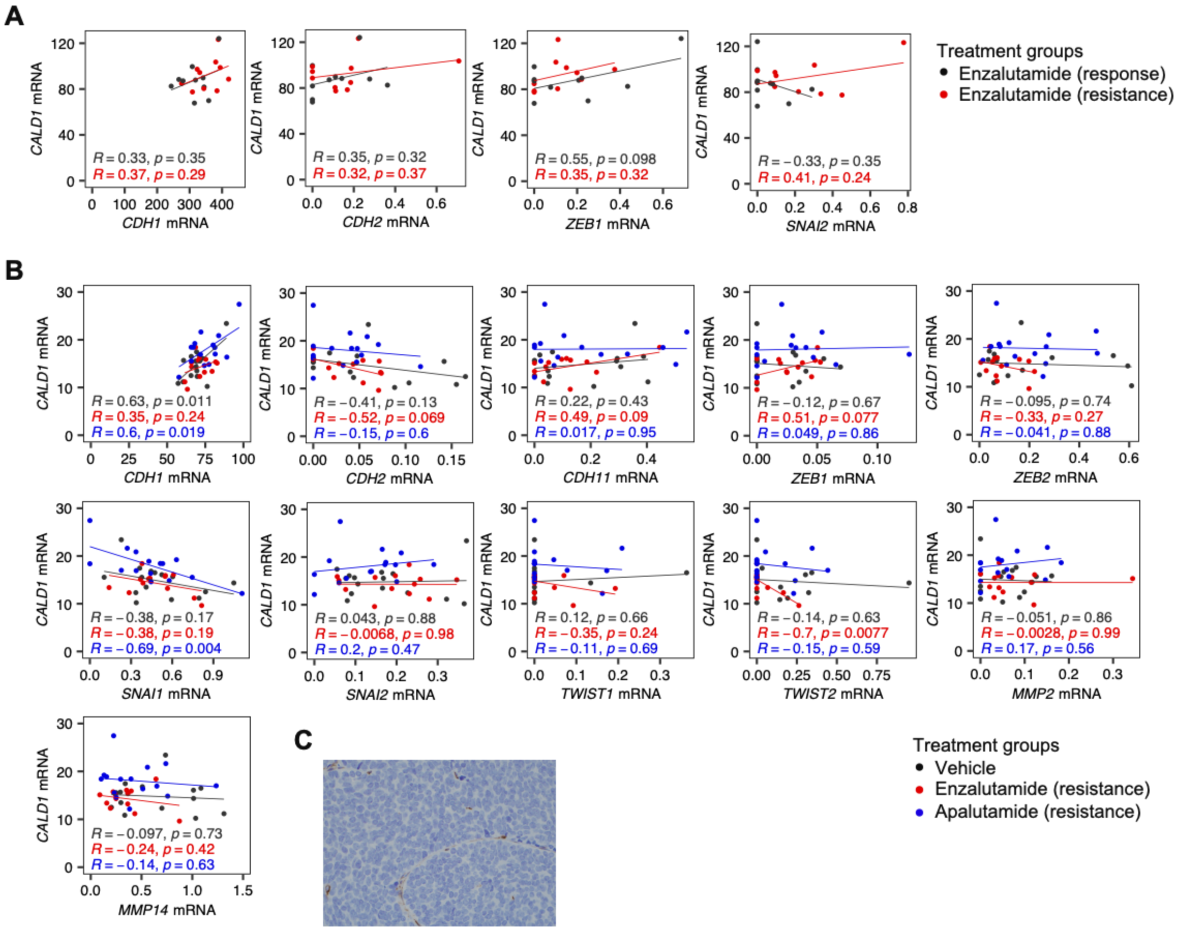

Supplementary Figure S6. EMT-related marker expression in VCaP xenograft mouse models.

(A) Scatterplots depicting the correlation between *CALD1* and EMT marker mRNAs from enzalutamide-treated VCaP xenograft mice.

(B) Scatterplots depicting the correlation between *CALD1* and EMT marker mRNAs from vehicle-, enzalutamide- and apalutamide-treated VCaP xenograft mice.

(C) Representative IHC staining of vimentin from a castration-resistant subcutaneous VCaP xenograft mouse resistant to enzalutamide.
