# Supplementary material for: Glucocorticoid receptor-induced non-muscle caldesmon regulates metastasis in castration-resistant prostate cancer
Source: Oncogenesis. 2023 Aug 12;12(1):42. doi: 10.1038/s41389-023-00485-z (PMC10423232; doi:10.1038/s41389-023-00485-z)
Supplement: Supplementary file 3 — Supplementary Tables [file 41389_2023_485_MOESM3_ESM.pdf]

Supplementary Table 1. Co-expression analysis between *CALD1* and regulators of cancer hallmarks, actin-related processes and relevant signaling pathways (MSigDB, Gene Ontology: Biological Processes) in the PCa TCGA data set.

The percentage of positively (Spearman's rank correlation coefficient > 0.3) and negatively (Spearman's rank correlation coefficient < −0.3) co-expressed mRNAs in the gene sets.

GOBP_POSITIVE_REGULATION_OF_	Positive correlation	Negative correlation
EPITHELIAL_TO_MESENCHYMAL_TRANSITION	52.1 %	2.1 %
NON_CANONICAL_WNT_SIGNALING_PATHWAY	46.7 %	6.7 %
ACTIN_FILAMENT_DEPOLYMERIZATION	46.2 %	7.7 %
CELL_CELL_ADHESION_MEDIATED_BY_CADHERIN	45.5 %	9.1 %
TRANSFORMING_GROWTH_FACTOR_BETA_PRODUCTION	45.0 %	0.0 %
TRANSFORMING_GROWTH_FACTOR_BETA1_PRODUCTION	42.9 %	0.0 %
CELL_JUNCTION_ASSEMBLY	39.8 %	2.9 %
ACTIN_FILAMENT_BUNDLE_ASSEMBLY	39.3 %	0.0 %
SPROUTING_ANGIOGENESIS	38.1 %	0.0 %
CANONICAL_WNT_SIGNALING_PATHWAY	35.0 %	5.8 %
CELL_ADHESION_MEDIATED_BY_INTEGRIN	33.3 %	4.8 %
CELL_ADHESION	33.3 %	3.2 %
CYTOSKELETON_REORGANIZATION	33.0 %	5.2 %
EPITHELIAL_CELL_PROLIFERATION	32.6 %	4.9 %
CELL_CELL_ADHESION	29.9 %	3.8 %
ACTIN_FILAMENT_POLYMERIZATION	28.3 %	3.8 %
WNT_SIGNALING_PATHWAY	27.8 %	11.9 %
CELL_GROWTH	23.9 %	8.0 %
MESENCHYMAL_CELL_PROLIFERATION	23.8 %	0.0 %
CELL_DEATH	21.2 %	6.8 %
APOPTOTIC_SIGNALING_PATHWAY	19.8 %	8.7 %
ACTIN_NUCLEATION	18.8 %	6.3 %
INTRACELLULAR_STEROID_HORMONE_RECEPTOR_SIGNALING_PATHWAY	16.7 %	25.0 %
ANOIKIS	0.0 %	28.6 %

GOBP_NEGATIVE_REGULATION_OF_	Positive correlation	Negative correlation
ACTIN_NUCLEATION	0.0 %	11.1 %
CELL_CELL_ADHESION_MEDIATED_BY_CADHERIN	20.0 %	10.0 %
APOPTOTIC_SIGNALING_PATHWAY	19.5 %	6.3 %
CELL_DEATH	22.0 %	6.2 %
ANOIKIS	52.9 %	5.9 %
CYTOSKELETON_ORGANIZATION	30.5 %	5.8 %
EPITHELIAL_TO_MESENCHYMAL_TRANSITION	42.9 %	5.7 %
INTRACELLULAR_STEROID_HORMONE_RECEPTOR_SIGNALING_PATHWAY	25.7 %	5.7 %
EPITHELIAL_CELL_PROLIFERATION	34.4 %	4.9 %
CELL_ADHESION	32.5 %	3.9 %
CELL_GROWTH	26.9 %	3.8 %
CELL_CELL_ADHESION	26.6 %	3.5 %
ACTIN_FILAMENT_BUNDLE_ASSEMBLY	38.7 %	3.2 %
CELL_JUNCTION_ASSEMBLY	46.9 %	3.1 %
WNT_SIGNALING_PATHWAY	36.4 %	3.0 %
ACTIN_FILAMENT_DEPOLYMERIZATION	39.5 %	2.3 %
CANONICAL_WNT_SIGNALING_PATHWAY	38.6 %	2.3 %
ACTIN_FILAMENT_POLYMERIZATION	32.8 %	1.6 %
NON_CANONICAL_WNT_SIGNALING_PATHWAY	60.0 %	0.0 %
CELL_ADHESION_MEDIATED_BY_INTEGRIN	33.3 %	0.0 %
MESENCHYMAL_CELL_PROLIFERATION	33.3 %	0.0 %
TRANSFORMING_GROWTH_FACTOR_BETA_PRODUCTION	30.8 %	0.0 %
SPROUTING_ANGIOGENESIS	27.3 %	0.0 %
TRANSFORMING_GROWTH_FACTOR_BETA1_PRODUCTION	14.3 %	0.0 %

Supplementary Table 2. RNA sequencing analysis of the castration-resistant VCaP xenograft tumors in nude mice treated with enzalutamide.

Table showing the mRNA expressions of markers for epithelial phenotype (*CDH1* and *KRT8*) and markers for mesenchymal phenotype (*VIM*, *CDH2*, *CDH11*, *ZEB1* and *ZEB2*). Serum PSA values are also presented.

Tumor_ID	Group	PSA	*VIM*	*CDH1*	*CDH2*	*CDH11*	*ZEB1*	*ZEB2*	*KRT8*
TU_15	Response	2.81	0.00	308.25	0.00	0.00	0.00	0.00	94.70
TU_16	Response	1.42	0.07	314.83	0.00	0.00	0.00	0.00	179.62
TU_31	Response	4.91	0.00	266.93	0.14	0.00	0.00	0.00	100.63
TU_63	Response	3.98	0.00	279.73	0.08	0.00	0.00	0.00	95.84
TU_78	Response	4.50	0.00	390.27	0.23	0.00	0.68	0.00	109.40
TU_113	Response	5.48	0.00	358.91	0.00	0.00	0.25	0.00	185.69
TU_114	Response	8.49	0.00	243.41	0.36	0.07	0.44	0.00	120.15
TU_115	Response	1.42	0.00	318.10	0.28	0.00	0.21	0.00	106.43
TU_117	Response	0.94	0.00	344.64	0.00	0.00	0.08	0.00	115.77
TU_131	Response	2.76	0.00	341.61	0.11	0.00	0.22	0.00	120.33
TU_19	Resistance	11.53	0.00	384.39	0.17	0.00	0.00	0.00	127.12
TU_45	Resistance	8.33	0.00	275.33	0.18	0.00	0.00	0.00	126.85
TU_55	Resistance	26.94	0.00	331.24	0.00	0.00	0.20	0.00	105.27
TU_73	Resistance	38.24	0.00	380.48	0.71	0.00	0.10	0.00	98.83
TU_75	Resistance	4.87	0.00	394.25	0.00	0.00	0.15	0.00	117.81
TU_86	Resistance	14.82	0.00	308.73	0.11	0.00	0.00	0.00	130.30
TU_120	Resistance	15.64	0.00	420.23	0.00	0.00	0.22	0.00	145.03
TU_135	Resistance	19.81	0.00	323.45	0.19	0.00	0.37	0.00	96.16
TU_140	Resistance	23.19	0.00	388.41	0.22	0.00	0.11	0.11	91.27
TU_141	Resistance	38.39	0.00	343.86	0.11	0.00	0.11	0.00	128.06
