# Supplementary material for: Glucocorticoid receptor-induced non-muscle caldesmon regulates metastasis in castration-resistant prostate cancer
Source: Oncogenesis. 2023 Aug 12;12(1):42. doi: 10.1038/s41389-023-00485-z (PMC10423232; doi:10.1038/s41389-023-00485-z)

# Fig. 1A raw data for Western blot using anti-l-CaD antibody

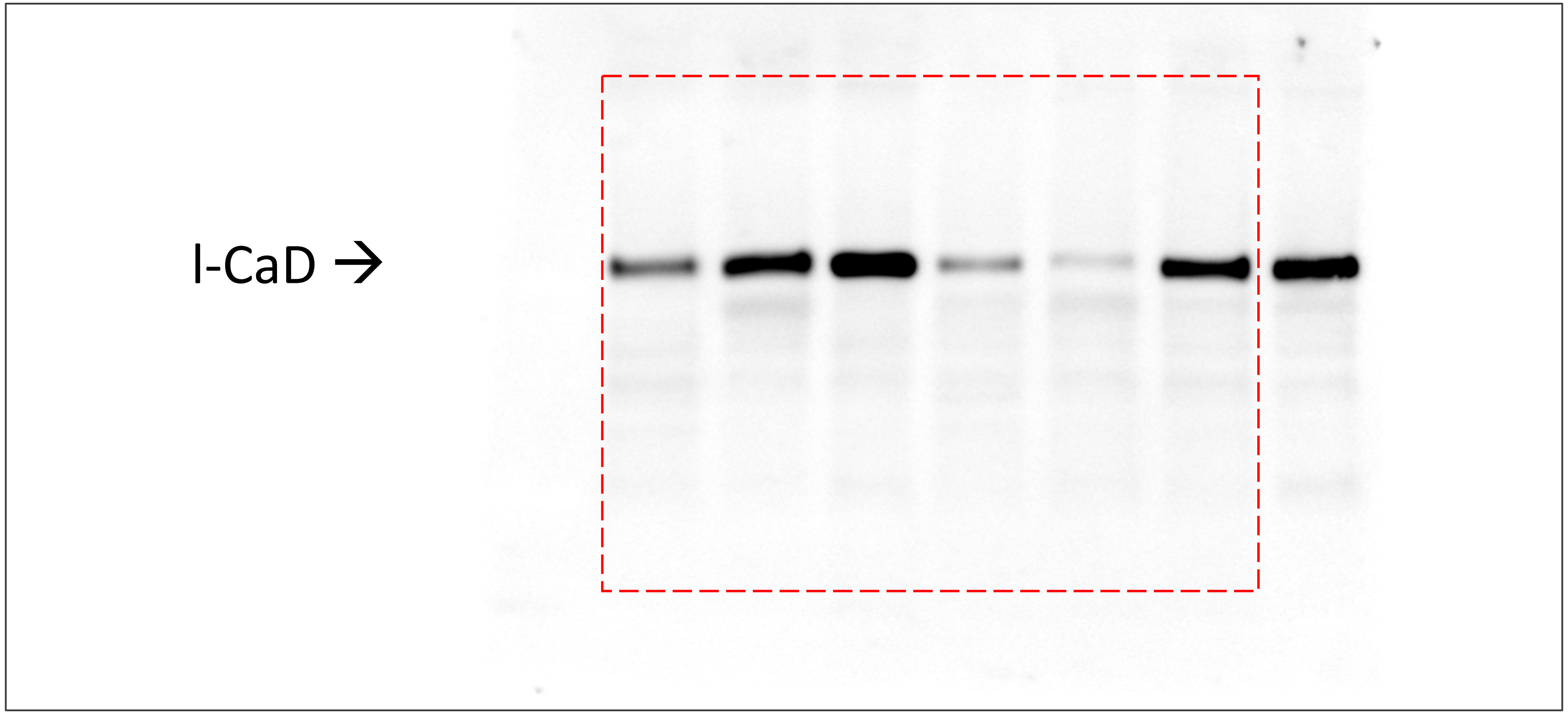

# Fig. 1A raw data for Western blot using anti-actin antibody

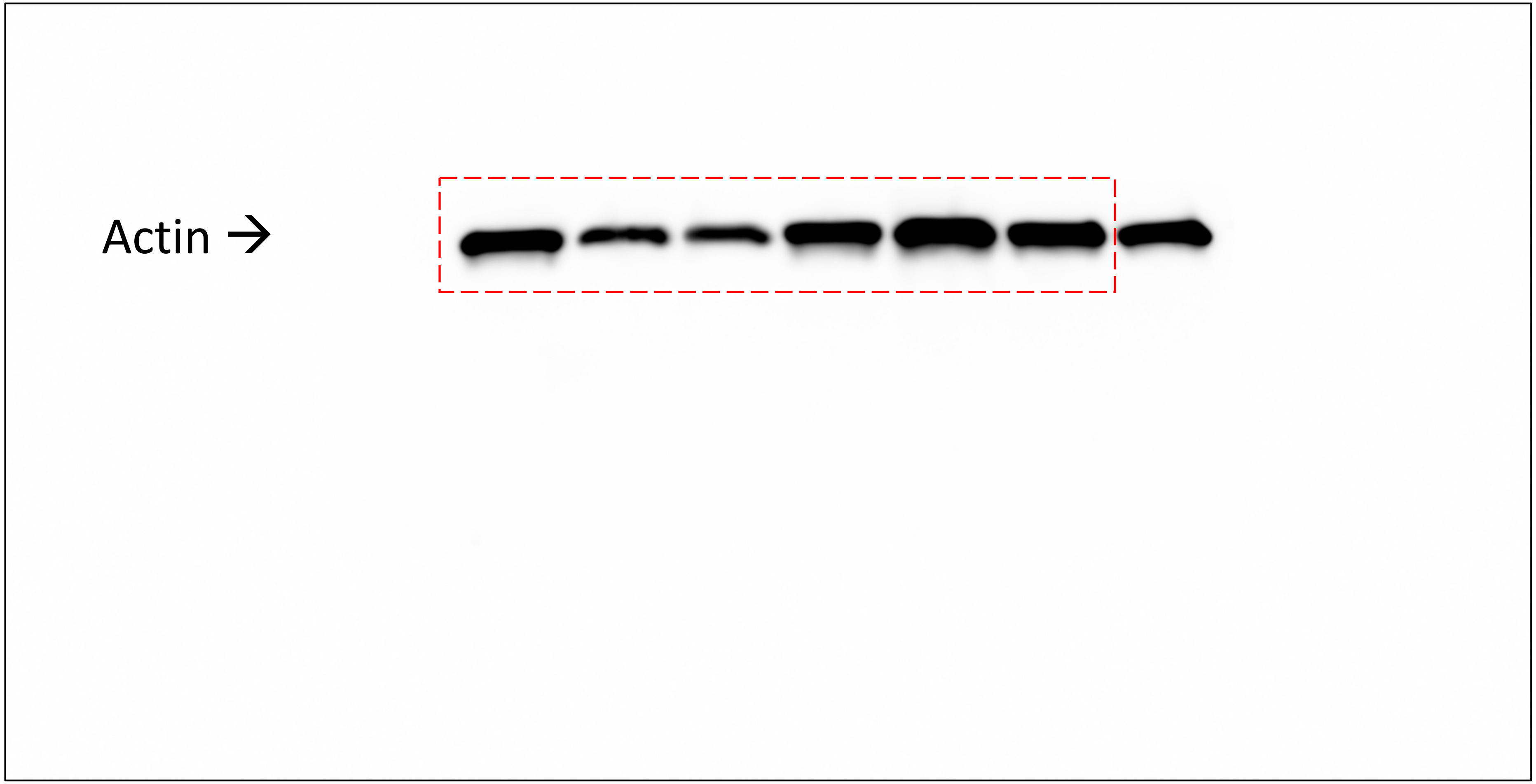

# Fig. 1D raw data for Western blot using anti-l-CaD antibody (exp 1/3)

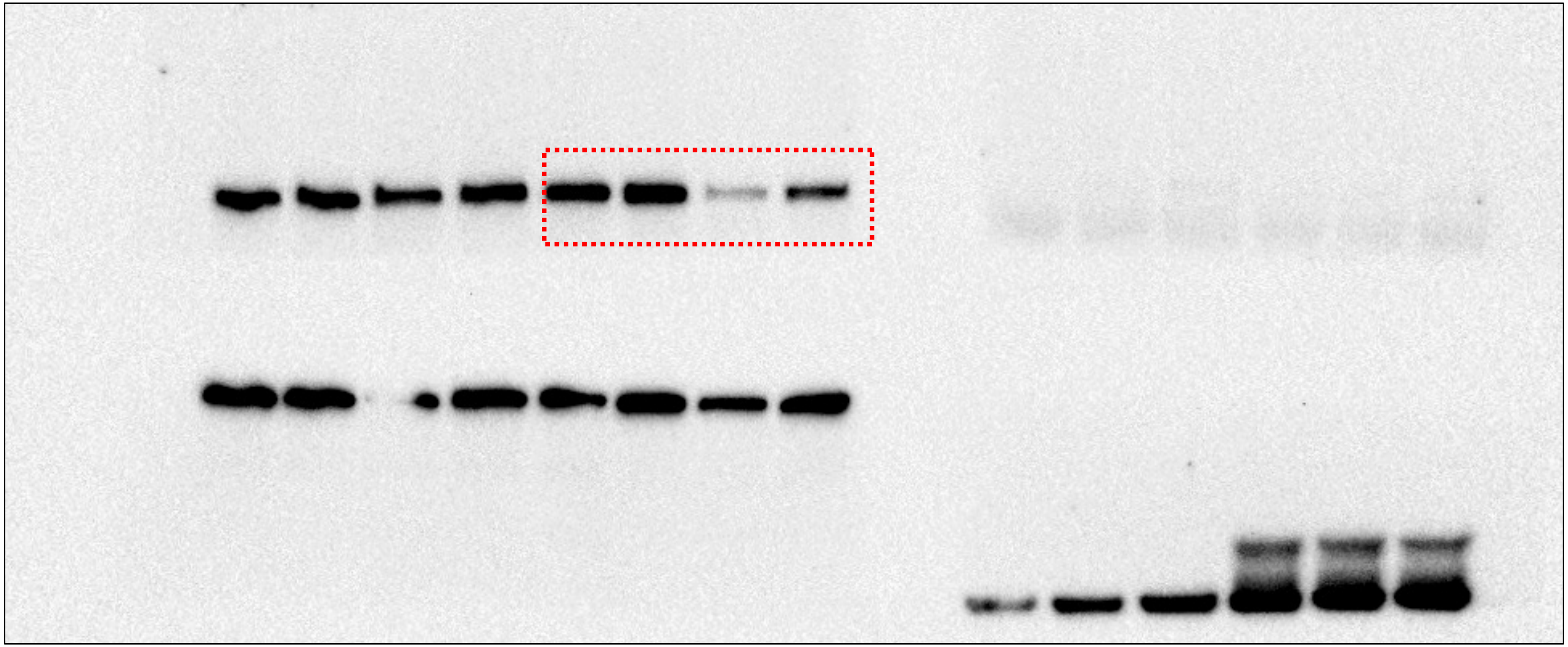

# Fig. 1D raw data for Western blot using anti-actin antibody (exp 1/3)

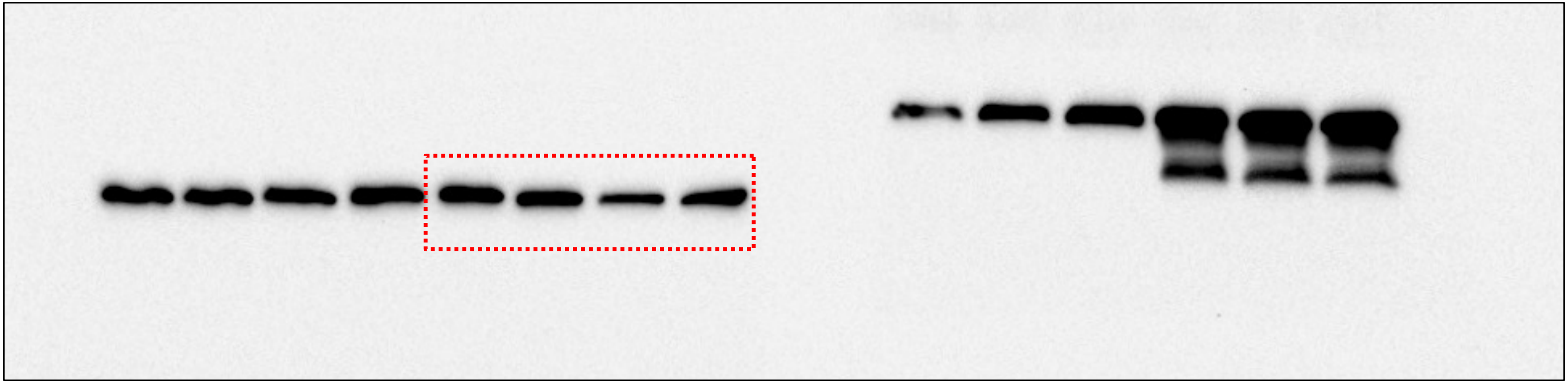

# Fig. 1D raw data for Western blot using anti-l-CaD antibody (exp 2/3)

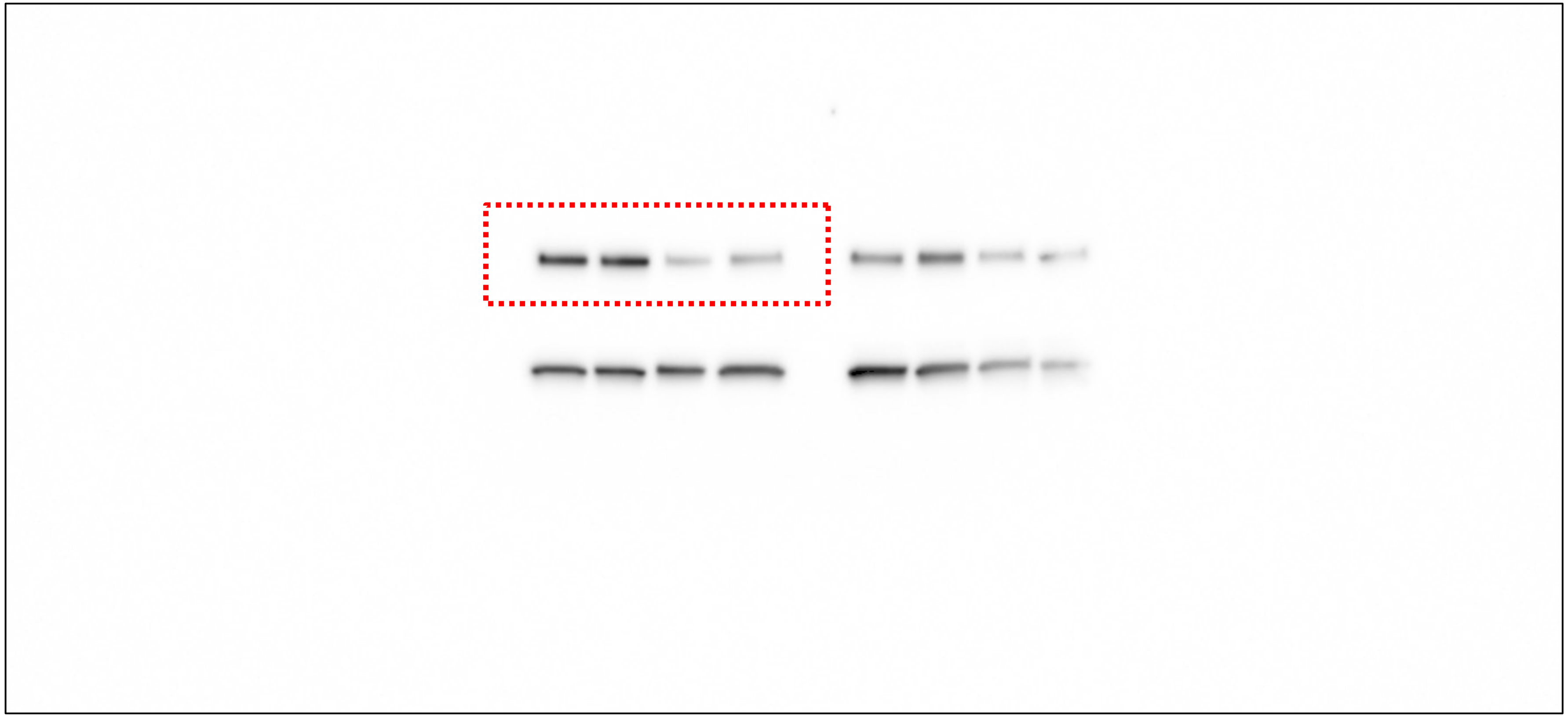

# Fig. 1D raw data for Western blot using anti-actin antibody (exp 2/3)

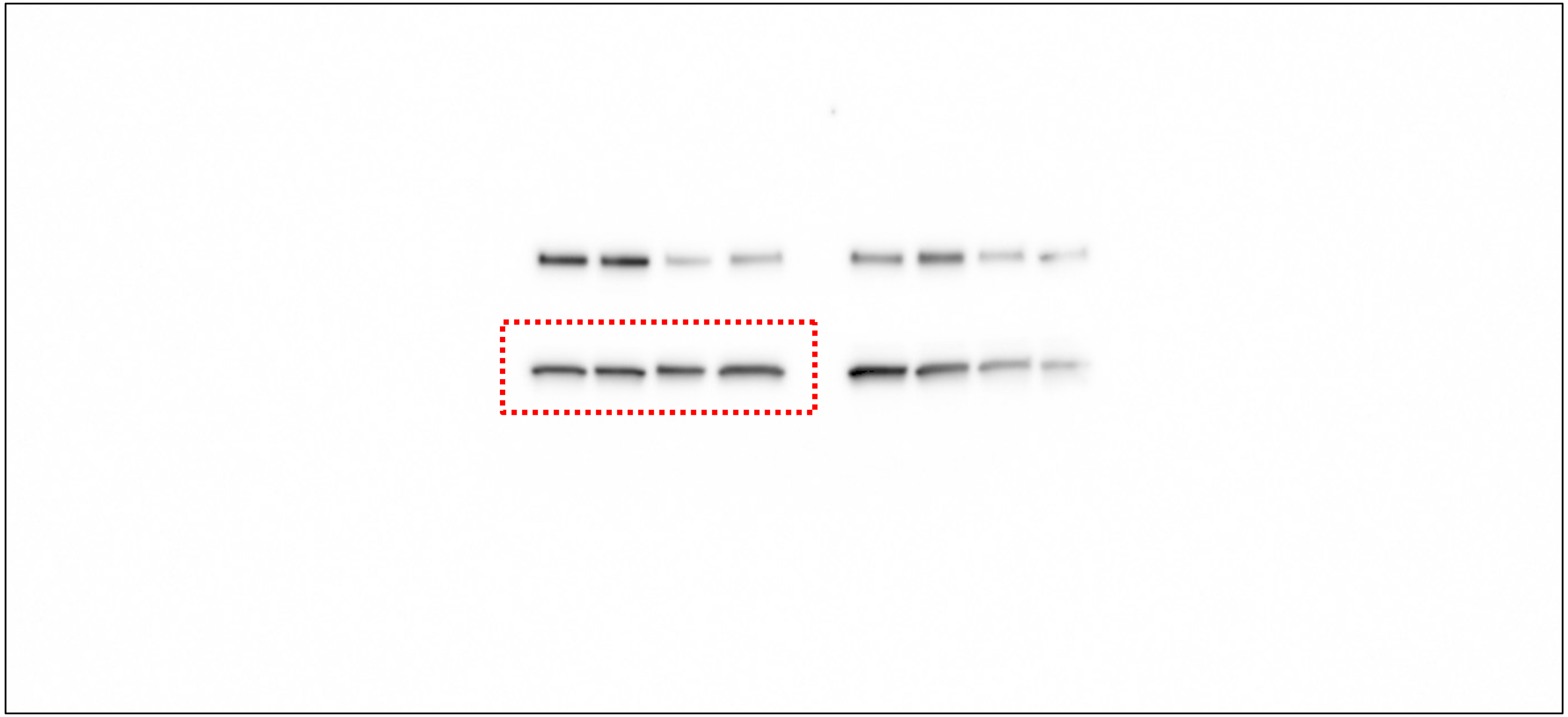

# Fig. 1D raw data for Western blot using anti-l-CaD antibody (exp 3/3)

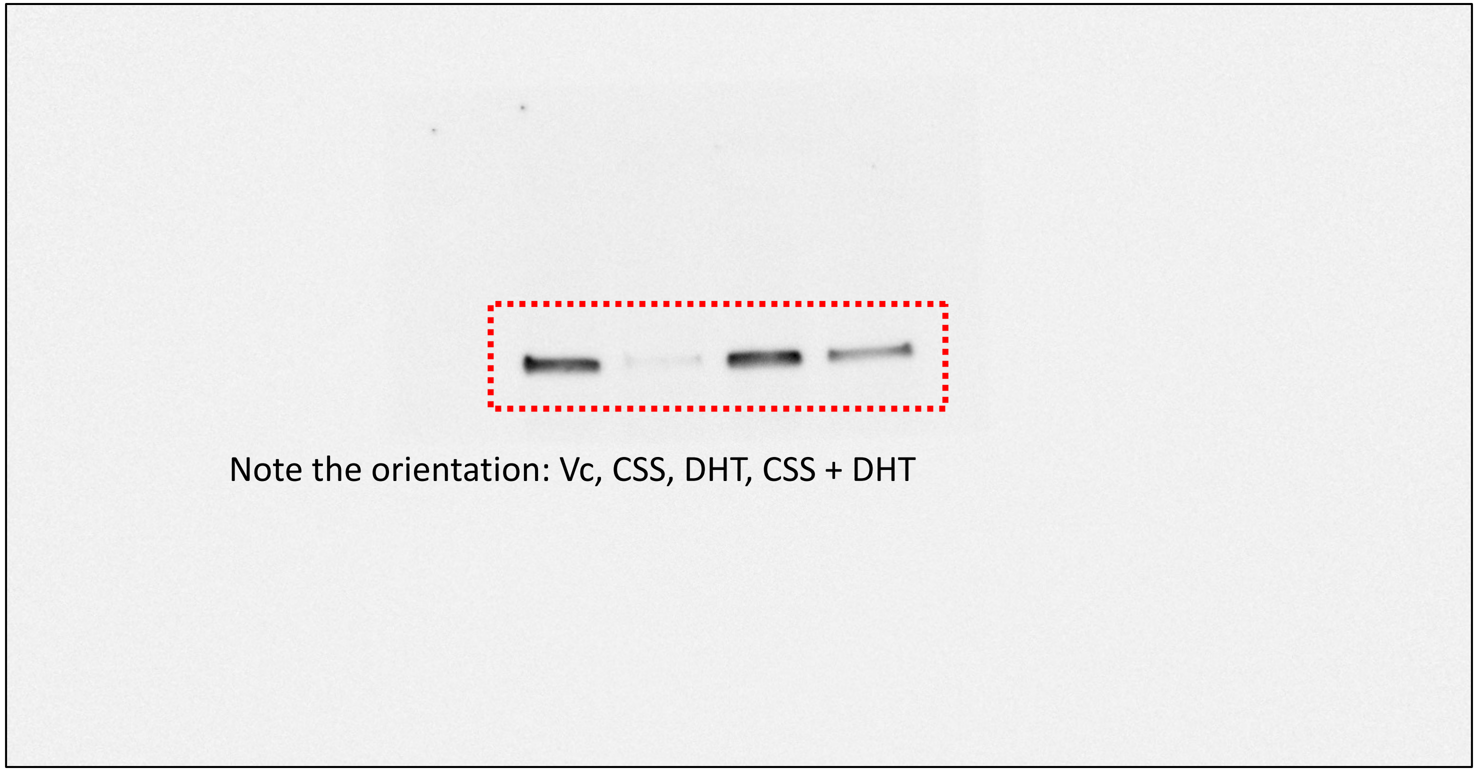

# Fig. 1D raw data for Western blot using anti-actin antibody (exp 3/3)

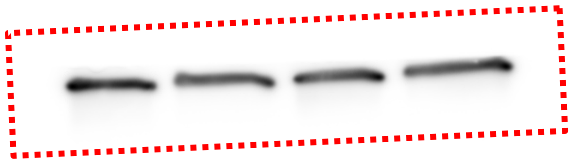

Note the orientation: Vc, CSS, DHT, CSS + DHT

# Fig. 2F raw data for Western blot using anti-l-CaD antibody

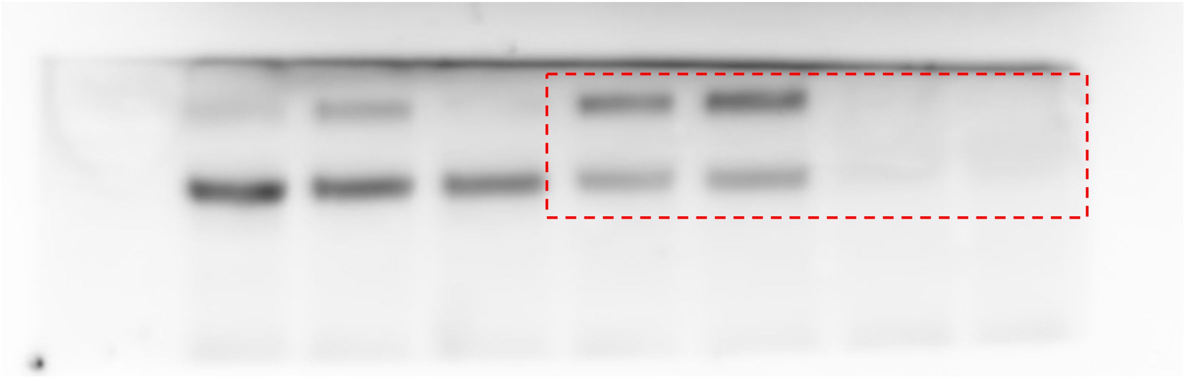

# Fig. 2F raw data for Western blot using anti-vinculin antibody

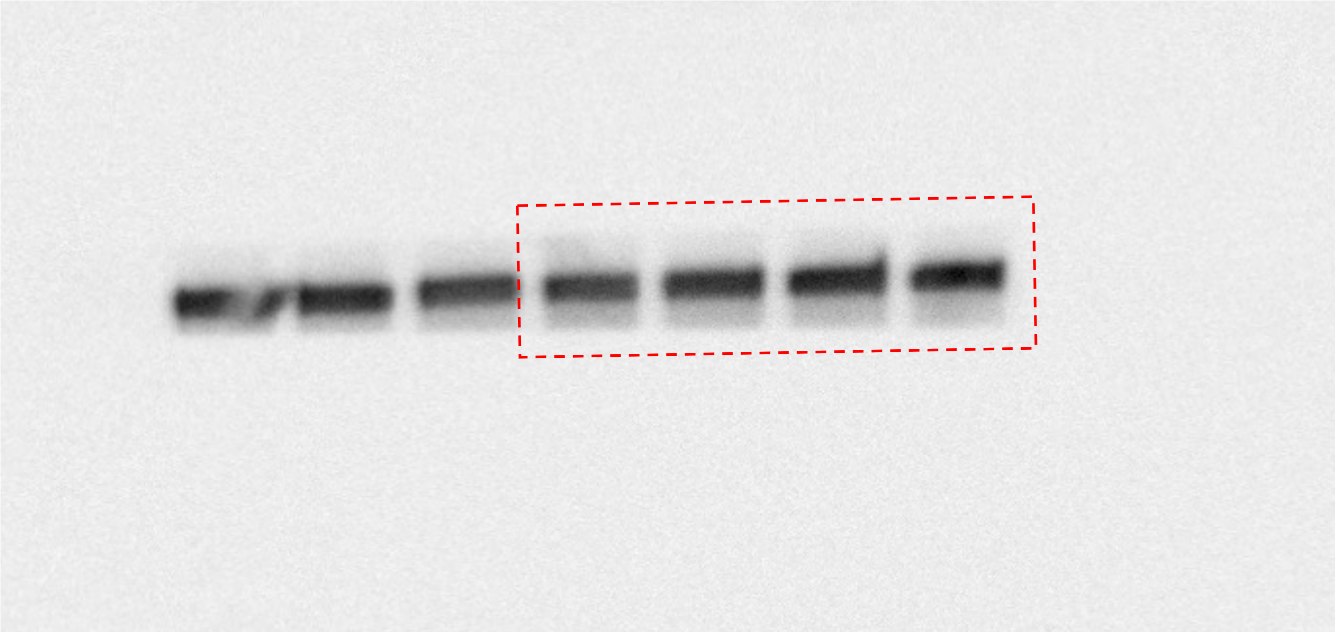

# Fig. 2J raw data for Western blot using anti-l-CaD antibody

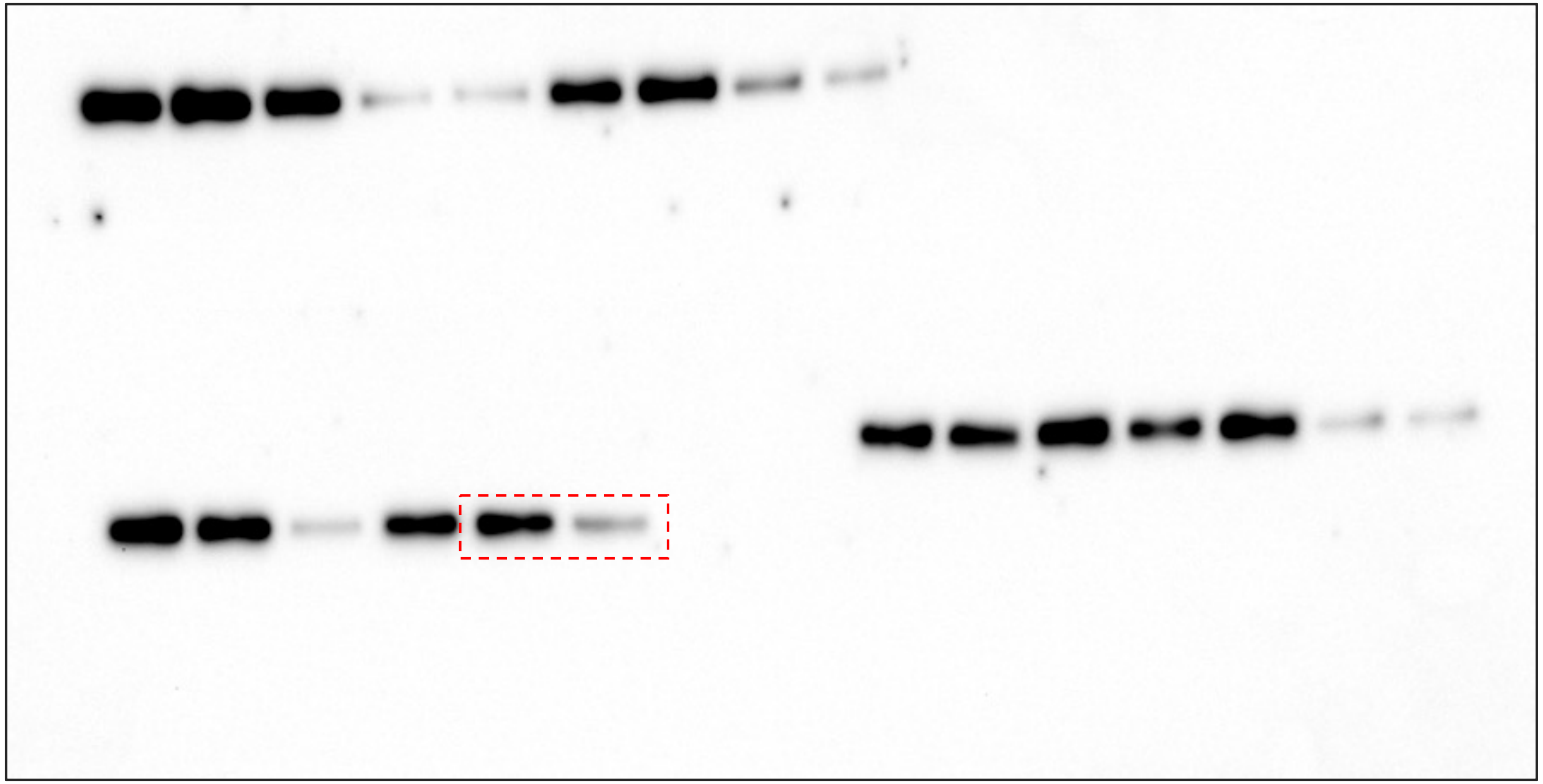

# Fig. 2J raw data for Western blot using anti-vinculin antibody

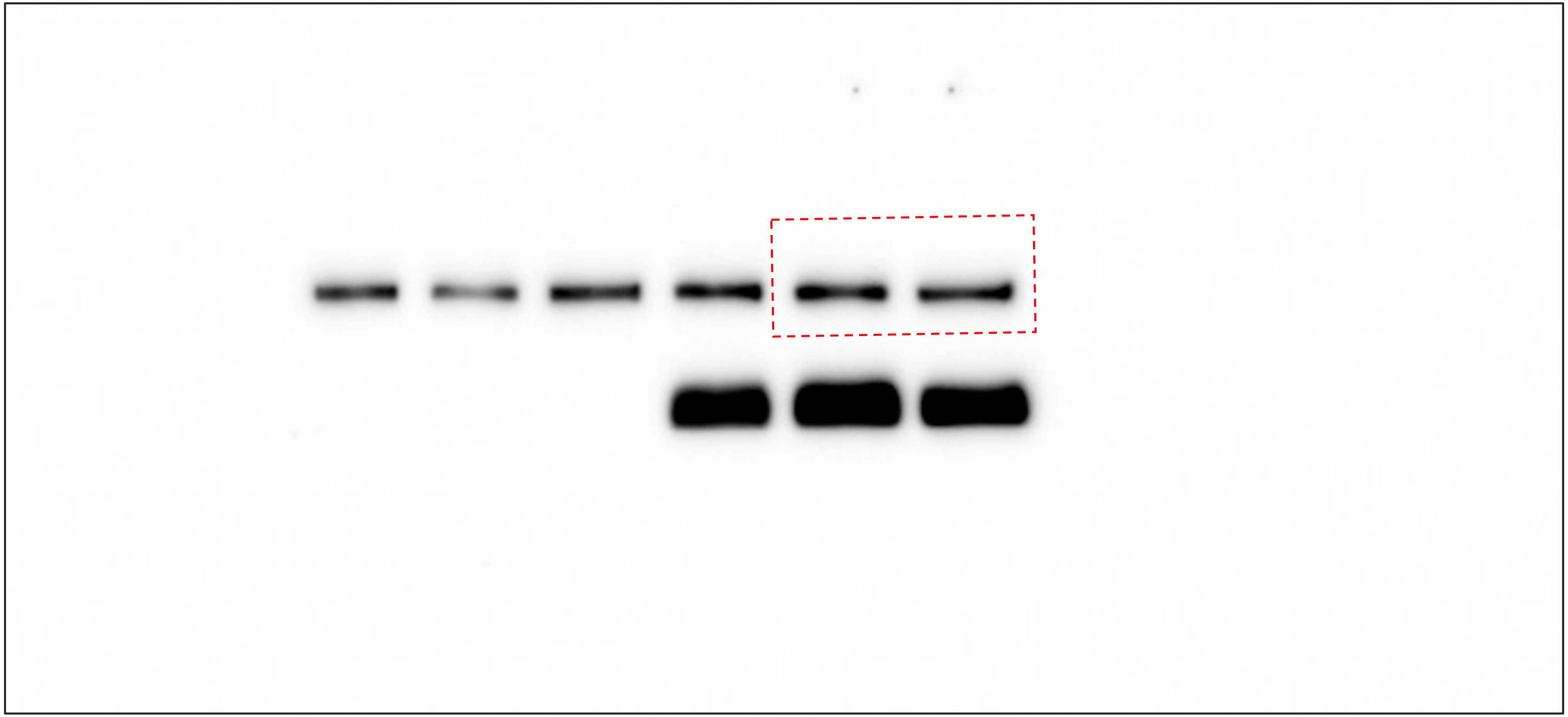

# Fig. 3B raw data for Western blot using anti-GR antibody

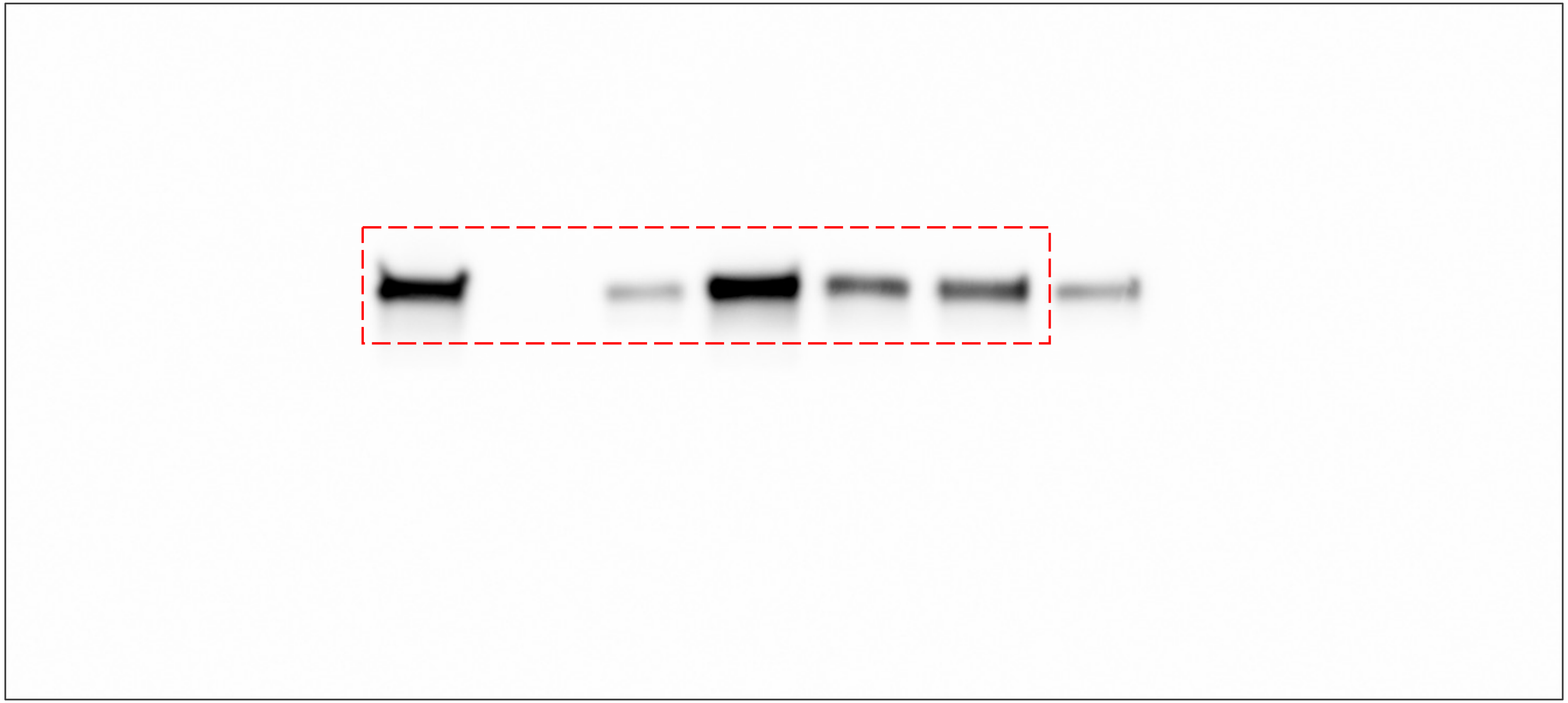

# Fig. 3B raw data for Western blot using anti-actin antibody

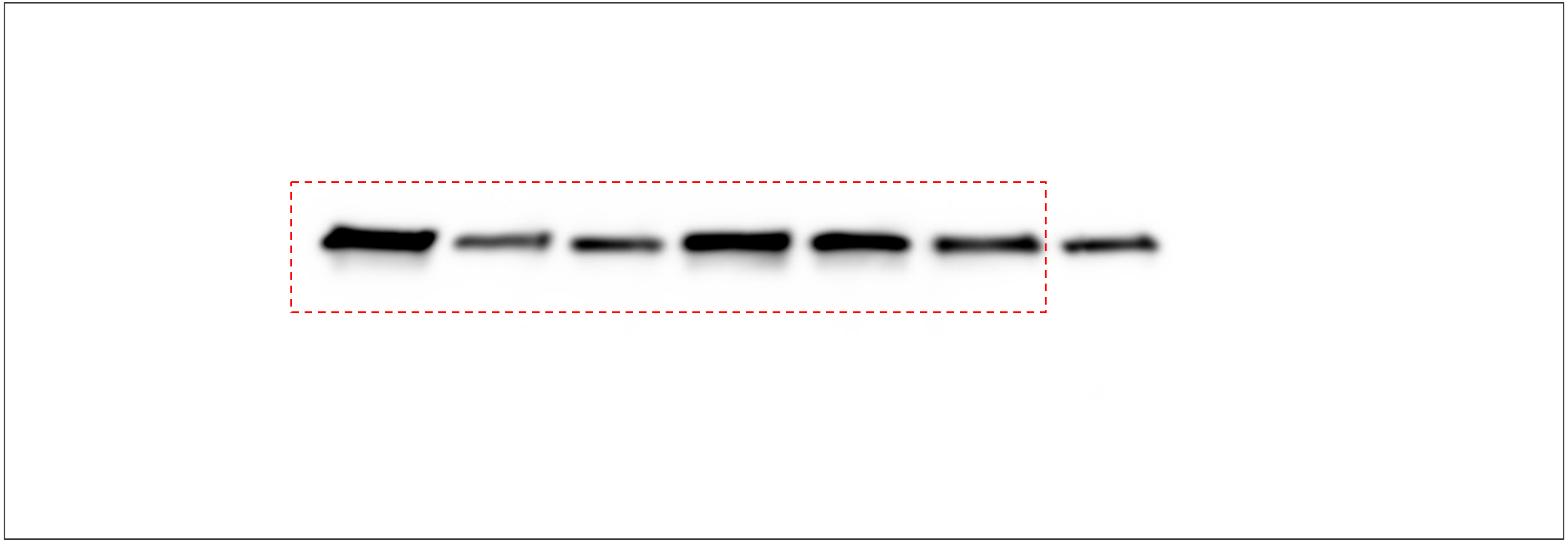

Fig. 3D raw data for Western blot using anti-l-CaD antibody (exp 1/3)

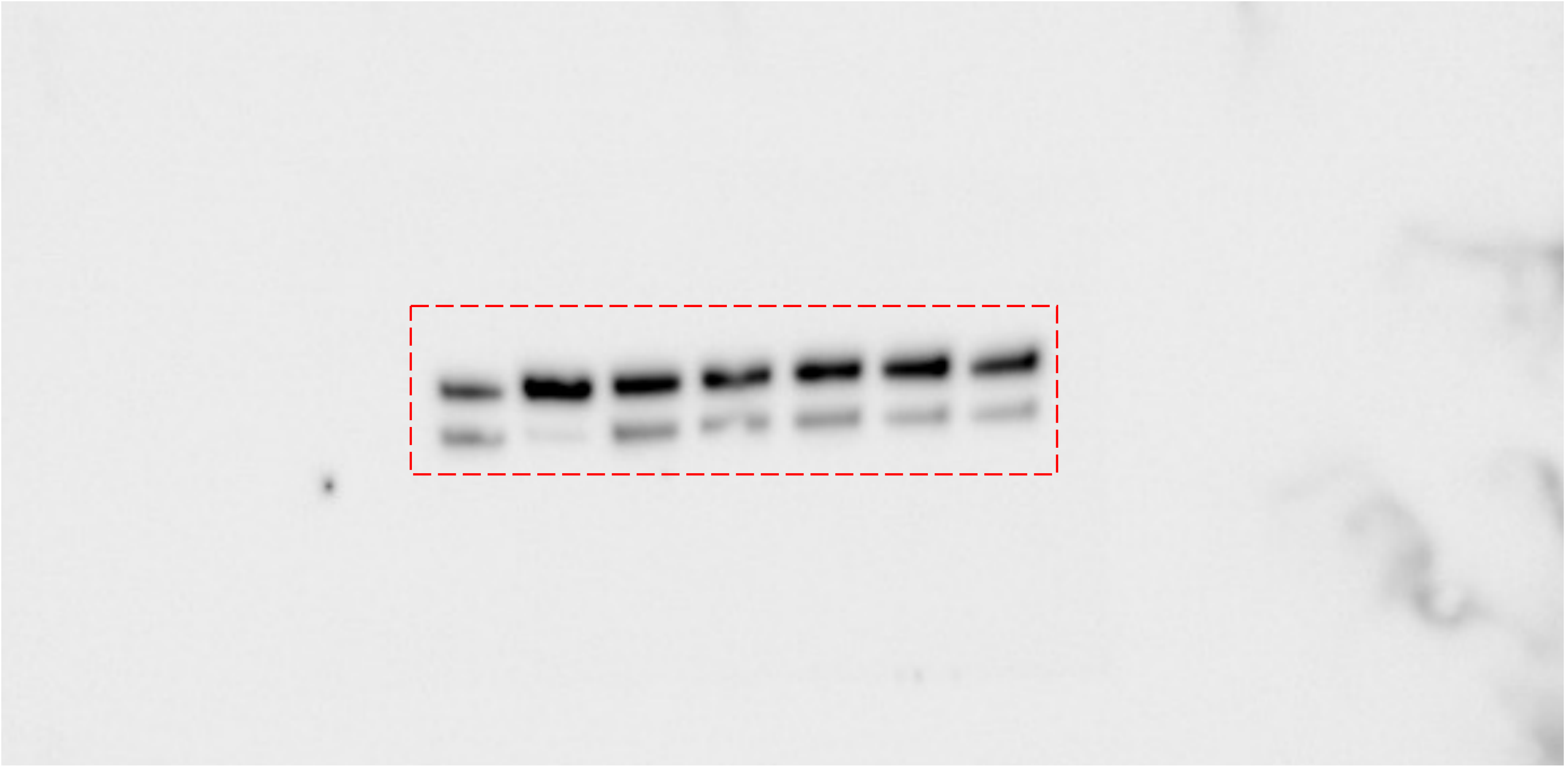

# Fig. 3D raw data for Western blot using anti-vinculin antibody (exp 1/3)

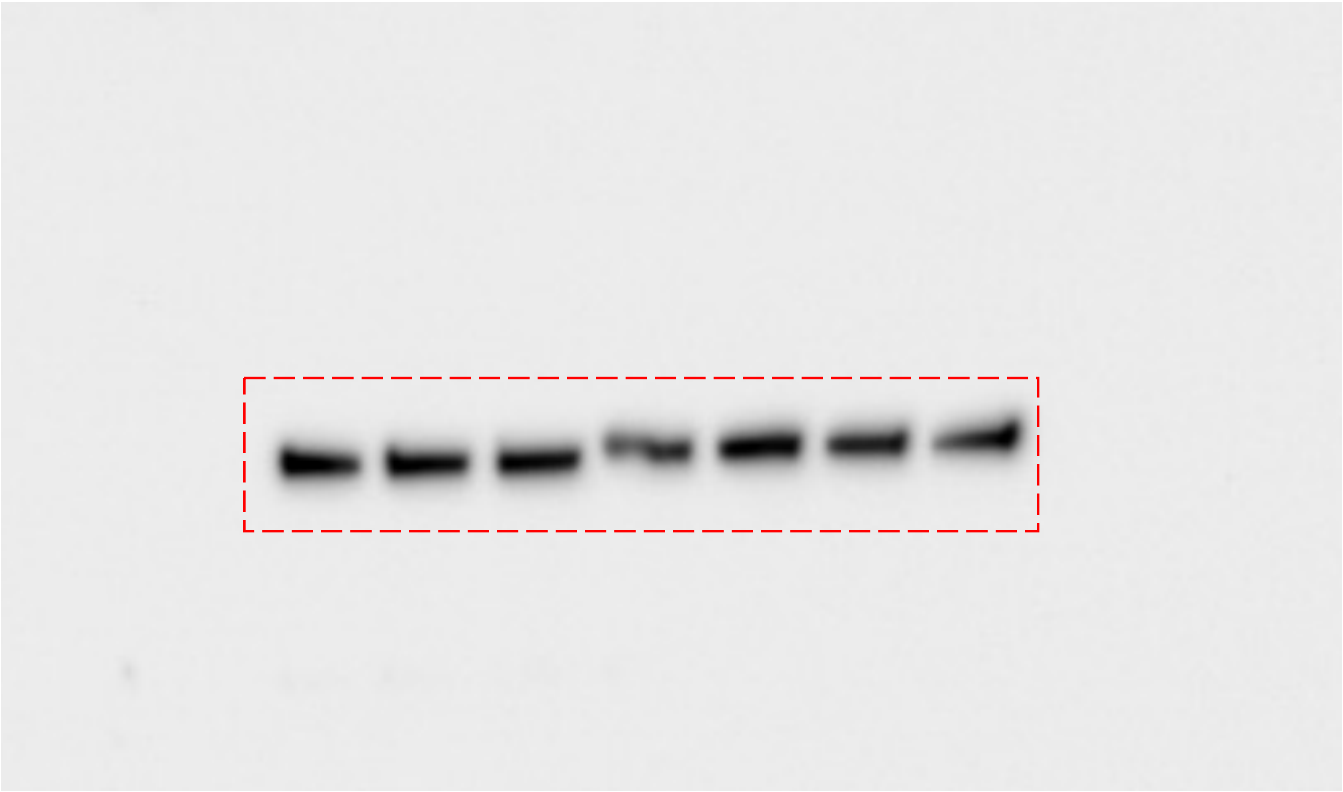

# Fig. 3D raw data for Western blot using anti-l-CaD antibody (exp 2/3)

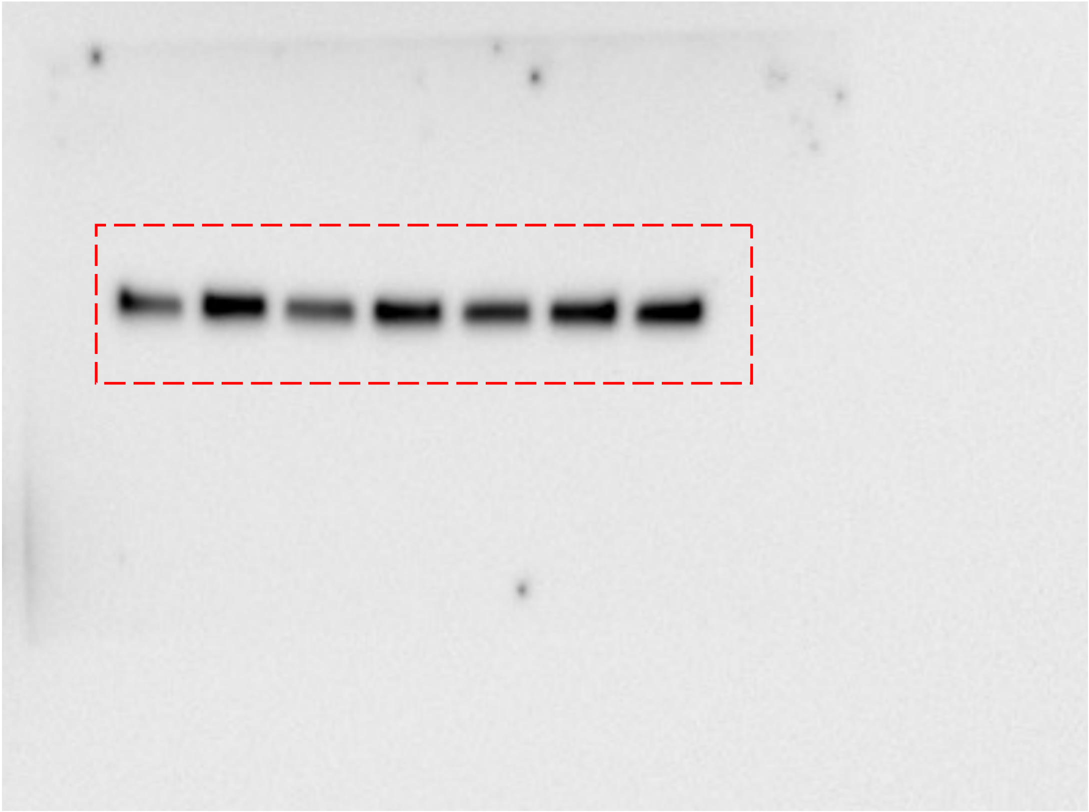

# Fig. 3D raw data for Western blot using anti-actin antibody (exp 2/3)

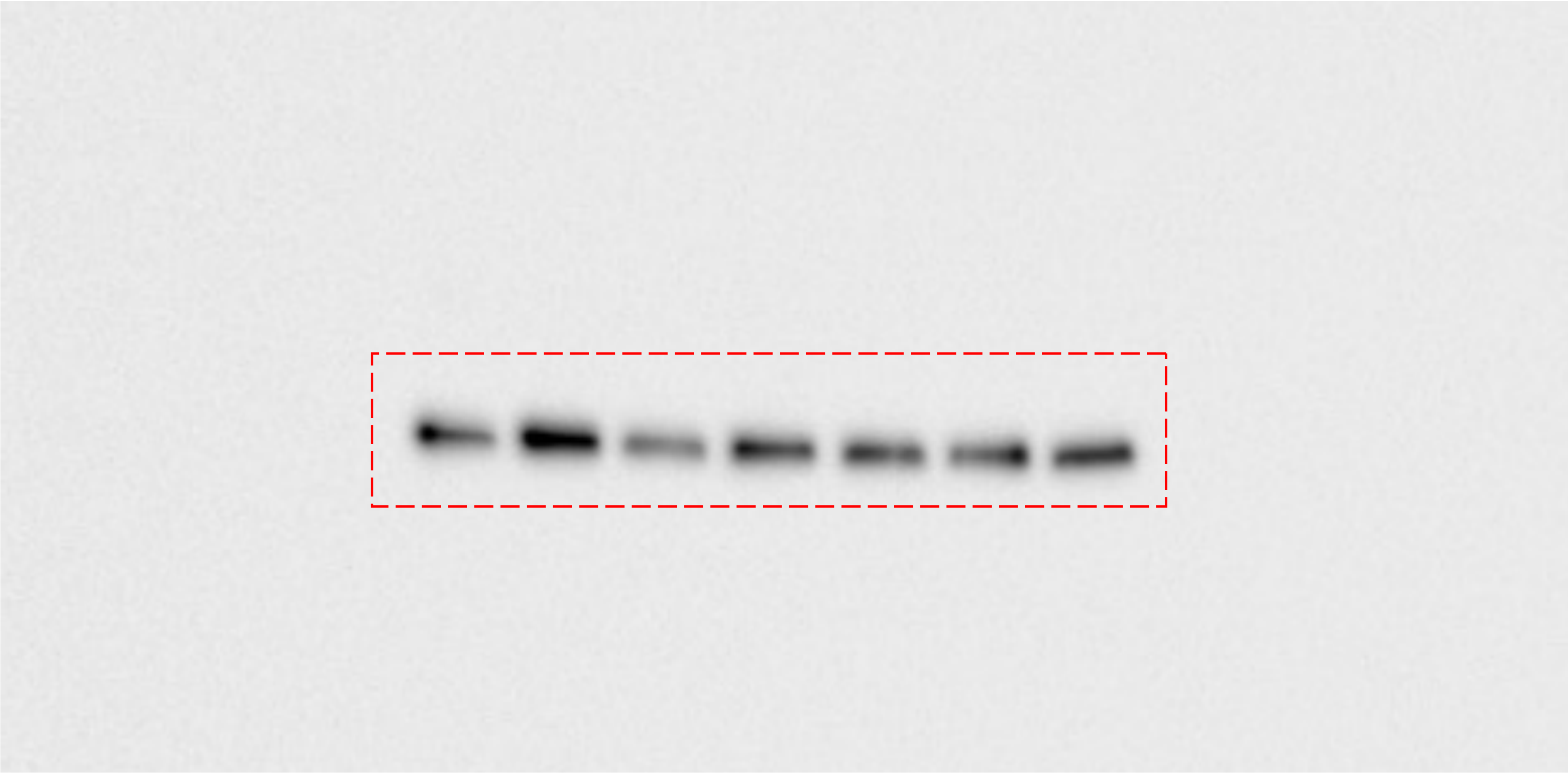

# Fig. 3D raw data for Western blot using anti-l-CaD antibody (exp 3/3)

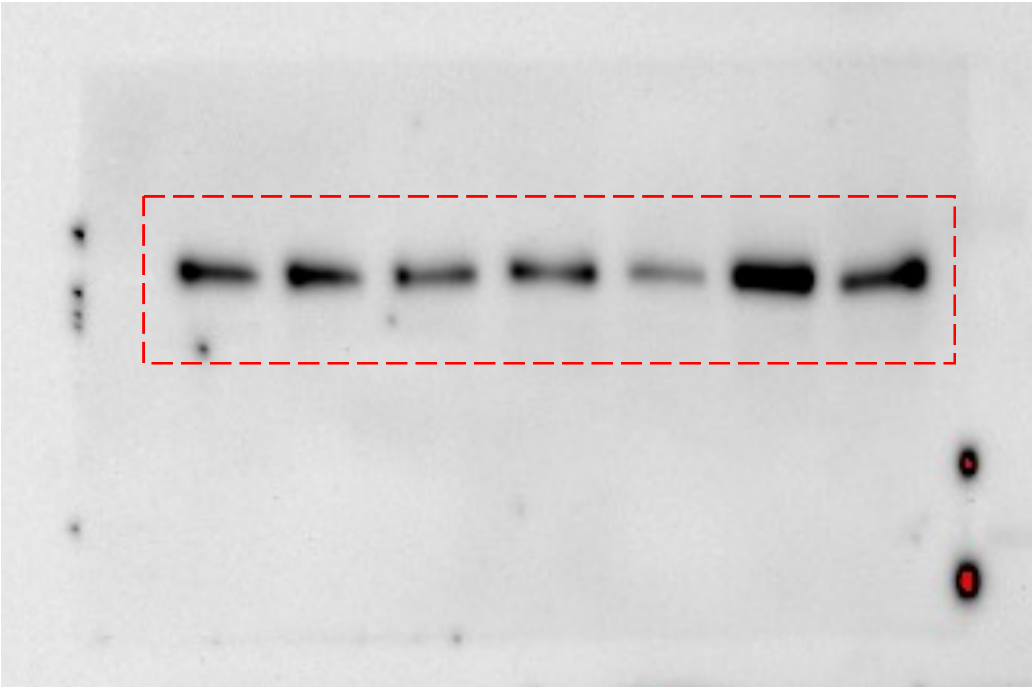

# Fig. 3D raw data for Western blot using anti-actin antibody (exp 3/3)

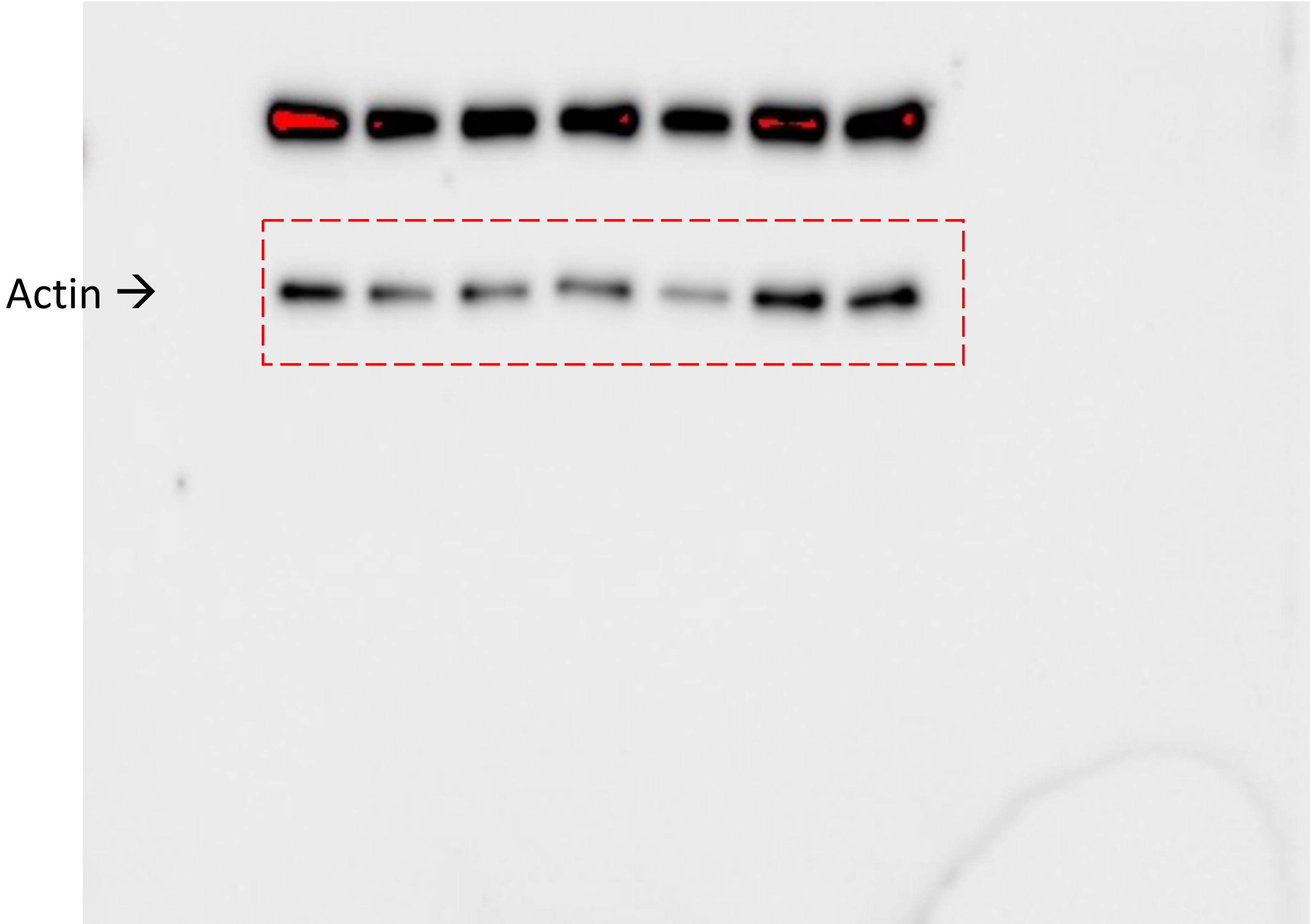

# Fig. 3E raw data for Western blot using anti-l-CaD antibody (exp 1/3)

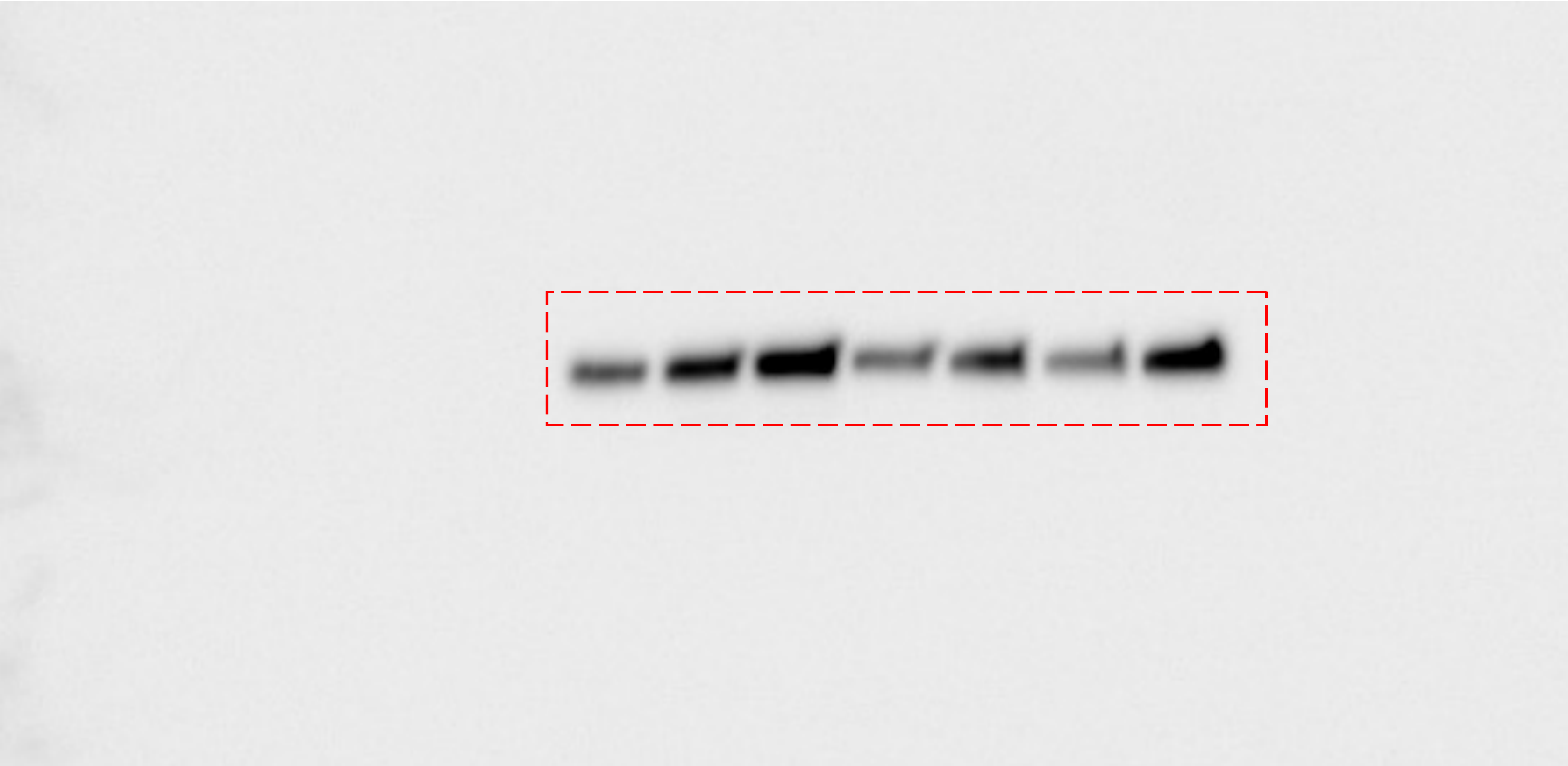

# Fig. 3E raw data for Western blot using anti-actin antibody (exp 1/3)

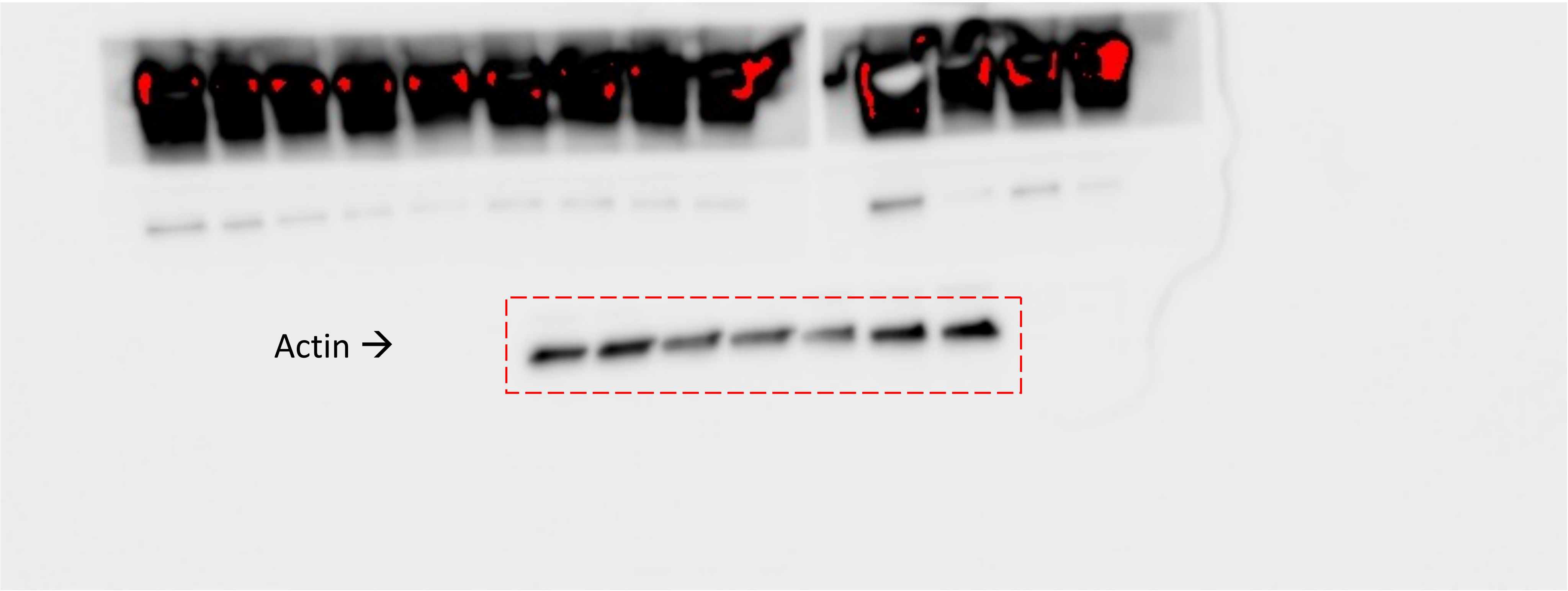

# Fig. 3E raw data for Western blot using anti-l-CaD antibody (exp 2/3)

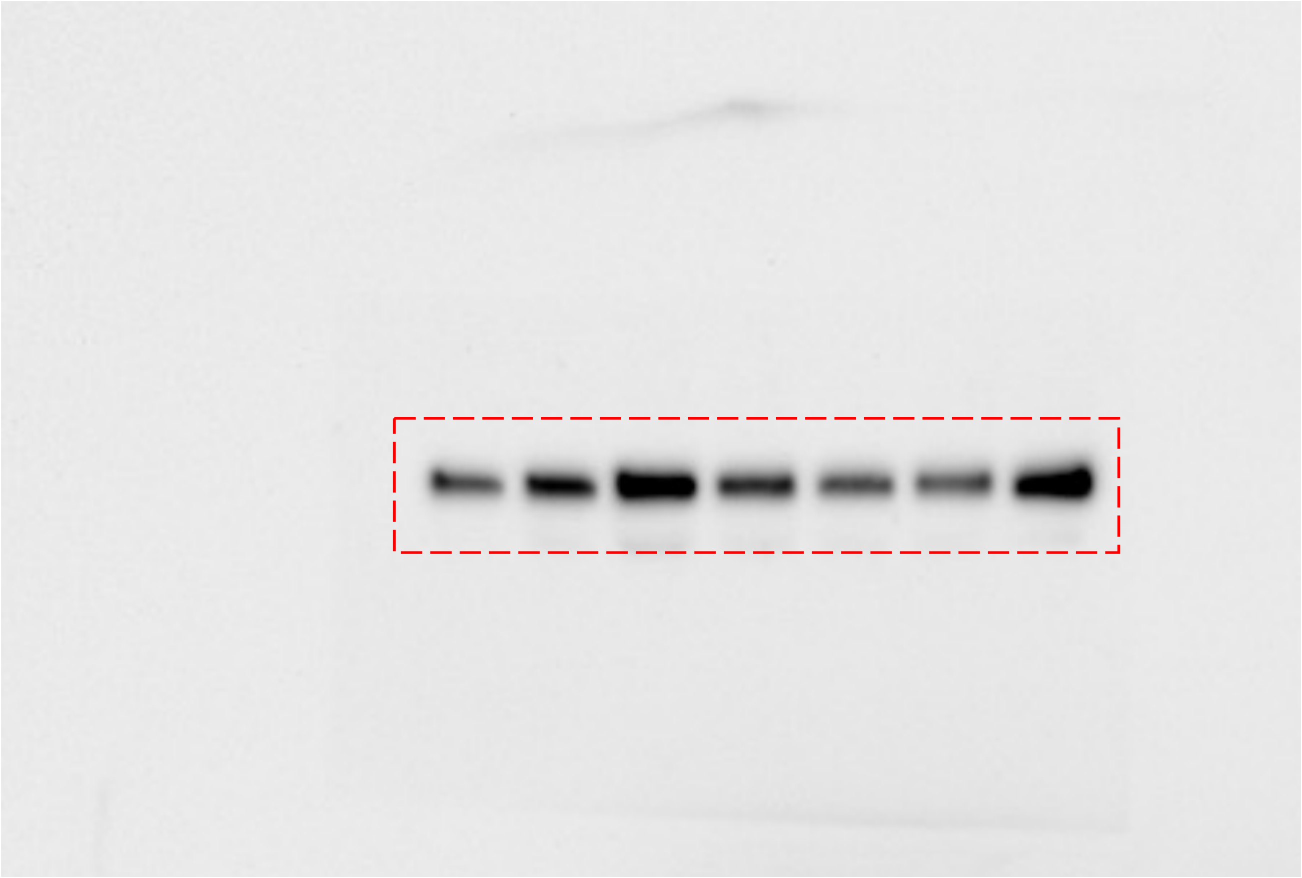

# Fig. 3E raw data for Western blot using anti-actin antibody (exp 2/3)

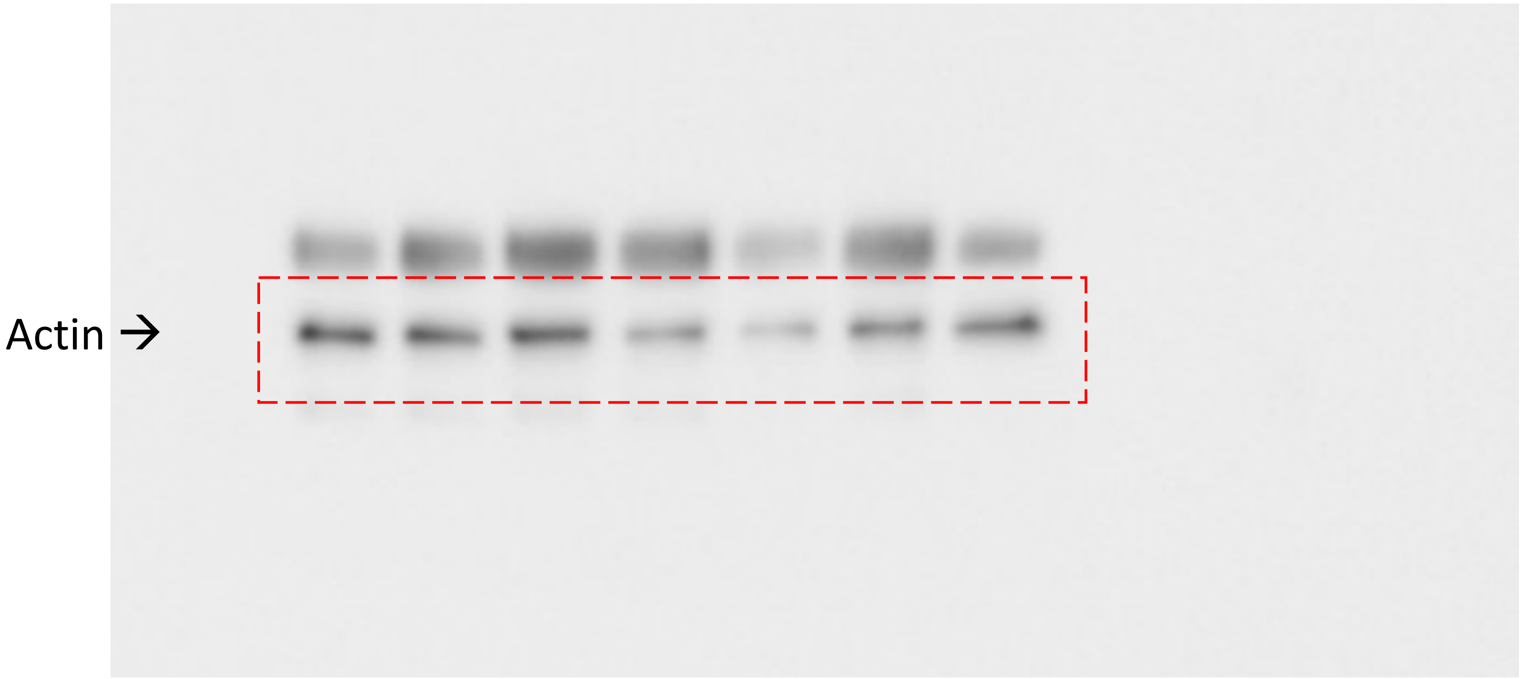

# Fig. 3E raw data for Western blot using anti-l-CaD antibody (exp 3/3)

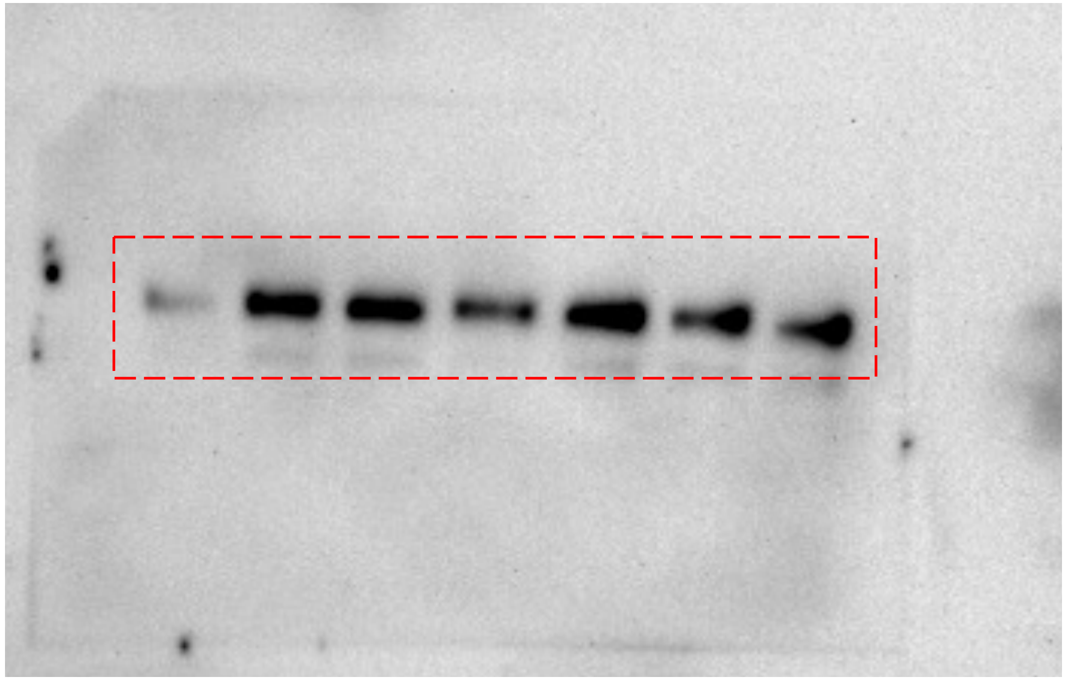

# Fig. 3E raw data for Western blot using anti-actin antibody (exp 3/3)

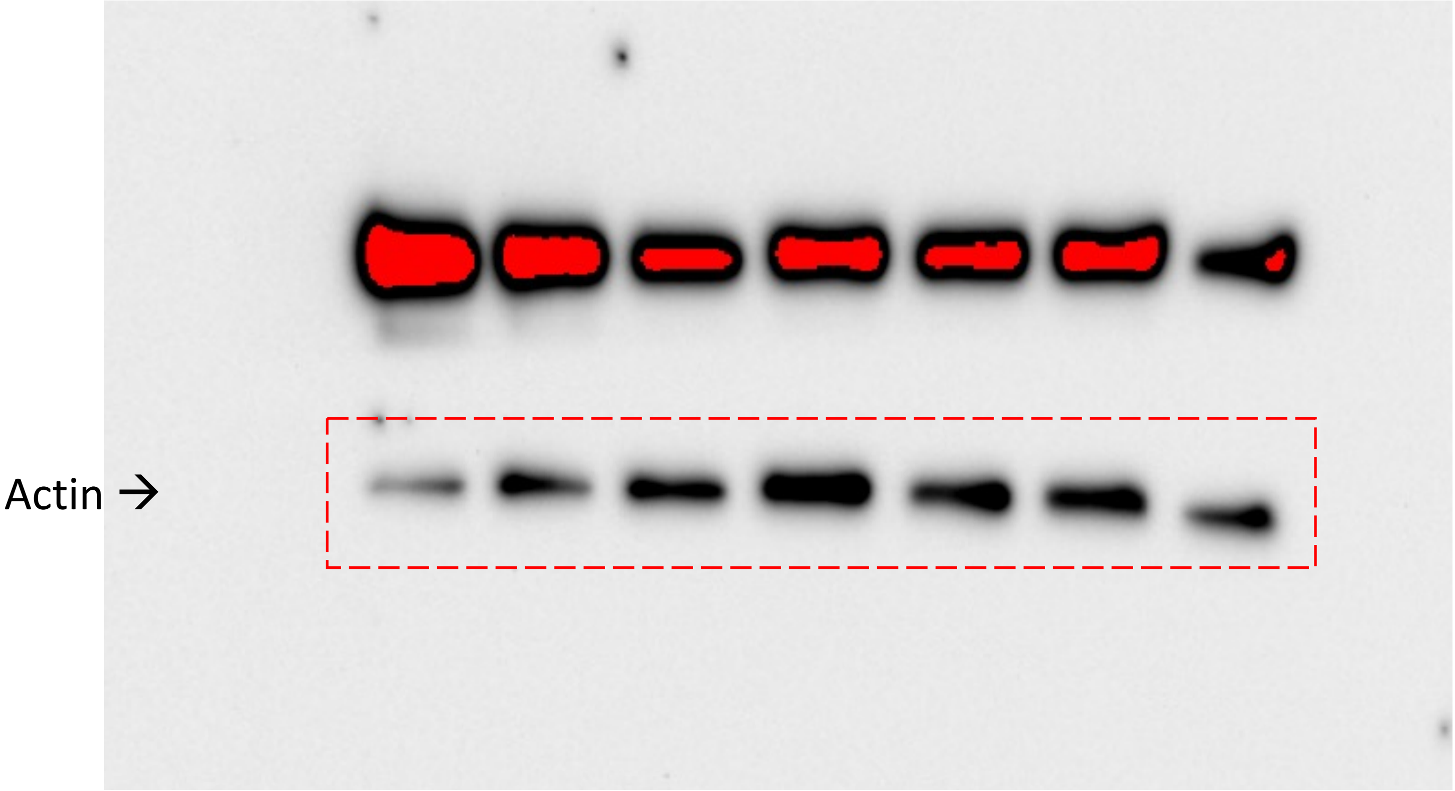

# Fig. 3F raw data for Western blot using anti-l-CaD antibody (exp 1/3)

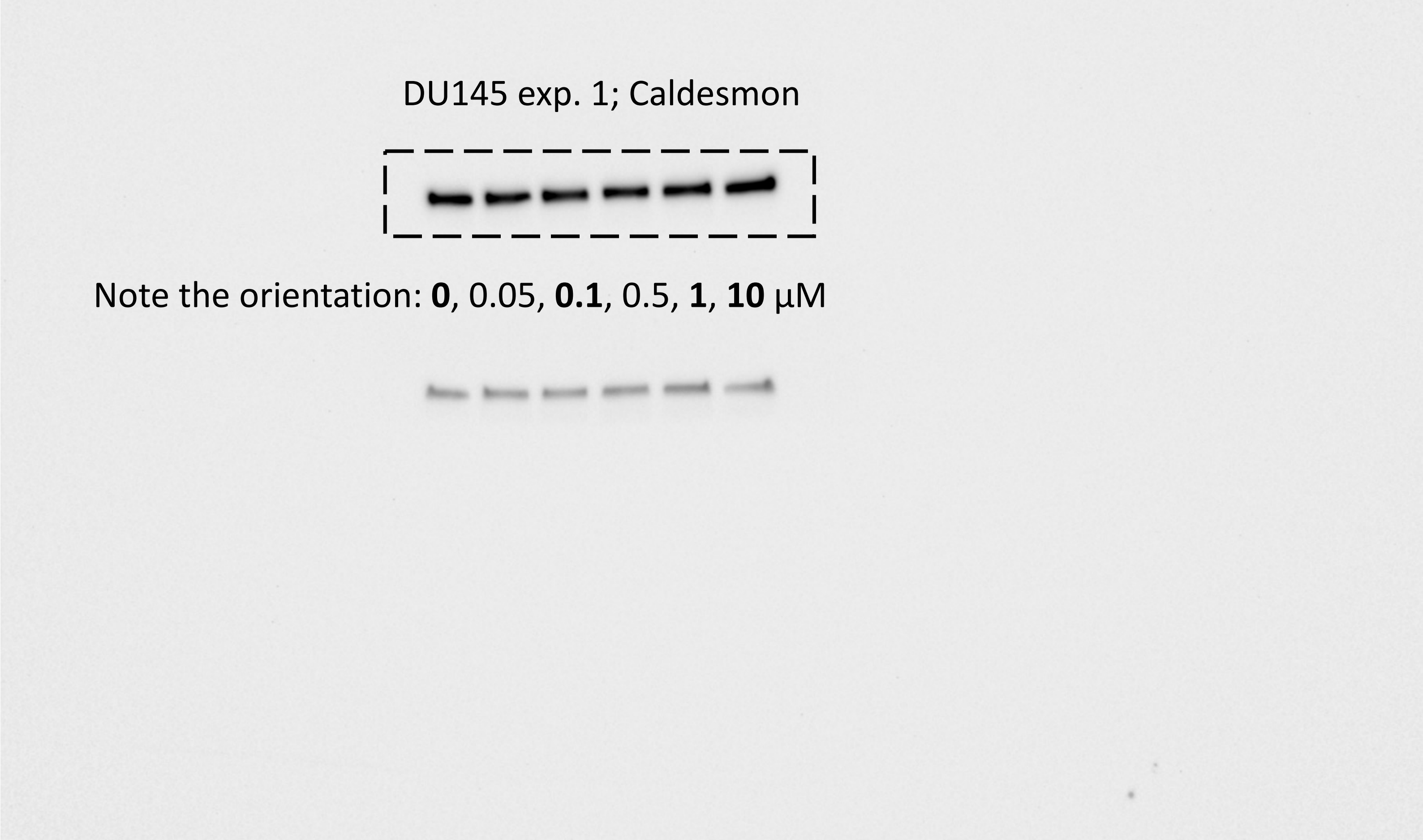

# Fig. 3F raw data for Western blot using anti-actin antibody (exp 1/3)

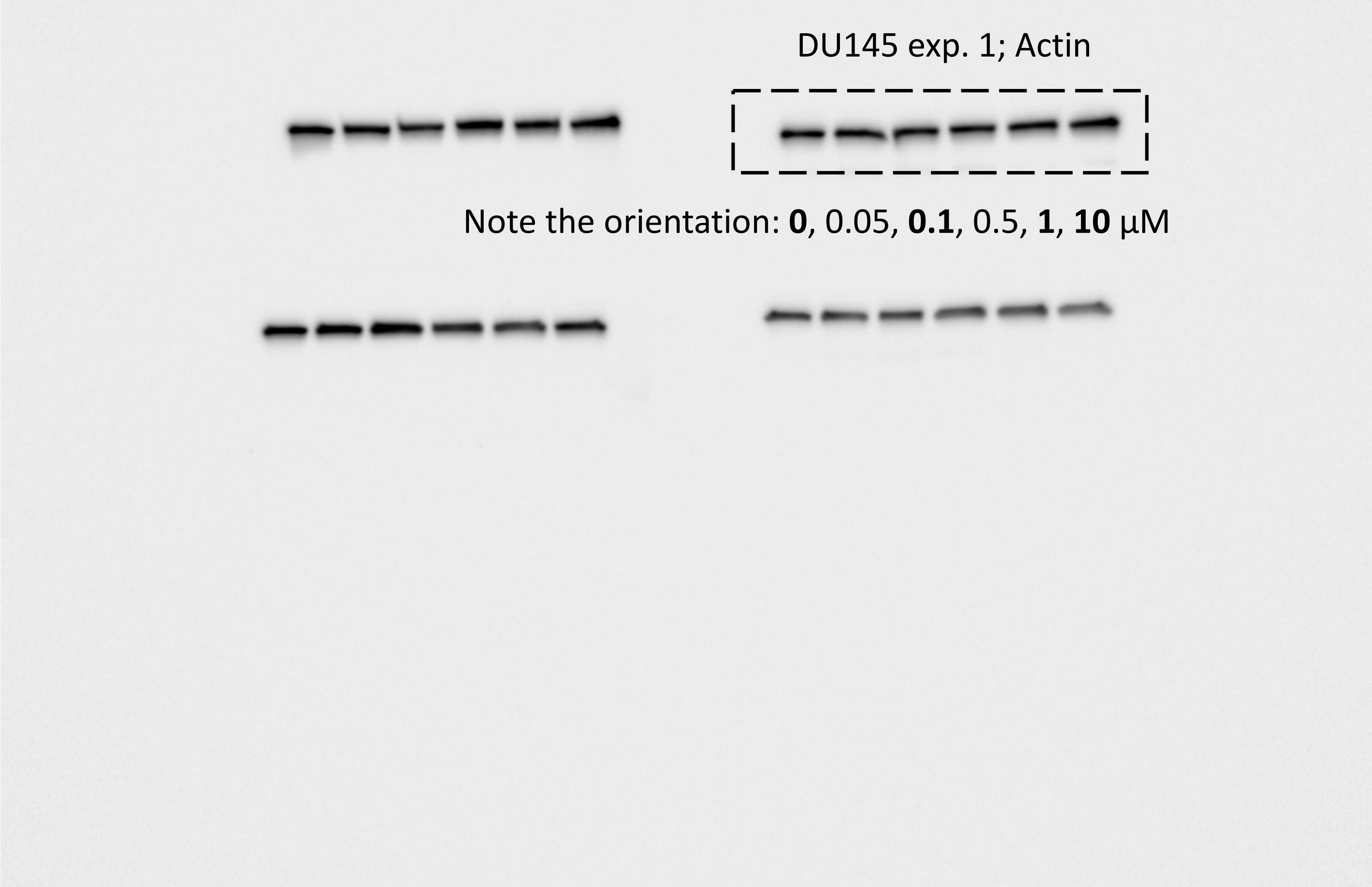

# Fig. 3F raw data for Western blot using anti-l-CaD antibody (exp 2/3)

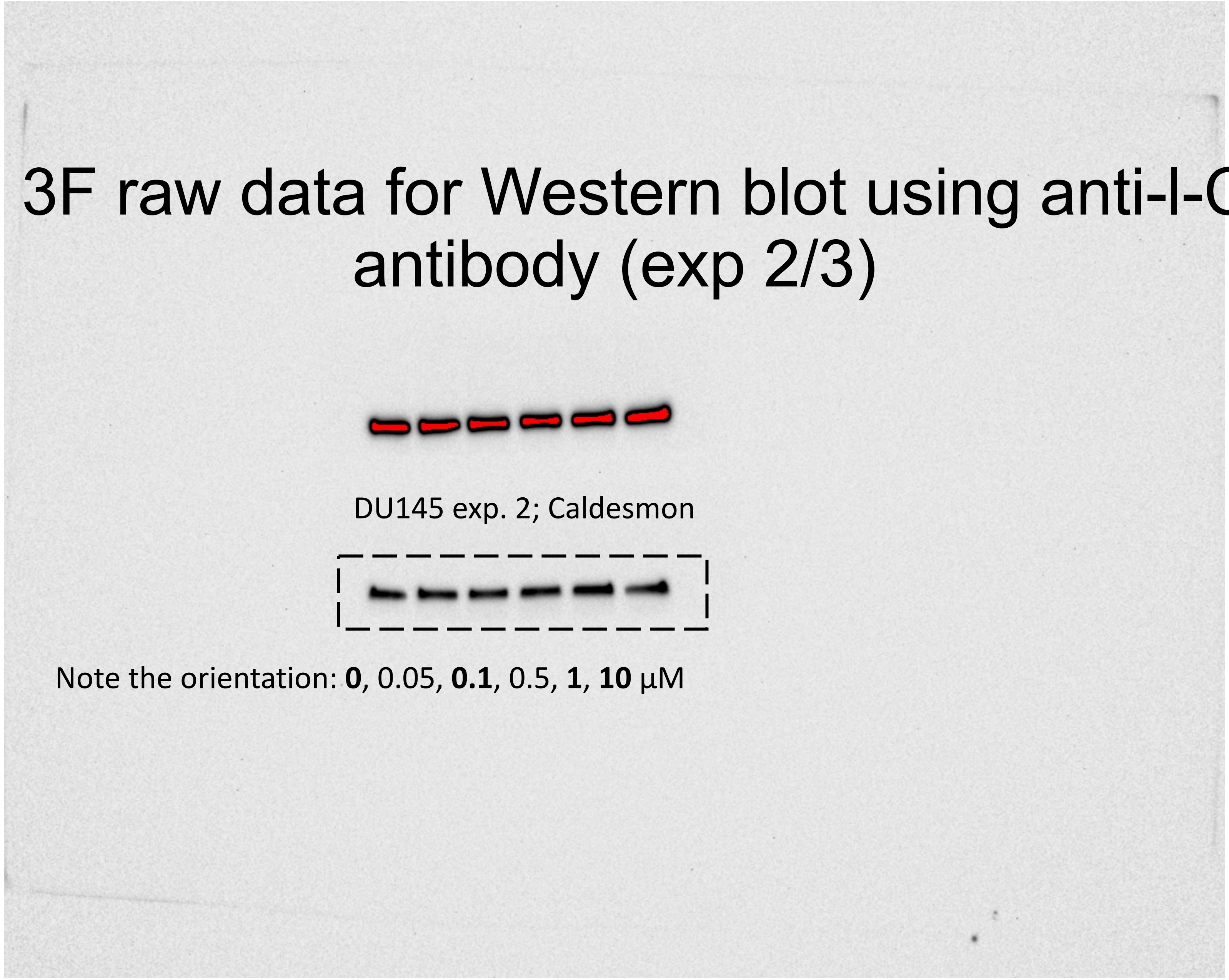

# Fig. 3F raw data for Western blot using anti-actin antibody (exp 2/3)

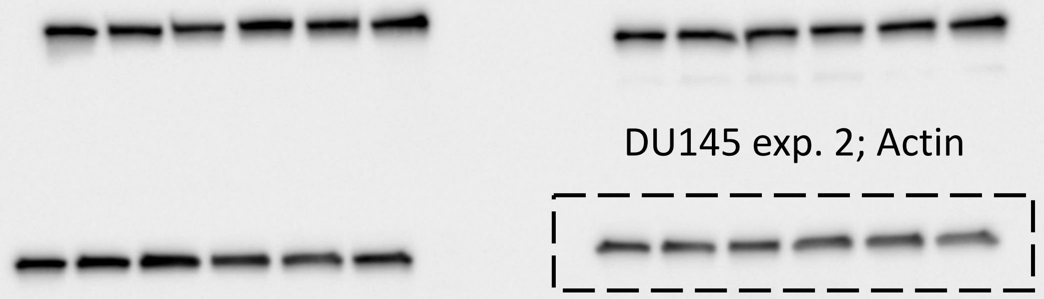

# Fig. 3F raw data for Western blot using anti-l-CaD antibody (exp 3/3)

DU145 exp. 3; Caldesmon

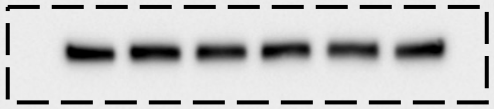

Note the orientation: **0**, 0.05, **0.1**, 0.5, **1**, **10** μM

# Fig. 3F raw data for Western blot using anti-actin antibody (exp 3/3)

DU145 exp. 3; Actin

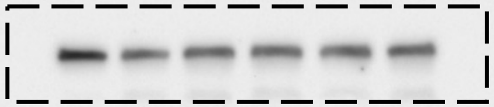

Note the orientation: **0**, 0.05, **0.1**, 0.5, **1**, **10** µM

# Fig. 5A raw data for Western blot using anti-l-CaD antibody (exps 1/3)

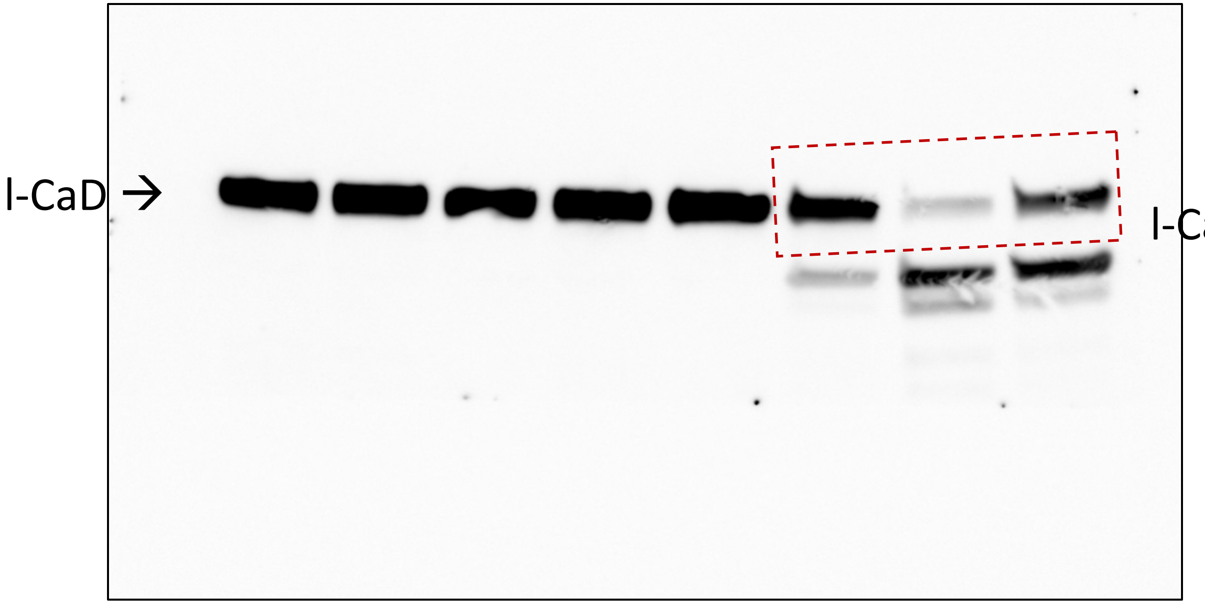


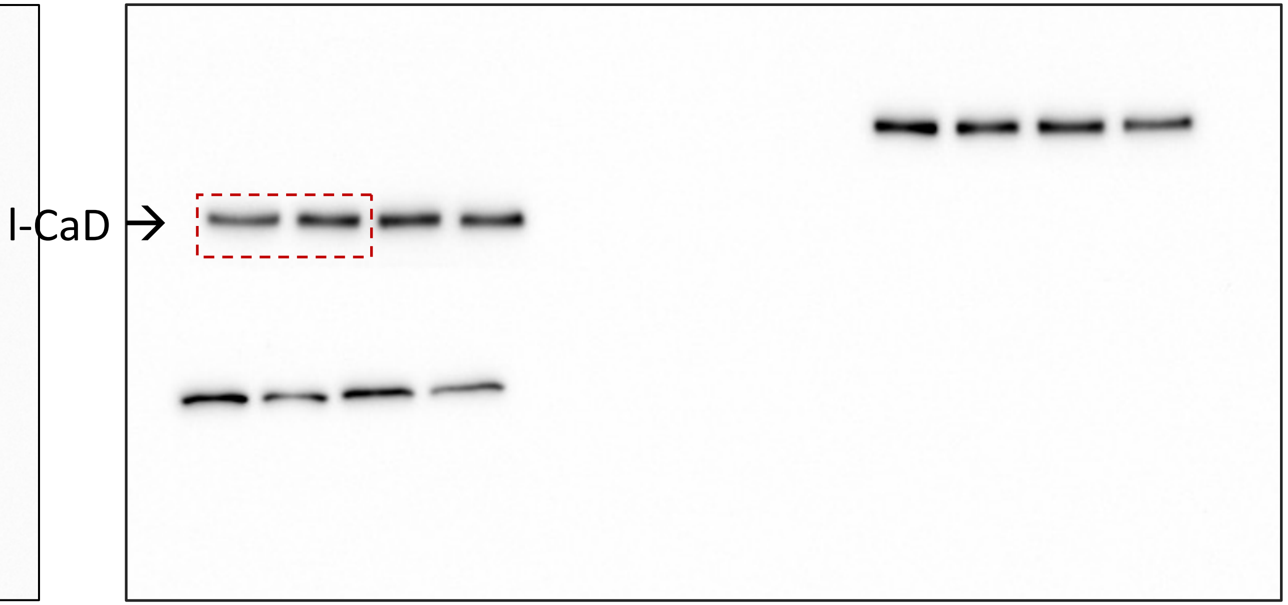

# Fig. 5A raw data for Western blot using anti-actin antibody (exps 1/3)

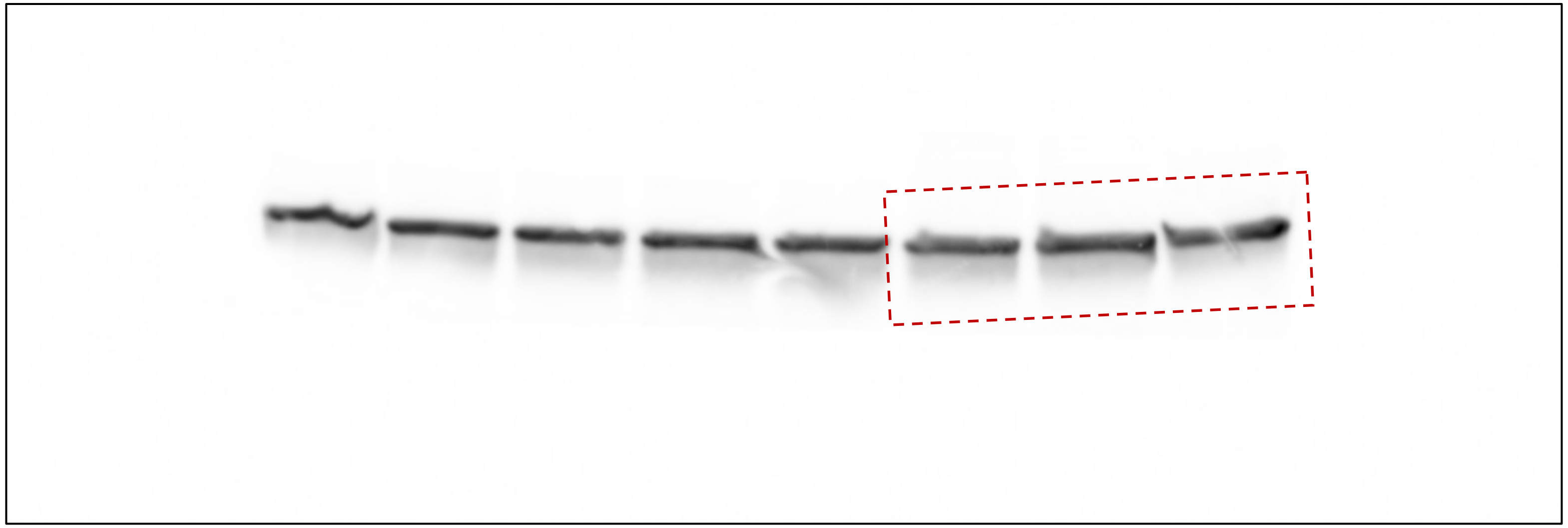

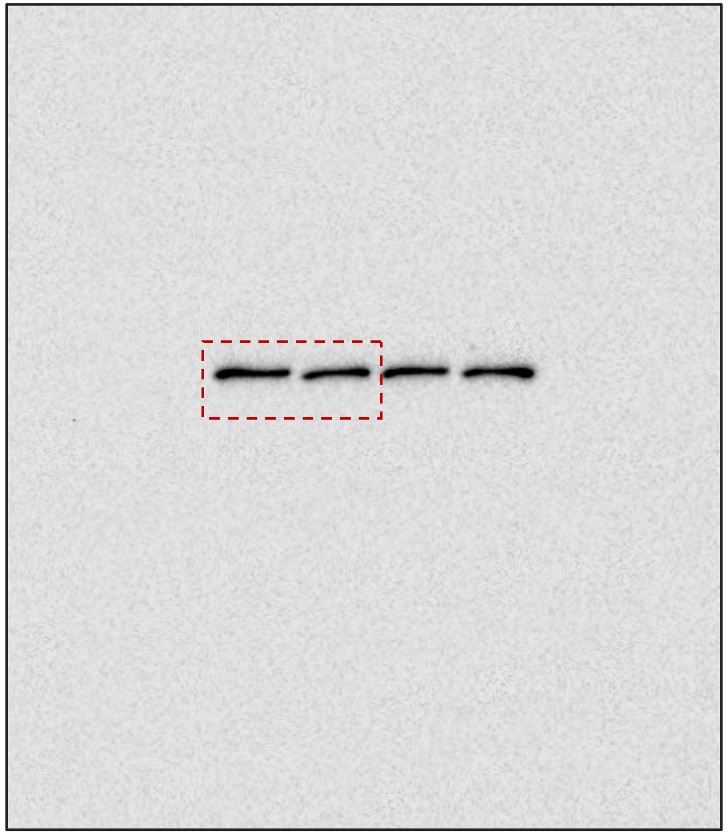

# Fig. 5A raw data for Western blot using anti-l-CaD antibody (exps 2/3)

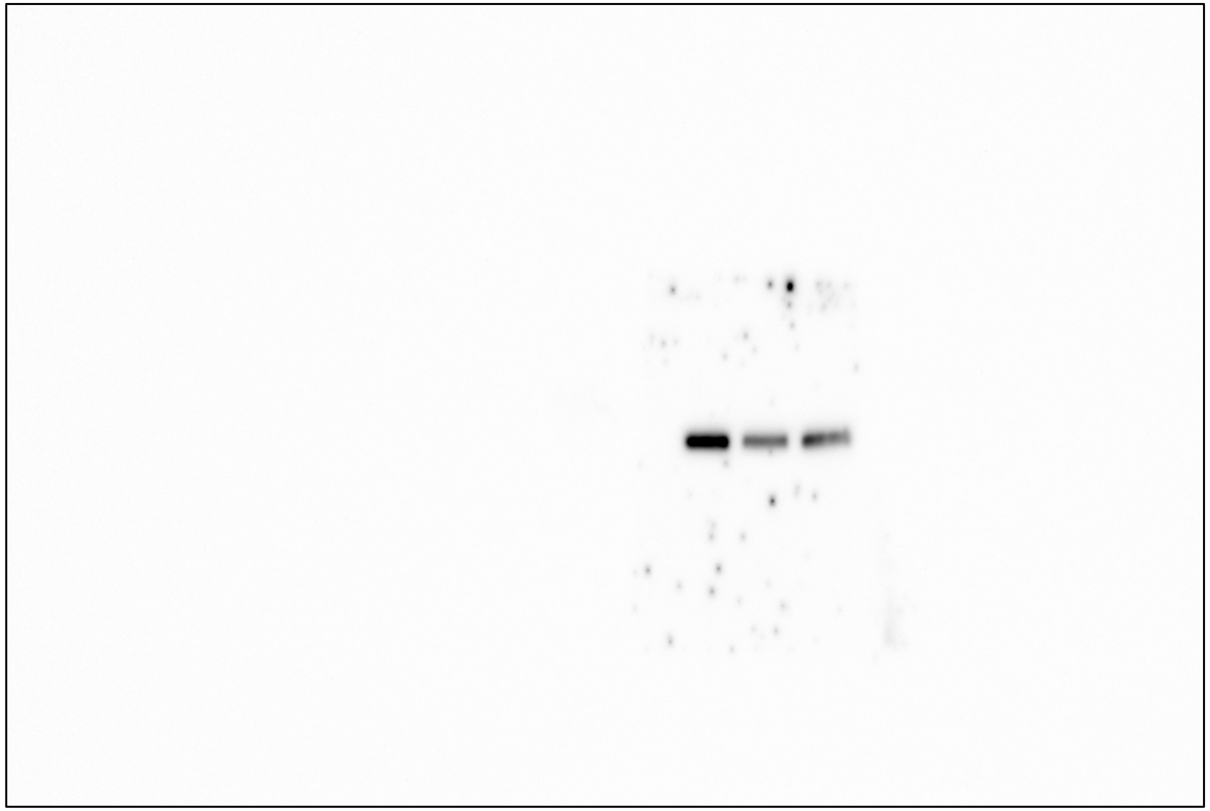

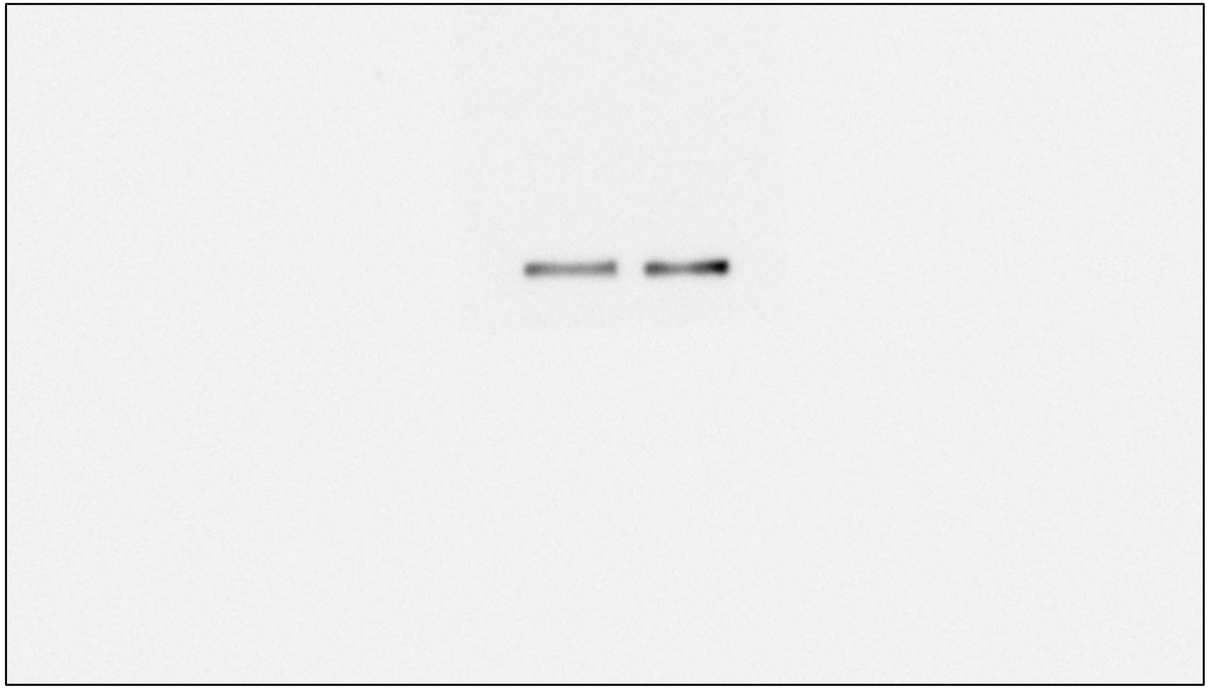

Fig. 5A raw data for Western blot using anti-actin antibody (exps 2/3)

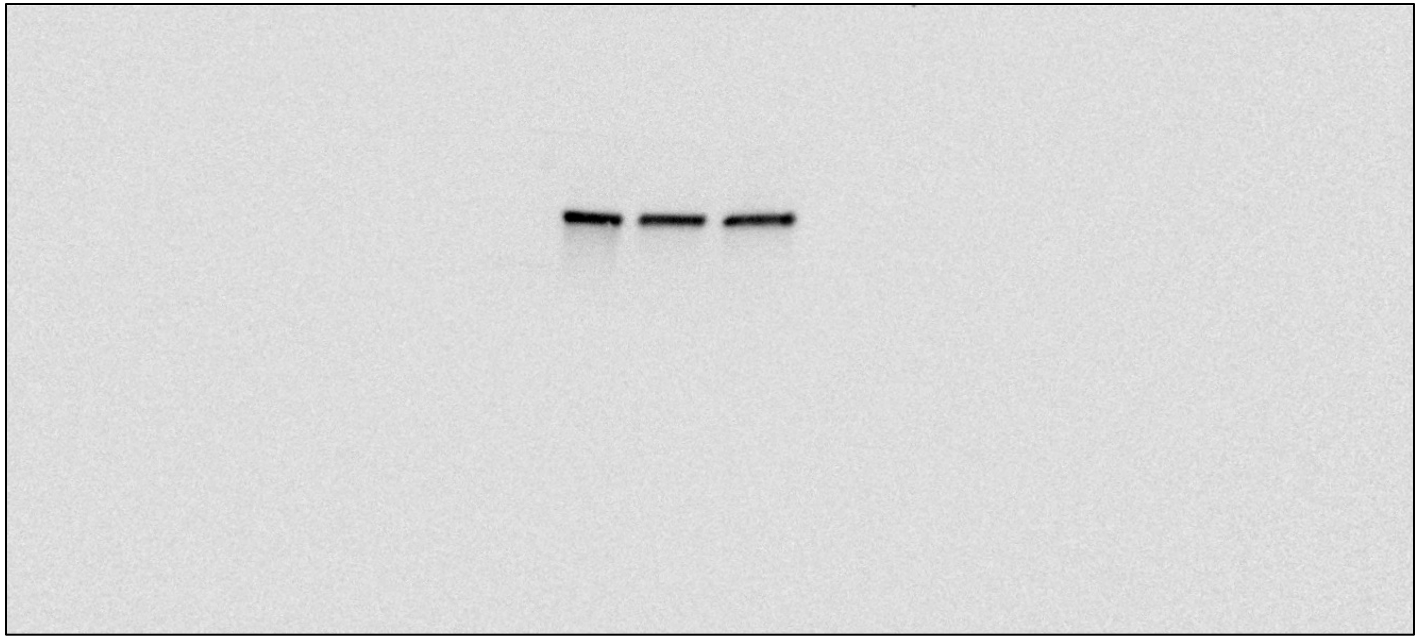

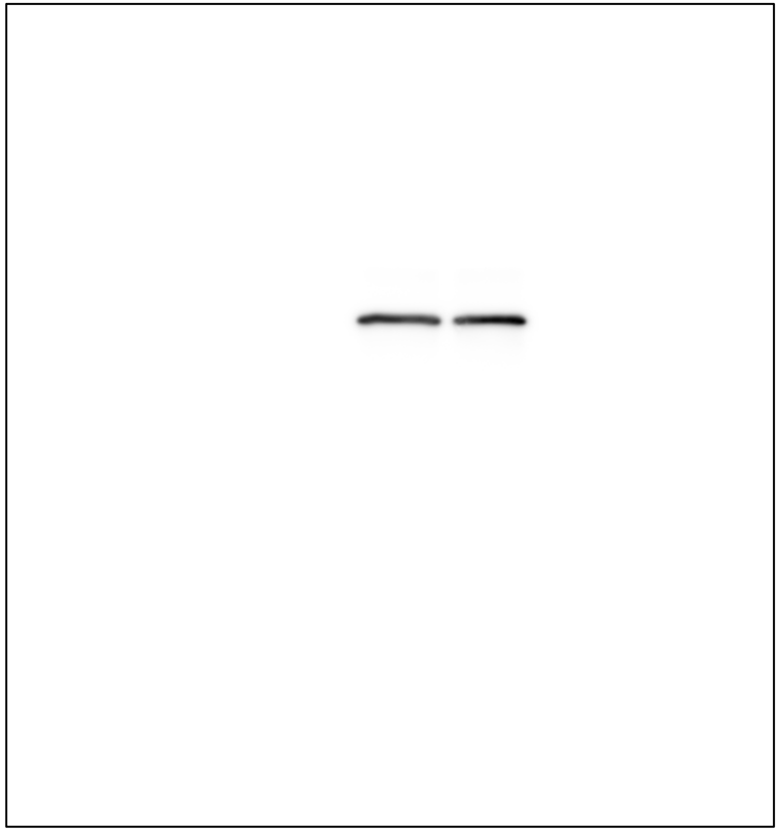

# Fig. 5A raw data for Western blot using anti-l-CaD antibody (exps 3/3)

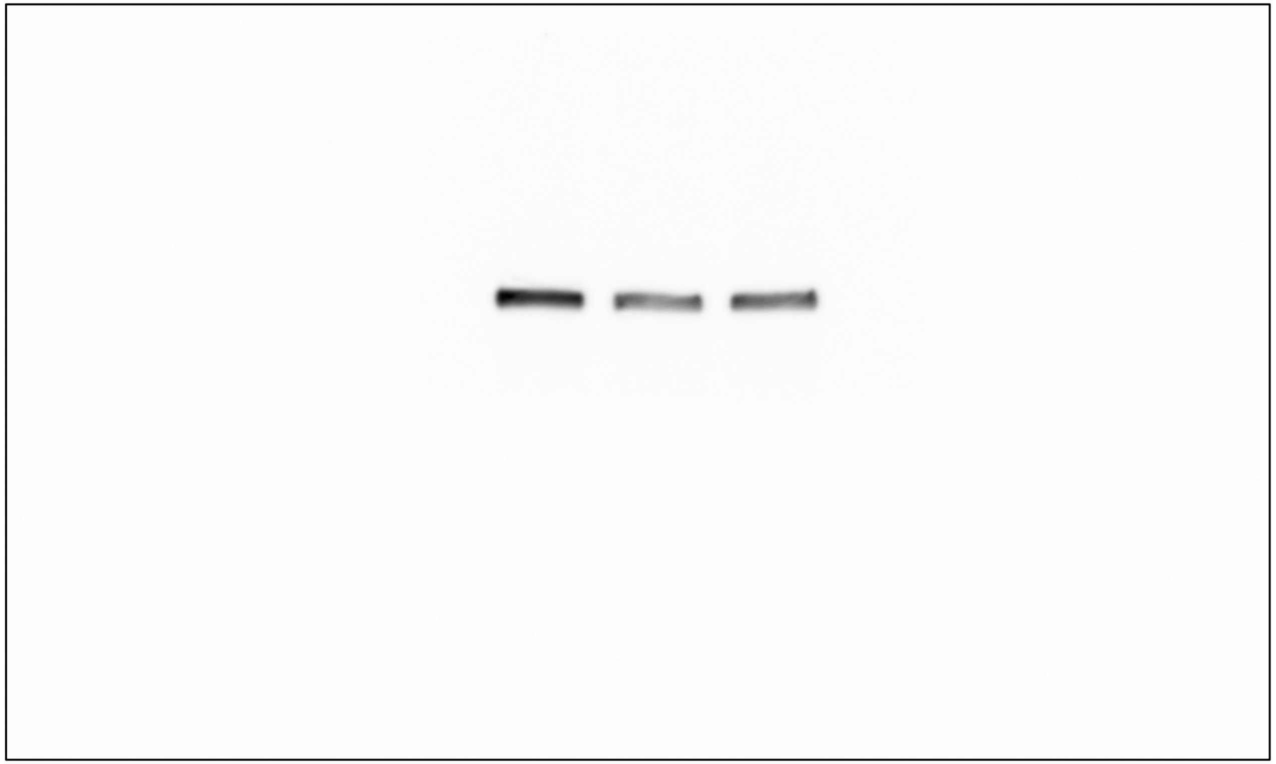

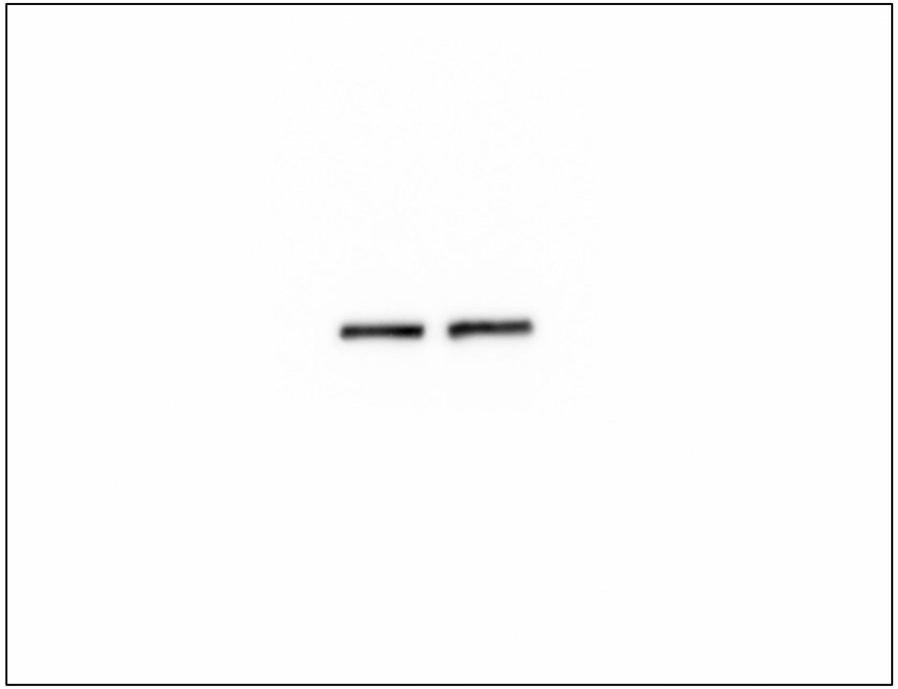

# Fig. 5A raw data for Western blot using anti-actin antibody (exps 3/3)

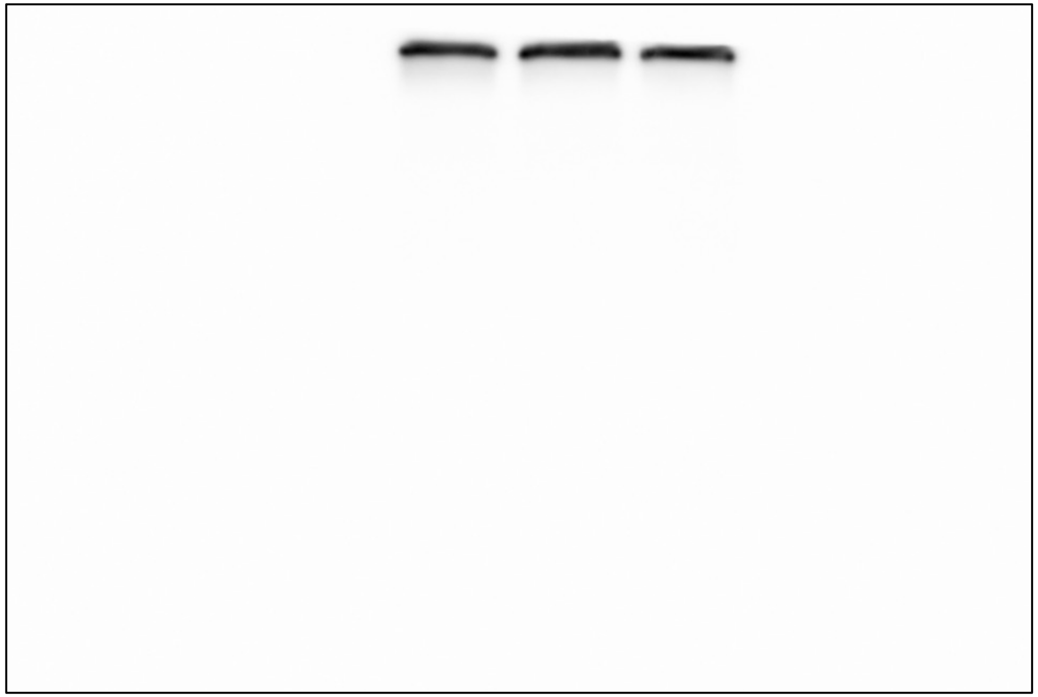

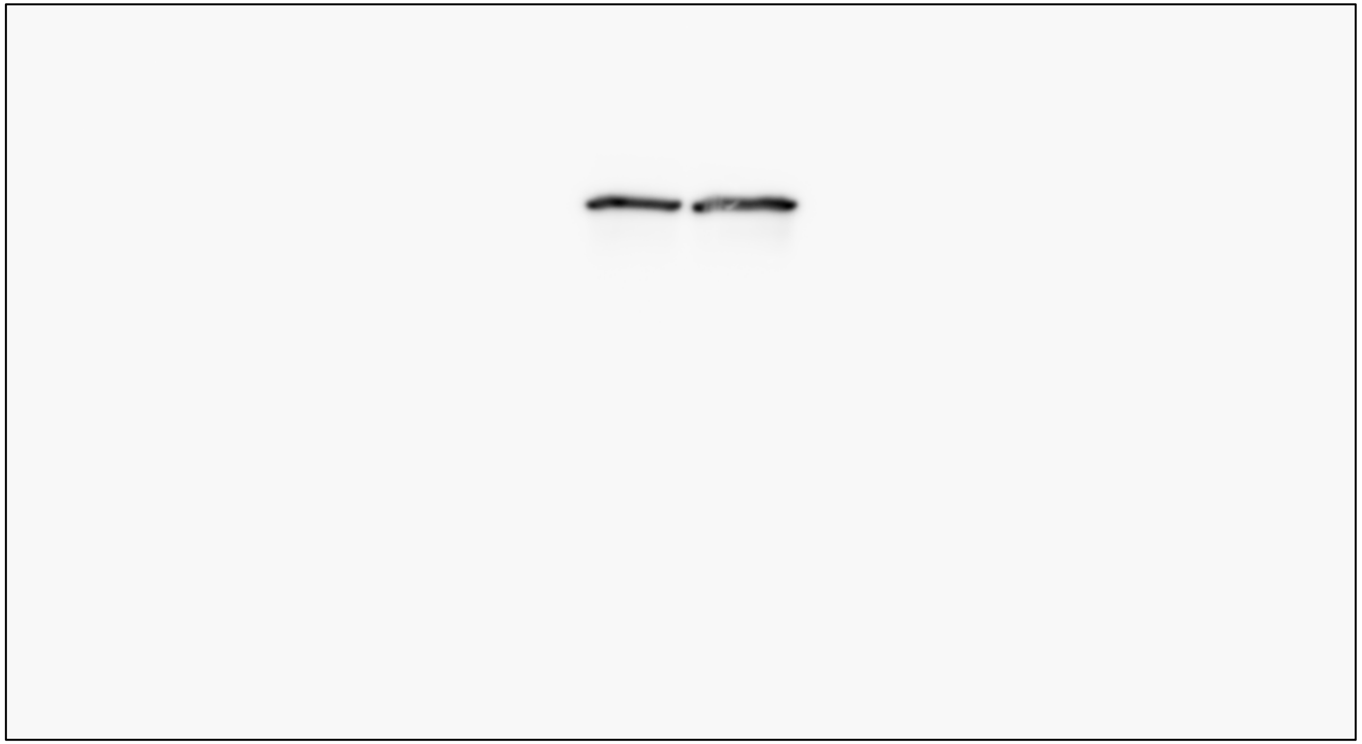

# Fig. 5G raw data for Western blot using anti-GR antibody

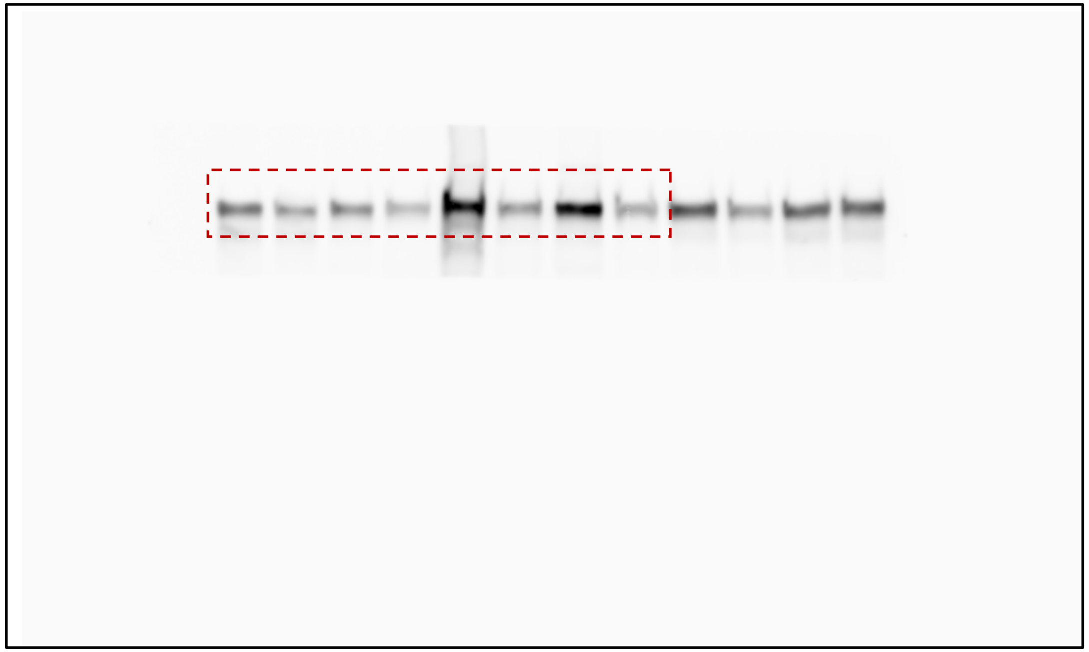

# Fig. 5G raw data for Western blot using anti-l-CaD antibody

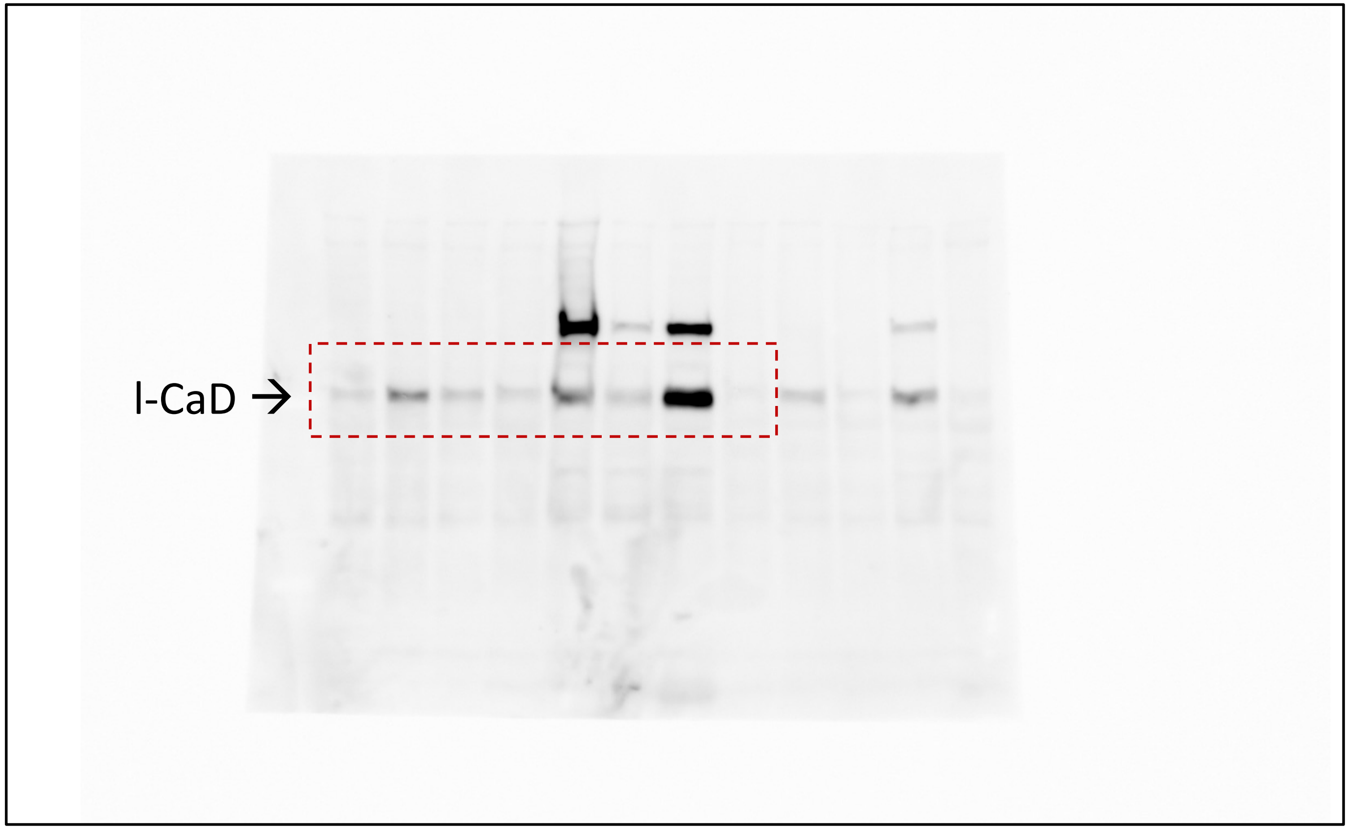

# Fig. 5G raw data for Western blot using anti-actin antibody

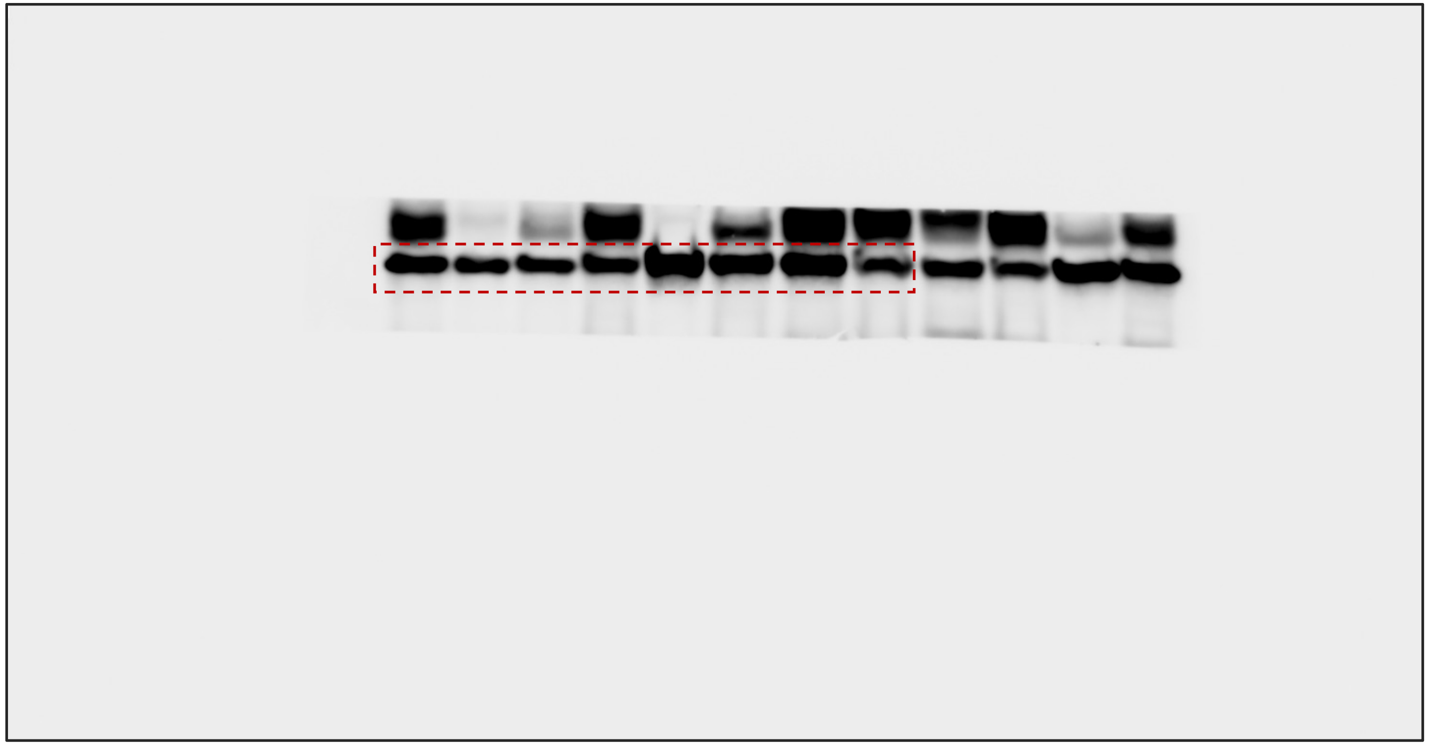

# Fig. S4B raw data for Western blot using anti-l-CaD antibody

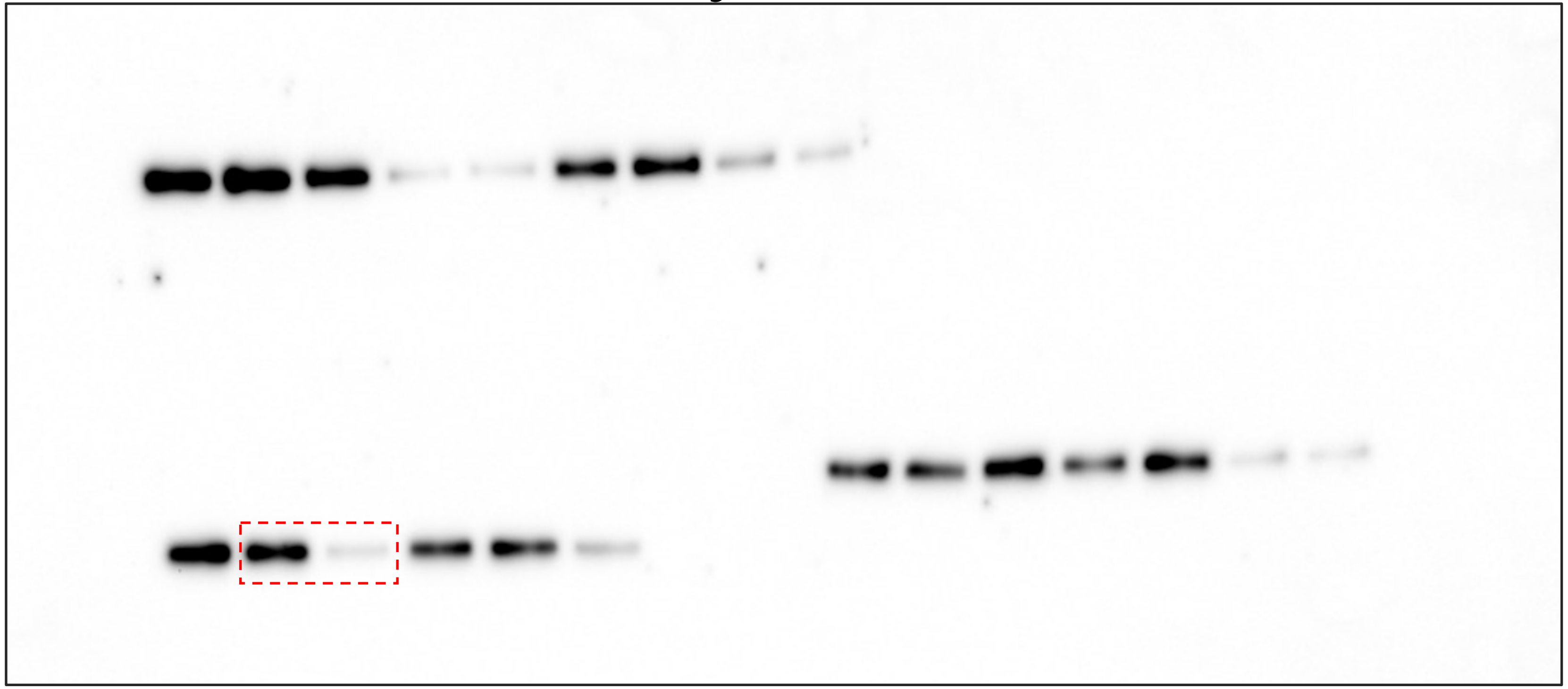

# Fig. S4B raw data for Western blot using anti-vinculin antibody

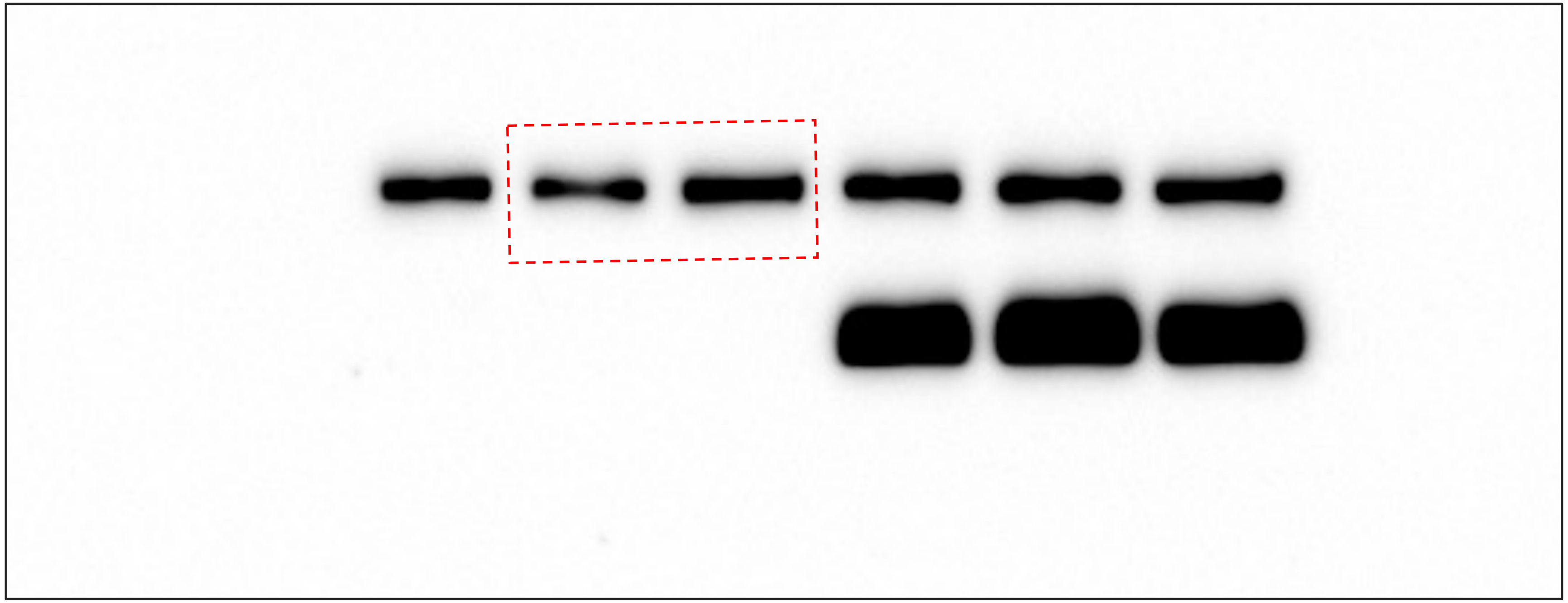

Supplement: Supplementary file 4 — Original Data File [file 41389_2023_485_MOESM4_ESM.pdf]
